# 4,6-*O*-Phenylethylidene Acetal Protected D-Glucosamine Carbamate-Based Gelators and Their Applications for Multi-Component Gels

**DOI:** 10.3390/gels8030191

**Published:** 2022-03-19

**Authors:** Pooja Sharma, Guijun Wang

**Affiliations:** Department of Chemistry and Biochemistry, Old Dominion University, Norfolk, VA 23529, USA; psharma@odu.edu

**Keywords:** carbohydrate, supramolecular gels, metallogels, organogelators, hydrogelators, chemiluminescence, carbamate, glucosamine, gel electrolyte

## Abstract

The self-assembly of carbohydrate-based low molecular weight gelators has led to useful advanced soft materials. The interactions of the gelators with various cations and anions are important in creating novel molecular architectures and expanding the scope of the small molecular gelators. In this study, a series of thirteen new C-2 carbamates of the 4,6-*O*-phenylethylidene acetal-protected D-glucosamine derivatives has been synthesized and characterized. These compounds are rationally designed from a common sugar template. All carbamates synthesized were found to be efficient gelators and three compounds are also hydrogelators. The resulting gels were characterized using optical microscopy, atomic force microscopy, and rheology. The gelation mechanisms were further elucidated using ^1^H NMR spectroscopy at different temperatures. The isopropyl carbamate hydrogelator **7** formed hydrogels at 0.2 wt% and also formed gels with several tetra alkyl ammonium salts, and showed effectiveness in the creation of gel electrolytes. The formation of metallogels using earth-abundant metal ions such as copper, nickel, iron, zinc, as well as silver and lead salts was evaluated for a few gelators. Using chemiluminescence spectroscopy, the metal–organic xerogels showed enzyme-like properties and enhanced luminescence for luminol. In addition, we also studied the applications of several gels for drug immobilizations and the gels showed sustained release of naproxen from the gel matrices. This robust sugar carbamate-derived gelator system can be used as the scaffold for the design of other functional materials with various types of applications.

## 1. Introduction

**Figure 1:**
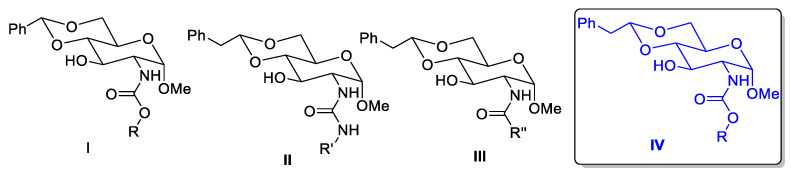
Structures of D-glucosamine derivatives that are effective LMWGs (**I**–**III**) and new derivatives prepared in the current study (**IV**).

Low molecular weight gelators (LMWGs) have gained much attention due to their versatile applications in the preparation of novel functional soft materials [1,2,3,4,5]. These materials have demonstrated potential in biomedical applications, pollutant removal, and optoelectronic devices, and for catalysis [4,6,7,8,9,10,11]. Various structural features have been carefully analyzed over the years in order to rationally design effective low molecular weight gelators [11,12]. Among the many classes of organic compounds used as the template for gelators, carbohydrates have emerged as interesting molecules for the preparation of these advanced materials due to their natural abundance, biocompatibility, and intrinsic chirality, which opens avenues for their desired modification [2,3]. The driving forces governing the formation of self-assembled networks by LMWGs are non-covalent in nature, such as hydrophobic interactions, hydrogen bonding, π–π, and CH–π interactions. Previously, various urea, amide, and carbamate derivatives of N-acetyl-D-glucosamine were synthesized, and their gelation properties were analyzed [13,14,15,16]. A variety of amides and ureas derived from 4,6-*O*-benzylidene acetal-protected D-glucosamine-formed gels in aqueous mixtures of ethanol and DMSO [13], and several amides were able to form gels for pump oil and engine oil [14]. Carbamate derivatives of 4,6-*O*-benzylidene acetal-protected glucosamine are versatile organogelators and hydrogelators as well [15,17]. As shown in Figure 1, similar amide derivatives synthesized using 4,6-*O*-(2-phenylethylidene) acetal-protected D-glucosamine (**III**) showed a good gelation trend in various solvents such as alcohols and aqueous mixtures of polar solvents such as ethanol, DMSO, and THF [14]. The methylene -CH_2_ unit introduced in the protecting group at the 4,6-position of the sugar ring in **IV** helped in facilitating hydrophobic interactions in comparison to **I**; and the carbamate functional group’s capability to participate in H-bonding is anticipated to be important for the self-assembling and hence gelation abilities of the synthesized analogs. 

Besides forming gels in organic solvents or in water, the formation of multiple component gels using these gelators for ionic liquids and electrolytes allows for their use as semiconductors and capacitors for energy and battery applications [18,19,20]. Gelators that have a response towards different anions are also reported and these have potential for practical applications in enzyme mimics and catalysis [21]. Gels formed in the presence of electrolytes such as tetrabutyl ammonium salts of halides may be useful as functional materials, such as gel electrolytes and for conductive media [22]. Similarly, ionogels can be useful for lithium ion batteries and other materials [23]. The formation of gel electrolytes from a sugar derivative is especially interesting since the materials will be more biocompatible.

Multiple component gels formed in the presence of metal ions, which are also called metallogels, have drawn an increasing amount of attention from researchers recently [20,24,25,26,27]. The introduction of metal ions to the gel matrix increases additional functions and can lead to materials that are therapeutic, conductive, and magnetic. Metallogels also showed to undergo catalytic reactions due to the redox activity of the metal ions trapped within the gel matrix [17,28,29]. For instance, a copper metal–organic hydrogel was used as a heterogeneous catalyst for the reaction of SO_2_ and CO_2_ with epoxides to produce cyclic 1,3,2-dioxathiolane-2 oxides and carbonates [30]. A recent multi-responsive silver organic gel has been prepared and showed responses towards light and chemicals [31]. Copper-based metal–organic gels (Cu-MOG) have been converted to a Cu-based metal–organic porous network (Cu-MOPN) with intrinsic oxidase and peroxidase-mimicking activities [32], and they are used as nanozymes for the colorimetry detection of dopamine [33]. A bimetallic copper(II)/cobalt(II) organic gel showed enhanced peroxidase-like activity and was applied for fluorescent detection of glucose [34].

Chemiluminescence (CL) is a unique phenomenon when light is emitted in response to a chemical reaction. Luminol-based CL is a powerful analytical method for reporting or detecting various analytes including hydrogen sulfide [35]. In a recent report, a metal–organic xerogel (MOX) was found to exhibit peroxidase activity to catalyze chemiluminescence of luminol, which was further used for dopamine detection [36,37]. A few recent reports have demonstrated the use of metal–organic gels (MOG) or metal–organic frameworks as enzyme mimics for CL systems [36,38,39,40]. An iron-based metal–organic gel showed enzyme-mimicking activities and was able to detect H_2_O_2_ released from Hela cells using luminol chemiluminescence [41]. 

Formation of multi-component gels will expand the scope of usefulness of LMWGs and because of the importance of metallogels and conductive gels, we are interested in designing and preparing novel self-assembled gels with these desired properties. To further expand the versatile gelation properties from sugar templates, in this study, we synthesized a set of thirteen carbamate derivatives and analyzed their gelation properties in various solvents, and with an emphasis of finding effective gelators for metal ions and electrolytes. 

## 2. Results and Discussion

**Scheme 1:**
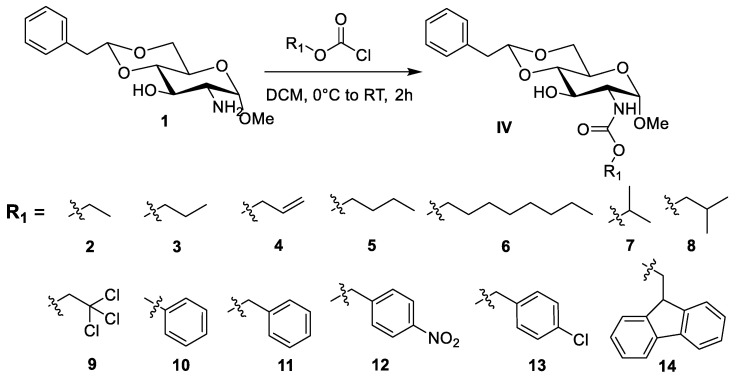
Synthesis of carbamate derivatives with general structure **IV**.

The synthesis of the carbamates is shown in Scheme 1. The glucosamine derivative **1** was synthesized in a few steps from *N*-acetyl-D-glucosamine according to literature procedures [14]. This intermediate was reacted with a series of commercially available chloroformates to afford N- linked carbamate derivatives **2**–**14**. We selected short-chain alkyl substituents (**2**–**6**) and branched derivatives (**7**–**9**), as well as aromatic derivatives (**10**–**14**). Compound **14** contains the F-MOC functional group, which often leads to good gelation performance. These carbamates were tested in a panel of solvents ranging from non-polar organic solvents such as hexane to polar solvents such as alcohols and aqueous mixtures of ethanol and dimethyl sulfoxide. Additionally, the compounds were also tested for their ability to form hydrogels as well as gelling pump oil. As shown in Table 1, compounds **2**–**14** displayed impressive gelation ability. The best performing solvent is glycerol and all compounds formed stable gels. Many compounds also formed gels in ethylene glycol, vacuum pump oil, and aqueous mixtures of organic solvents. These compounds did not form gels in ethanol and isopropanol. 

**Figure 2:**
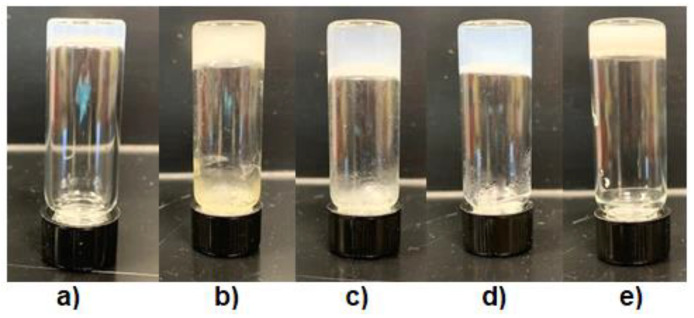
Opaque gels formed by several compounds: (**a**) compound **2** in DMSO:H_2_O (1:2) at 6.7 mg/mL; (**b**) compound **6** in DMSO:H_2_O (1:1) at 2.8 mg/mL; (**c**) compound **7** in H_2_O at 2.0 mg/mL; (**d**) compound **8** in EtOH:H_2_O (1:2) at 1.67 mg/mL; and (**e**) compound **11** in EtOH:H_2_O (1:2) at 3.3 mg/mL.

As shown in Table 1, compounds with linear aliphatic chains such as **5** and **6** formed gels in hexanes at 5.0 mg/mL and they also formed gels in six and seven other selected solvents. The branched alkyl derivatives **7** and **8** formed hydrogels at 2.0 and 10.0 mg/mL, respectively. Interestingly, the trichloro derivative compound **9** and the phenyl carbamate **10** were not able to gelate many solvents and they only formed gels in two solvents. However, benzyl and substituted benzyl derivatives **11** and **12** performed much better, forming gels in eight and seven solvents, respectively. The benzyl derivative **11** also formed a hydrogel at 0.4 wt% and the nitrobenzyl carbamate **12** showed great gelation ability by forming gels in aqueous mixtures of DMSO and ethanol at low concentrations. It formed a gel at 1.8 mg/mL in DMSO:H_2_O (1:2) and 2.8 mg/mL in EtOH:H_2_O (1:2). A similar reduction trend was observed with the chloro-phenyl derivative **13**; it became a less effective gelator and only formed gels in three solvents. The F-MOC derivative, on the other hand, was able to gelate several solvents. A majority of the gels appeared opaque and some were translucent. The representative gel photos are shown in Figure 2.

To compare the gelation performance of derivatives **IV** from this study with the general structures **I** reported before [14], a few examples in the same solvents are shown in Table 2. Compound **IV-a** formed more effective gels in EtOH:H_2_O (*v*/*v* 1:2) and DMSO:H_2_O (*v*/*v* 1:2) at concentrations of 6.7 and 4.0 mg/mL in comparison to compound **I-a,** which formed gels at 10.0 mg/mL in these solvent systems. However, the isobutyl derivative **IV-b** and **I-b** showed similar gelation tendencies, with the benzylidene derivative performed at lower concentrations in water and DMSO:H_2_O (*v*/*v* 1:2). With the benzyl carbamates, the gelation ability improved significantly for compound **IV-c**; it formed gels at lower concentrations at 3.3 mg/mL in EtOH:H_2_O (*v*/*v* 1:2) and 6.7 mg/mL in DMSO:H_2_O (*v*/*v* 1:2), and even formed a hydrogel at 4.0 mg/mL as compared to **I-c**, which did not form a hydrogel. The gelation properties of these carbamate derivatives showed overall enhancement possibly due to the increased hydrophobic methylene group “insertion” in the 4,6-protecting group. The calculated CLogP values for the butyl derivative **I-a** is 1.66 and 2.03 for compound **5**, which reflects the increased hydrophobicity of the benzyl derivative.

### 2.1. Gelation Mechanism by ^1^H NMR Studies

**Figure 3:**
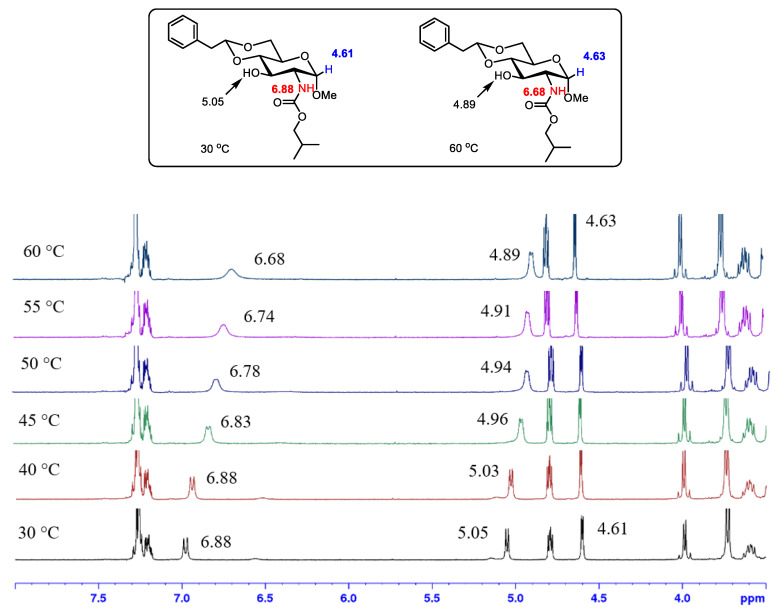
The ^1^H NMR spectra overlay of compound **8** from 30–60 °C at 8.0 mg/mL in DMSO-*d*_6_.

In order to probe the inter/intramolecular interactions, we carried out the ^1^H NMR experiment at different temperatures with compound **8** in DMSO-d_6_ (Figure 3). The full range ^1^H NMR overlaid spectra are shown in ESI Figure S1. As shown in previous studies for a similar system [14], typically both the -NH and O-H functional groups potentially participate in H-bonding. The carbamate NH signal of compound **8** showed a significant upfield shift from *δ* 6.88 to 6.68 ppm, corresponding to 30 °C and 60 °C, respectively. Similarly, the 3-hydroxyl group showed an upfield shift of 0.16 ppm from 5.05 to 4.89 ppm. These indicated the importance of hydrogen bonding from the carbamate NH and 3-OH groups intermolecularly. Interestingly, we also observed a slight downfield shift in the anomeric proton from *δ* 4.61 to 4. 63 ppm. Although the gelation mechanism varies with solvents, it is obvious that both the 2-NH and 3-OH functional groups play important roles in the gelation process. In comparison to the urea derivative **21** reported previously [14], the 3-OH also had a 0.16 ppm shift from *δ* 5.09 (30 °C) to 4.93 (60 °C) ppm. The urea’s 2-NH signal, however, showed a smaller chemical shift change of 0.07 ppm from 30 °C to 60 °C. This indicates that that the carbamate functional group played more important role during the gelation process since it offers both hydrogen bonding donating and accepting properties.

### 2.2. Metallogels Preparation and Analysis

The versatility of these gelators was further demonstrated by their ability to form gels in the presence of various metal salts. Several earth-abundant metal salts including Cu(OAc)_2_⋅H_2_O, CuBr_2_, CuSO_4_⋅5H_2_O, Zn(OAc)_2_⋅2H_2_O, Hg(OAc)_2_, Pb(OAc)_4_, NiCl_2_⋅6H_2_O, FeCl_3_⋅6H_2_O, FeCl_2_, and AgNO_3_ were used for the formation of metallogels. Compound **8** formed stable gels in the DMSO:H_2_O (*v*/*v* 1:5) solvent at 5.0 mg/mL concentration. This solvent was used for the metallogel formation for gelator **8**. The detailed gelation results are included in the ESI Figure S2 and Table S1. Besides compound **8**, we also tested the gelation performance of compound **11** with a few metal ions; it formed metallogels in the presence of the 1.0 equivalent (eq.) metal salt including Cu(OAc)_2_⋅H_2_O, ZnCl_2_, NiCl_2_⋅6H_2_O, and CoBr_2_ in the DMSO:H_2_O (1:5) mixture at 3.0 mg/mL concentration (Table S2). The photographs of the metallogels for compounds **8** and **11** are shown in ESI Figure S3a,b, respectively. 

**Figure 4:**
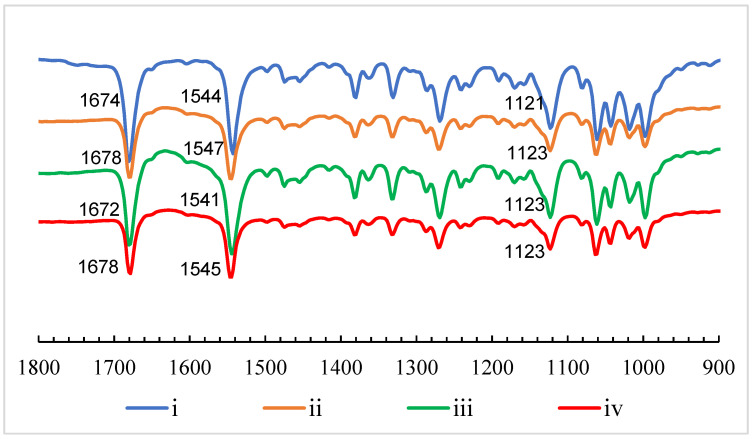
Overlay of IR spectra of compound **8** and its gels. (**i**) **8** as a solid. (**ii**) Gel formed by **8** in DMSO:H_2_O (1:5) at 6.0 mg/mL as control. (**iii**) Metallogel formed by **8** and Zn(OAc)_2_·2H_2_O (1.5 eq.) at 2.5 mg/mL. (**iv**) Metallogel formed by **8** and Cu(OAc)_2_·H_2_O (1.5 eq.) at 3.0 mg/mL.

**Figure 5:**
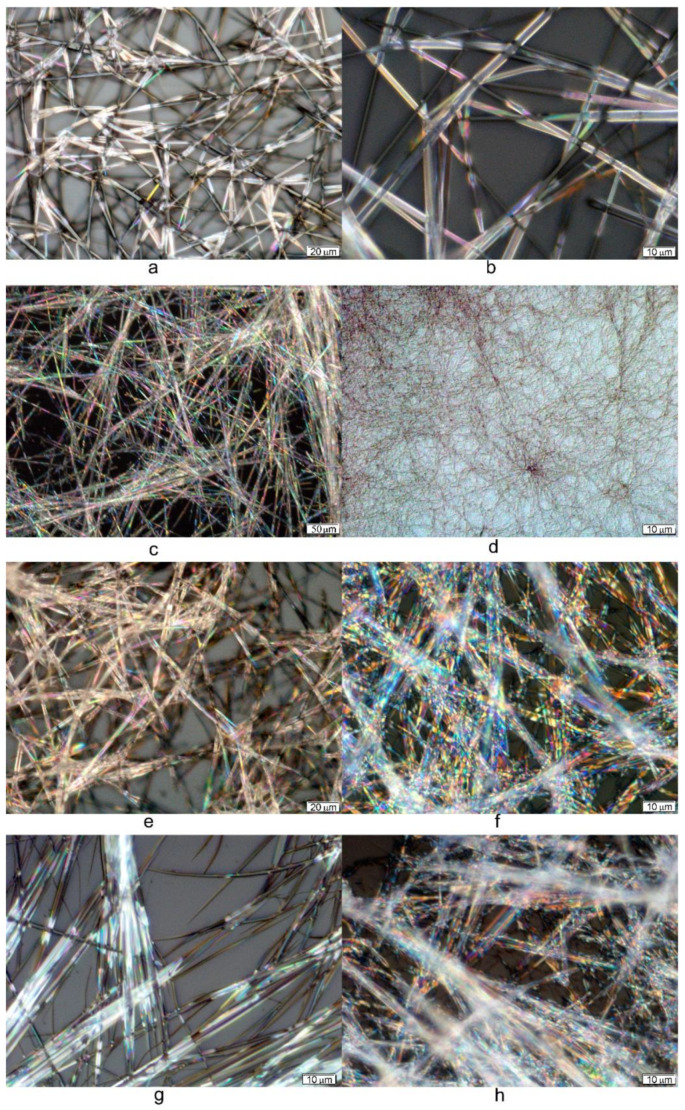
Optical micrographs of the gels formed by several compounds. (**a**,**b**) Compound **2** in DMSO:H_2_O (1:2) at 6.7 mg/mL; (**c**) compound **5** in hexane at 5.0 mg/mL; (**d**) compound **7** in H_2_O at 2.0 mg/mL; (**e**) compound **6** in DMSO:H_2_O (1:1) at 2.8 mg/mL; (**f**) compound **12** in DMSO:H_2_O (1:2) at 1.8 mg/mL; (**g**) compound **8** in DMSO:H_2_O (1:5) at 5.0 mg/mL; and (**h**) compound **8** with NiCl_2_·6H_2_O (1.5 eq.) in DMSO:H_2_O (1:5) at 2.5 mg/mL.

The summarized gelation results for compound **8** are shown in Table 3. Except for Hg (II), in the presence of the 1.5 equivalent of the metal ions to the gelator, opaque gels were obtained for all other metal salts with gelation concentrations ranging from 2.5 to 3.8 mg/mL. However, the compound showed an interesting sensing ability to detect Hg (II) ions as it did not form gels, in contrast to other metal ions. FTIR spectroscopy was used to analyze several gels with or without the metal ions. As shown in Figure 4, the binding of compound **8** with salts such as zinc acetate and copper acetate showed some changes in the peaks corresponding to functional groups such as C=O, C-O, N-H, and -OH. The full range FTIR spectra are available in ESI Figure S3c. These metallogels were characterized using an optical microscope, which revealed the presence of a three-dimensional network of long fibers as shown in Figure 5h.

### 2.3. Gel Morphology Characterization

**Figure 6:**
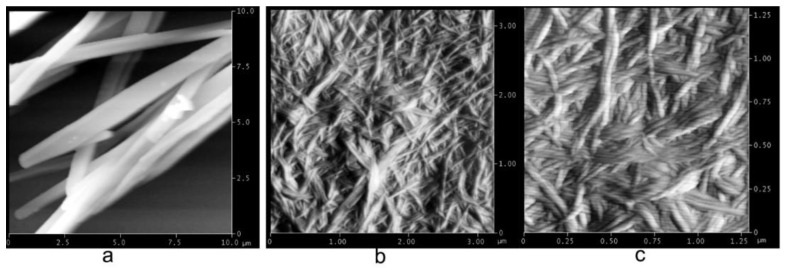
Atomic force microscope images of the gels formed by compounds **6** and **7**: (**a**) compound **6** in DMSO:H_2_O (1:1) at 2.8 mg/mL and (**b**,**c**) compound **7** in H_2_O at 2.0 mg/mL.

The gels obtained by several compounds were characterized using an optical microscope and atomic force microscope. Some of the representative images can be seen in Figure 5. Clearly, the presence of densely packed long fibers, a typical characteristic for supramolecular gels, was observed. The gels formed by compound **2** in DMSO:H_2_O (1:2) displayed tubular or cylindrical-shaped fibers (Figure 5a,b). The gel formed by compound **5** in hexanes at 5.0 mg/mL showed densely packed straight fibers and some flat ribbons (Figure 5c). The hydrogel formed by compound **7** showed a somewhat different morphology, which contains a much more flexible curly fibrous network with smaller diameters (Figure 5d). The gels formed by compound **6** in DMSO:H_2_O (1:1) showed the presence of a ribbon-like morphology similar to what was seen in Figure 6a. Additionally, the gel of **12** in DMSO:H_2_O (1:2) at 1.8 mg/mL showed thin planar ribbons and fibers. The gel of compound **8** in DMSO:H_2_O (1:5) exhibited a tubular and fibrous morphology (Figure 5g), and, after forming metallogels, the gel exhibited similar but more densely packed fibers or planar ribbon-like morphologies (Figure 5h). 

The AFM phase images of the gels by compounds **6** and **7** are shown in Figure 6. The gel formed by compound **6** in DMSO:H_2_O (1:1) at 2.8 mg/mL showed some individual long rods/tubule-types of assemblies (Figure 6a), while the hydrogel of compound **7** showed a uniform intertwined fibrous network (Figure 6b,c).

### 2.4. Rheological Analysis

**Figure 7:**
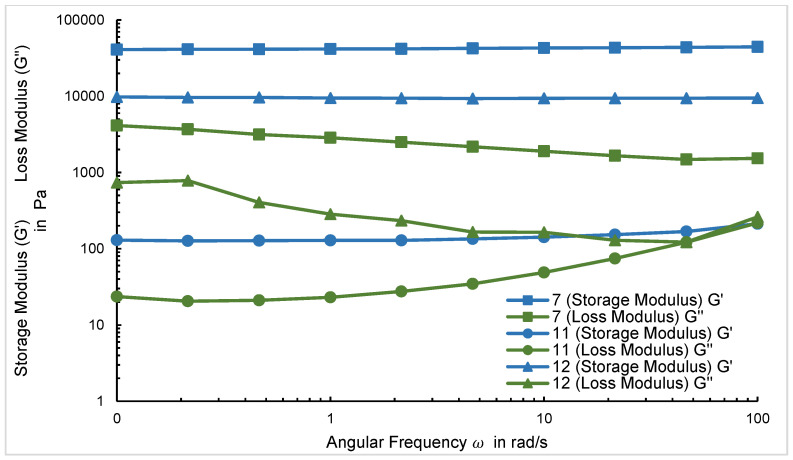
The rheological data for the gels formed by compound **7** in H_2_O at 2.0 mg/mL, **11** in glycerol at 1.3 mg/mL, and **12** in DMSO:H_2_O (1:2) at 1.8 mg/mL. The strain was 0.2% for all samples.

The viscoelastic strength of several gels was analyzed using rheology experiments. The gels for this study were aged 15–18 h followed by analyzing the linear viscoelastic region with the help of amplitude sweep experiments. For all gels, a 0.2% strain was identified in the linear range and was thus used for frequency sweep analysis. In general, the G′s for all gels studied were found to be higher than the loss moduli G″s. The average G′/G″ ratio was highest for the gel formed by compound **12** in DMSO:H_2_O (*v*/*v* 1:2) and lowest for the gel formed by compound **11** in ethylene glycol. The hydrogel of compound **7** at 2.0 mg/mL showed the highest values for G′, indicating high strength. The stacked rheological data graphs for these gels are shown in Figure 7. The results for the amplitude sweep and the tables for the rheology data can be found in SI Figure S4a–c and Table S3.

**Figure 8:**
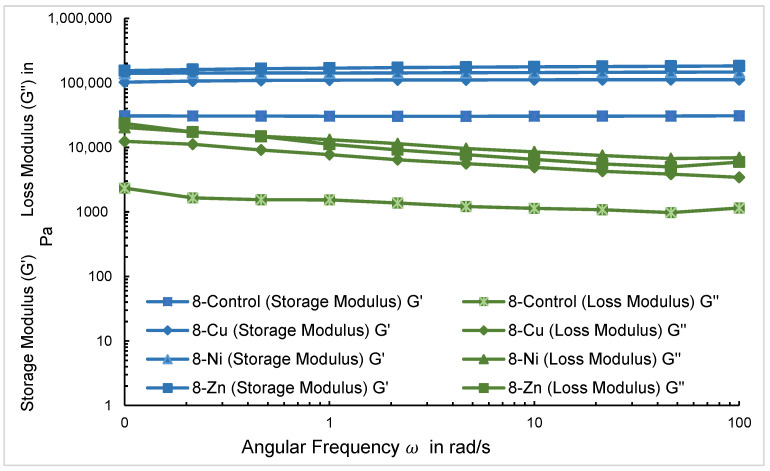
The rheological data for the gel formed by compound **8** in DMSO:H_2_O (1:5) and its corresponding metallogels. The strain was 0.2% for all samples.

The rheological properties of the metallogels were also analyzed using a similar procedure. The results for these gels are shown in Figure 8. In general, all gels were found to be stable under the tested angular frequency range. The mechanical strength of the metallogel formed by compound **8** with the 1.5 equivalents of zinc acetate salt at 2.5 mg/mL was found to be comparable with the control gel formed at 6.0 mg/mL in the DMSO:H_2_O (1:5) mixture in terms of their average G′/G″ values. The copper acetate and nickel chloride gels also displayed good mechanical strengths. The results for the amplitude sweep and the tables for the rheology data can be found in SI Figure S4d–g and Table S4.

### 2.5. Chemiluminescence Properties of the Metal–Organic Xero Gels with Several Metal Ions

**Figure 9:**
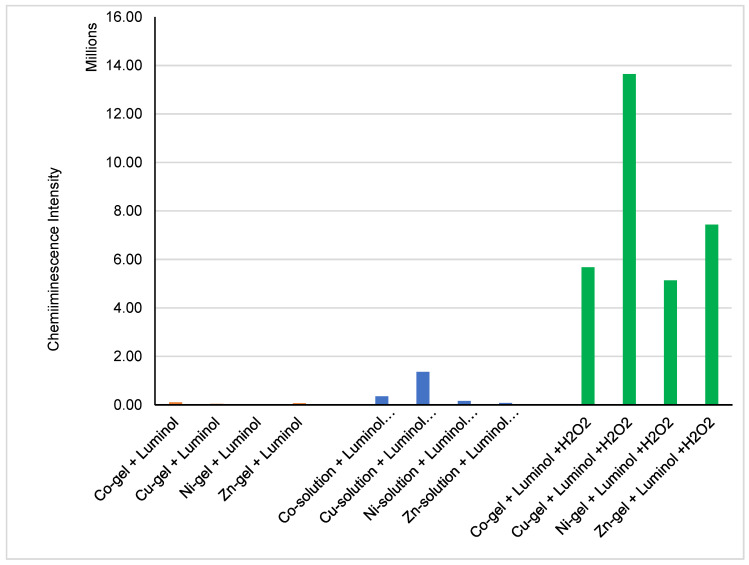
Chemiluminescence intensities (in Millions unit) of luminol with metal–organic xerogels of compound **11**. Controls are shown in orange and blue colors. The green-colored graphs are for the metal organogels: MOX (3.0 mg/mL in MilliQ water) + luminol (100 μL of 2.0 mg/mL solution in 10.0 mM NaOH) + H_2_O_2_ (100 μL of 0.15%).

To analyze the catalytic potential of the metallogels obtained from this study, the chemiluminescence of luminol was analyzed. It is well known that some transition metal cations can catalyze luminol CL reactions [42] and metal–organic gels have been utilized to both catalyze luminol chemiluminescence reactions and show enhanced CL intensities. The metallogels were prepared using compounds **8** and **11** with different earth metal salts including Cu(OAc)_2_·H_2_O, CoBr_2_, NiCl_2_·6H_2_O, and ZnCl_2_. The results are included in Figure S5 and Table S5a for compound **8**, and in Figure 9 and Table S5b for compound **11**.

As shown in Figure 9, the control panels include samples of metallogels of compound **11** (3.0 mg/mL in DMSO:H_2_O (1:5)) + luminol (100 μL of 2.0 mg/mL solution in 10 mM NaOH) without adding hydrogen peroxide. There was no CL observed with the control (orange). The second control (blue) included the solution of metal ions with luminol and hydrogen peroxide, and aqueous solutions of metal ions + luminol (100 μL of 2.0 mg/mL solution in 10.0 mM NaOH) + H_2_O_2_ (0.15%). Without adding gelators, the CL was almost non-detected, except for the copper solution, which showed weak luminescence. However, strong chemiluminescence (green) was detected with the metallogels and luminol together with 0.15% H_2_O_2_. This can be attributed to the abundance of the active metal sites and large surface area generated by the fibrous network of the gels, making them better catalysts in comparison to their corresponding solutions [36,43]. The xerogels obtained from this system were able to detect hydrogen peroxide with high intensity at concentrations as low as 15 mM. The copper xerogels showed the strongest CL, followed by zinc, nickel, and cobalt gels. 

### 2.6. Drug loading and Their Sustained Release Studies

**Figure 10:**
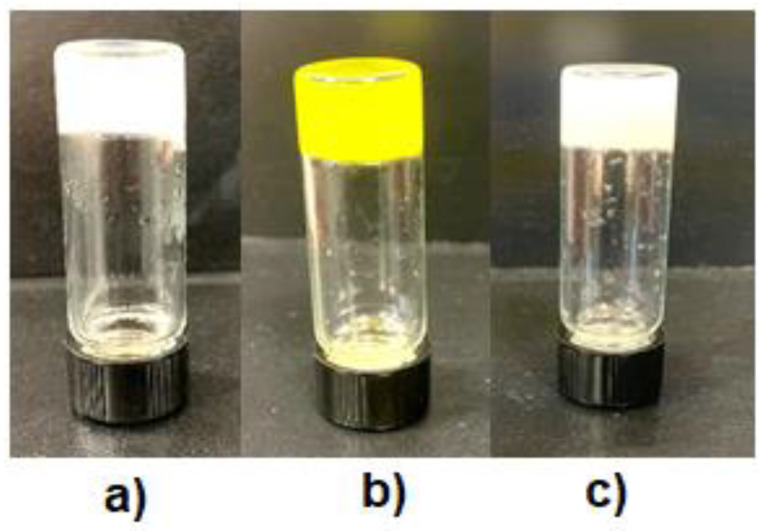
(**a**) Gel of compound **11** in 1.3 mL DMSO:H_2_O (1:10) loaded with 0.5 mg naproxen sodium. (**b**) Gel of compound **8** in 1.0 mL of DMSO:H_2_O (1:2) mixture with 0.1 mg vitamin B_2_. (**c**) Gel of compound **8** in 1.0 mL of DMSO:H_2_O (1:2) mixture.

**Figure 11:**
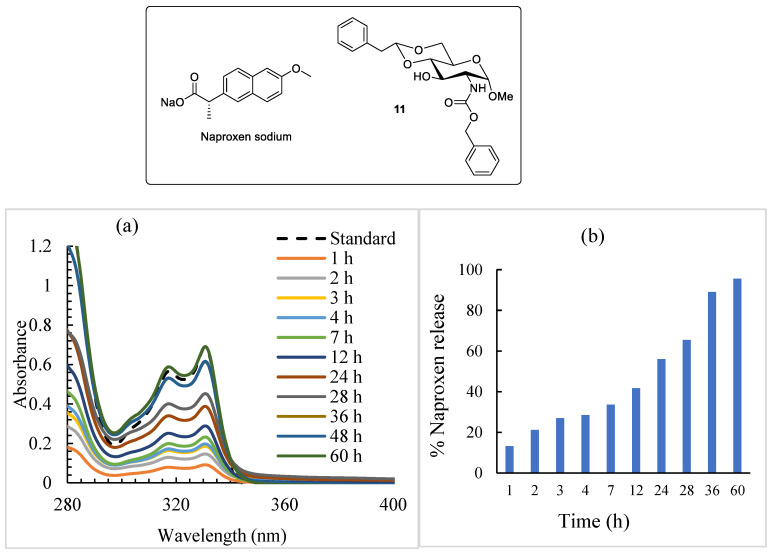
Results of naproxen release study using gelator **11**. (**a**) UV-vis spectra of naproxen sodium from the aqueous phase added on top of the gel at different times. (**b**) The cumulative percent release of the drug from the gel. The standard solution was prepared by dissolving 0.5 mg of naproxen sodium in 4.0 mL DMSO/H_2_O (*v*/*v* 1:10). The absorption at 330 nm was used to calculate the percentage of dye absorbed. Formula used: % drug released = [(A_standard_ − A_solution_)/A_standard_] × 100.

Another important feature of the carbohydrate-based gels is their ability to form gels in the presence of drug molecules. For our system, we were able to load 0.5 mg of naproxen sodium in the gel formed by **11** at 7.7 mg/mL. Figure 10 represents the images of gels formed by compound **11** with naproxen sodium and compound **8** in the presence of vitamin B_2_. Followed by these results, we carried out a study to analyze the sustained release of naproxen sodium from the gel to solution phase. This was carried out similarly to the literature [14] by monitoring the UV-vis absorptions of naproxen in the aqueous phase and the results are shown in Figure 11 and ESI File Figure S6. At 60 h, naproxen was about 90% released to the solution phase. In contrast to the release profile using the amide derivative, the carbamate gel showed more sustained release over time. 

### 2.7. Co-Gels with Electrolytes

**Figure 12:**
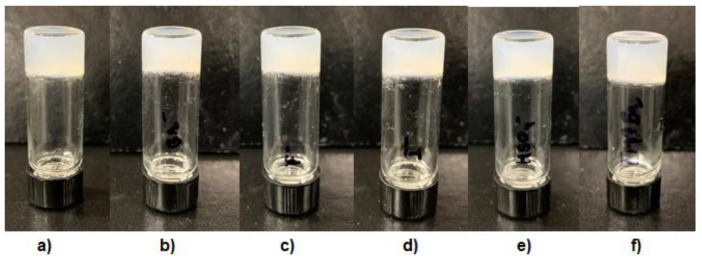
Gel photos for (**a**) compound **7** at 3.0 mg/mL as control and (**b**–**f**) its co-gels with various tetra-butyl ammonium salts at 3.0 mg/mL. Co-gels formed with 10.0 eq. (**b**) TBABr; (**c**) TBAF; (**d**) TBAI; (**e**) TBAHSO_4_; and (**f**) TMABr.

Since compound **7** formed a hydrogel, it was analyzed for its ability to form co-gels in the presence of electrolytes such as tetrabutyl ammonium (TBA) salts. The compound was able to form co-gels with up to the 10.0-equivalents salts such as TBABr, TBAF, TBAI, TBACl, TBAHSO_4_, and TMABr at 3.0 mg/mL concentration calculated with respect to the amount of gelator. Images of these co-gels are shown in Figure 12. 

The formation of gel electrolytes using low molecular weight gelators has theoretical and practical applications, as reported in the literature [44,45]. Gelator **7** formed stable gels in the presence of various TBA salts and these could have electrochemical applications. Therefore, the conductivity of the co-gels with TBABr and the solutions of TBABr salt alone was measured. The calibration curve of the conductivity of TBABr in DMSO:H_2_O (*v*/*v* 1:9) at various concentrations and conductivity data for gels formed by compound **7** at 3.0 mg/mL with various amounts of TBABr in DMSO:H_2_O (*v*/*v* 1:9) can be found in ESI Figures S7 and S8, and Table S6. The gel electrolytes showed increased conductivity in the gel form. For instance, co-gel formed by compound **7** with a 6.2 mM solution of TBABr showed a conductivity value of 1307 µS/cm, while the corresponding solution showed 700 µS/cm. These results illustrate the potential of small-molecule gelators in the formation of multiple-component gels, in this case, with tetra-alkyl ammonium salts that result in gel matrices with enhanced conductivity. 

## 3. Conclusions

Thirteen new C-2 carbamates derived from 4,6-*O*-phenylethylidene acetal protected D-glucosamine **1** were synthesized and characterized. These compounds were prepared in one step by reaction with the amine functional group in compound **1**. These compounds were found to be effective organogelators for several solvents. Three hydrogelators were also obtained from these compounds. The carbamates with linear or branched alkyl chains between three and eight carbons are the most versatile gelators, forming gels in at least six different solvents. The aromatic carbamate derivatives with a methylene group linker are also effective gelators. The gels were characterized using optical microscopy and atomic force microscopy for the morphology of the assemblies. The hydrogelators were selected to study for a few applications. They were shown to be able to form multiple component gels with metal ions, tetra-alkyl ammonium salts, vitamin B_2_, and naproxen. The gelators **8** and **11** formed metallogels with different metal ions and both the copper and cobalt xerogels demonstrated higher catalytic properties for chemiluminescence in the presence of hydrogen peroxide and luminol as compared to the corresponding metal salt solutions. Hydrogelator **7** also formed co-gels with many tetra-alkyl ammonium ions and exhibited higher conductivity in comparison to the solution. Gelator **11** formed stable gels with naproxen sodium, which showed sustained release over time for naproxen. These gelators and their various applications are expected to be useful models for designing soft materials that can function as sensors or conductive electrolytes. The hydrogelators can also be useful for drug delivery applications of other drug compounds. 

## 4. Materials and Methods

Reagents and solvents were used as they were received from the suppliers. All purification was conducted by flash chromatography using 230–400 mesh silica gel. The solvent systems used for chromatography and for the gelation test were all in volume ratios. NMR analysis was conducted using a 400 MHz Bruker NMR spectrometer. The molecular mass was measured using LCMS on an Agilent 6120B Single Quad Mass Spectrometer and LC1260 system or Shimadzu LCMS-2020 with ESI in positive ionization mode. Melting point measurements were carried out using the Stuart automatic melting point apparatus SMP40. Fluorescence emission spectra were obtained using the Shimadzu RF-6000 Spectro Fluorophotometer with excitation and emission bandwidths set at 5.0 nm and a scan speed of 600 nm/min. The gelation tests, optical microscopy, and AFM imaging were carried out following a literature report [46]. The instruments used for optical microscopy was the Olympus BX60M optical microscope and the Olympus DP73-1-51 high-performance 17MP digital camera with pixel shifting and Peltier cooling. AFM images were acquired using the Veeco Dimension 3100 Atomic Force Microscope. The tips used were Tap300-G silicon AFM probes with a resonant frequency of 300 KHz and a force constant of 40 N/m. Chemiluminescence data were acquired using the Varioskan LUX multimode microplate reader by Thermo Scientific from a 96-well adapter for plates without lids. The data was obtained in SkanIt software.

### 4.1. Optical Microscopy Studies

A small amount of the gel was placed on a clean glass slide using a micro-spatula and air-dried, which was then observed under an Olympus BX60M optical microscope and with the Olympus DP73-1-51 high-performance 17MP digital camera with pixel shifting and Peltier cooling. The imaging software used for image capturing was CellSens 1.11. 

### 4.2. Atomic Force Microscopy Studies 

AFM images were acquired using the Veeco Dimension 3100 Atomic Force Microscope. The tips used were Tap300-G silicon AFM probes with a resonant frequency of 300 KHz and a force constant of 40 N/m. The samples were prepared by spreading the gel on a glass plate with the help of a micro-spatula, which were then air-dried to obtain the corresponding xerogel viewed under the microscope.

### 4.3. Rheological Analyses 

The rheological properties of gels were investigated by Anton Parr MCR 302 with RheoCompass software. The cone geometry is a 25 mm Peltier plate for both and with a gap of 0.1 mm for the Anton Parr Rheometer. The experimental temperature was 25.0 °C and the sample was subjected to amplitude sweep for an oscillation strain from 0.1% to 10%. A frequency sweep was then performed for the sample in the range of 0.1 to 100.0 rad/s for an angular frequency at 0.2% strain. The results were expressed as the storage modules (G′) and loss modules (G″) as a function of the angular frequency.

### 4.4. Preparation of Gels with Tetra-Alkyl Ammonium Salts and Conductivity Study 

In a one-dram vial, 3.0 mg of gelator **7** and 1.0 mL of DI water were added. This mixture was heated until all solids had dissolved, which was then allowed to cool down to rt for 15 min. The formation of the gel was tested by inverting the vial. This hydrogel was used as the control (3.0 mg/mL). To prepare the gels with different anions, the desired amount of TBA salt was added. The mixture was then re-heated and cooled to test the formation of the gel. 

For the conductivity studies, the gels were prepared using compound **7** (30.0 mg, 1.0 eq.) in 1.0 mL of DMSO. To this solution, warm water (9.0 mL) was added through a syringe to the solution. The resulting mixture was allowed to cool to rt until gelation was observed. To this gel, TBABr (10.0 mg, 0.5 eq.) was added for the measurement of conductivity. Similarly, gels with 1.0 eq. (20.0 mg), 1.5 eq. (30.0 mg), and 3.0 eq. (60.0 mg) of TBABr were prepared and tested for conductivity. Details of the amounts used for each gel are available in Table S7. 

### 4.5. Metal–Organic Gels for Chemiluminescence Study

In a one-dram vial, gelator **11** (3.0 mg, 0.007 mol, 1.0 eq.) and metal salt (1.0 eq.) were added. Then, the DMSO:H_2_O (1:5) mixture (0.2 mL) was added into the vials. The mixture was heated to form a homogeneous solution and allowed to cool to room temperature to form gels. Subsequently, metal−organic gels were observed within 15 min under ambient conditions. Metal–organic Xerogels (MOX) were further acquired after removing the solvent of MOGs under air. To investigate the luminol−MOXs CL system, 100 μL of the solution of MOX (3.0 mg/mL premixed in Millipore-grade water) was added in a 96-well plate. Then, 100 μL of the luminol solution (2.0 mg/mL in 10.0 mM NaOH solution) was injected, followed by 100 μL of the H_2_O_2_ solution (0.15%), and both the CL profile and intensity were measured.

### 4.6. Naproxen Release Study

A gel was prepared in a one-dram vial using compound **11** (15.0 mg), 0.5 mg of naproxen sodium, and 2.0 mL of DMSO/H_2_O (*v*/*v* 1:10). After a stable gel formed and the gel was left undisturbed for 15 min, 2.0 mL of DMSO/H_2_O (*v*/*v* 1:10) was added to the top of the gel carefully. The naproxen release from the gel was monitored by UV absorption at intervals by transferring the supernatant with a pipet to a cuvette and, after each measurement, the aqueous phase was carefully transferred back to the vial and placed on top of the gel again until the next measurement. The UV spectra of 0.5 mg of pure naproxen in 4.0 mL DMSO/H_2_O (*v*/*v* 1:10) was also recorded as standard. Images of the gels can be found in ESI File Figure S6.

### 4.7. Synthesis of Carbamate Derivatives

Headgroup 4,6-*O*-(2-phenylethylidene)-protected glucosamine derivative **1** was synthesized according to the literature procedure [14]. The general method for the carbamate synthesis and detailed synthesis of compound **2** is provided, and only the amounts of the reagent and characterization data are given for other compounds. 

### 4.8. General Procedure for the Preparation of Carbamate Compounds

Headgroup **1** (1.0 eq.) was dissolved in anhydrous DCM at 0 °C; Et_3_N or DIEA (1.1–1.5 eq.) was added; and the reaction mixture was held at 0 °C for 15 min under an anhydrous N_2_ atmosphere. Then, the desired chloroformate (1.1–1.5 eq.) was added to the reaction mixture, which was then stirred at RT until the completion of the reaction, after which the mixture was diluted with CH_2_Cl_2_, washed with water and brine, dried over Na_2_SO_4_, concentrated, and purified by flash column chromatography on silica gel using 0–3% MeOH in DCM to give the pure product carbamates. 

**Synthesis of compound 2.** In a 50 mL round bottom flask, compound **1** (75 mg, 0.25 mmol, 1.0 eq.) was dissolved in anhydrous DCM (5.0 mL). Triethyl amine (35 µL, 0.25 mmol, 1.0 eq.) was added next and the reaction mixture was kept at 0 °C for 15 min under an anhydrous N_2_ atmosphere. After this, ethyl chloroformate (26 µL, 0.27 mmol, 1.1 eq.) was added and the mixture was allowed to warm gradually to room temperature and stirred until the completion of the reaction. The crude product was purified by column chromatography using 0–1% MeOH in DCM to afford a yellowish solid (81.6 mg, 0.22 mmol, 89%) as the desired product. (R_f_ value in 5% MeOH/DCM = 0.54) M.P. = 136.0–137.0 °C; ^1^H NMR (400 MHz, CDCl_3_) *δ* 7.31–7.19 (m, 5H), 5.12–4.93 (m, 1H, -NH), 4.76 (dd, *J* = 4.0, 6.3 Hz, 1H, H-7), 4.67 (d, *J* = 3.2 Hz, 1H, H-1), 4.21–4.07 (m, 3H, H-6b, H-10), 3.91–3.77 (m, 2H, H-2,3), 3.70–3.61 (m, 1H, H-5), 3.48 (dd~t, *J* = 10.3 Hz, 1H, H-6a), 3.39 (s, 3H, 3H-9), 3.40–3.29 (m, 1H, H-4), 3.04 (dd, *J* = 14.2, 3.9 Hz, 1H, H-8a), 2.95 (dd, *J* = 14.2, 6.3 Hz, 1H, H-8b), 1.26 (t, *J* = 7.1 Hz, 3H, 3H-11); ^13^C NMR (100 MHz, CDCl_3_) *δ* 157.1, 136.1, 129.7, 128.3, 126.6, 102.8, 99.1, 81.5, 70.6, 68.5, 62.4, 61.5, 55.7, 55.3, 40.9, 14.5. LC-MS (ESI+) calculated for C_18_H_26_NO_7_ [M + H]^+^ 368.1 found 368.

**Synthesis of compound 3**. Compound **1** (75 mg, 0.25 mmol, 1.0 eq.), propyl chloroformate (43 µL, 0.38 mmol, 1.5 eq.), and DIPEA (50 µL, 0.30 mmol, 1.2 eq.). The crude product was purified by column chromatography using 0–1% MeOH in DCM to afford a yellowish solid (70.0 mg, 0.18 mmol, 73%) as the desired product. R_f_ = 0.37 (3% MeOH/DCM), M.P. = 166.0–169.0 °C; ^1^H NMR (400 MHz, CDCl_3_) *δ* 7.31–7.19 (m, 5H), 5.10–4.99 (m, 1H), 4.76 (dd, 1H, *J* = 3.9, 6.3 Hz), 4.70–4.65 (m, 1H), 4.14–4.08 (dd, 1H, *J* = 4.9, 10.3 Hz), 4.05 (t, 2H, *J* = 6.8 Hz), 3.90–3.78 (m, 2H), 3.70–3.61 (m, 1H), 3.48 (dd~t, *J* = 10.3 Hz, 1H), 3.37 (s, 3H), 3.39–3.29 (m, 1H), 3.04 (dd, *J* = 14.2, 3.9 Hz, 1H), 2.94 (dd, *J* = 14.2, 6.3 Hz, 1H), 1.70–1.60 (m, 2H), 0.94 (t, *J* = 7.4 Hz, 3H); ^13^C NMR (100 MHz, CDCl_3_) *δ* 157.3, 136.1, 129.7, 128.3, 126.6, 102.8, 99.1, 81.5, 70.7, 68.5, 67.1, 62.4, 55.7, 55.3, 40.9, 22.3, 10.3. LC-MS (ESI+) calculated for C_19_H_28_NO_7_ [M + H]^+^ 382.1 found 382.

**Synthesis of compound 4**. Compound **1** (75 mg, 0.25 mmol, 1.0 eq.), allyl chloroformate (30 µL, 0.27 mmol, 1.1 eq.), and triethyl amine (35 µL, 0.25 mmol, 1.0 eq.). The crude product was purified by column chromatography using 0–1% MeOH in DCM to afford a yellowish solid (88.4 mg, 0.23 mmol, 93%) as the desired product. R_f_ = 0.47 (3% MeOH/DCM), M.P. = 174.0–175.0 °C; ^1^H NMR (400 MHz, CDCl_3_) *δ* 7.31–7.19 (m, 5H), 5.98–5.87 (m, 1H), 5.36–5.29 (m, 1H), 5.26–5.20 (m, 1H), 5.14–5.05 (br, 1H, -NH), 4.76 (dd, *J* = 6.2, 3.9 Hz, 1H), 4.68 (d, *J* = 3.2 Hz, 1H), 4.59 (d, *J* = 5.7 Hz, 2H), 4.11 (dd, *J* = 10.3, 4.9 Hz, 1H), 3.90–3.77 (m, 2H), 3.70–3.61 (m, 1H), 3.48 (dd~t, *J* = 10.3 Hz, 1H), 3.37 (s, 3H), 3.39–3.30 (m, 1H), 3.04 (dd, *J* = 14.2, 3.9 Hz, 1H), 2.93 (dd, *J* = 14.2, 6.3 Hz, 1H); ^13^C NMR (100 MHz, CDCl_3_) *δ* 156.7, 136.1, 132.5, 129.7, 128.3, 126.6, 118.1, 102.8, 99.1, 81.4, 70.8, 68.5, 66.2, 62.4, 55.7, 55.3, 40.9. LC-MS (ESI+) calculated for C_19_H_26_NO_7_ [M + H]^+^ 380.1 found 380.

**Synthesis of compound 5**. Compound **1** (75 mg, 0.25 mmol, 1.0 eq.), butyl chloroformate (30 µL, 0.27 mmol, 1.1 eq.), and triethyl amine (35 µL, 0.25 mmol, 1.0 eq.). The crude product was purified by column chromatography using 0–1% MeOH in DCM to afford a yellowish solid (86.1 mg, 0.22 mmol, 87%) as the desired product. R_f_ = 0.50 (3% MeOH/DCM) M.P. = 145.0–146.0 °C; ^1^H NMR (400 MHz, CDCl_3_) *δ* 7.31–7.19 (m, 5H), 5.06–4.98 (br, 1H, -NH), 4.76 (dd, *J* = 6.3, 3.9 Hz, 1H), 4.69–4.65 (m, 1H), 4.14–4.04 (m, 3H), 3.87–3.77 (m, 2H), 3.69–3.61 (m, 1H), 3.48 (dd~t, *J* = 10.3 Hz, 1H), 3.36 (s, 3H), 3.38–3.32 (m, 1H) 3.04 (dd, *J* = 14.2, 3.9 Hz, 1H), 2.93 (dd, *J* = 14.2, 6.3 Hz, 1H), 1.65–1.58 (m, 2H), 1.44–1.33 (m, 2H), 0.93 (t, *J* = 7.4 Hz, 3H); ^13^C NMR (100 MHz, CDCl_3_) *δ* 157.3, 136.1, 129.7, 128.3, 126.6, 102.8, 99.1, 81.5, 70.6, 68.5, 65.4, 62.4, 55.7, 55.3, 40.9, 31.0, 19.1, 13.7. LC-MS (ESI+) calculated for C_20_H_30_NO_7_ [M + H]^+^ 396.1 found 396.

**Synthesis of compound 6**. Compound **1** (75 mg, 0.25 mmol, 1.0 eq.), octyl chloroformate (75 µL,0.38 mmol, 1.5 eq.), and DIPEA (50 µL, 0.30 mmol, 1.2 eq.). The crude product was purified by column chromatography using 0–1% MeOH in DCM to afford a yellowish solid (91.3 mg, 0.20 mmol, 81%) as the desired product. R_f_ = 0.40 (3% MeOH/DCM) M.P. = 125.0–127.0 °C; ^1^H NMR (400 MHz, CDCl_3_) *δ* 7.31–7.19 (m, 5H), 5.08–4.98 (br, 1H, -NH), 4.76 (dd, *J* = 6.3, 3.9 Hz, 1H), 4.69–4.65 (m, 1H), 4.14–4.04 (m, 3H), 3.90–3.78 (m, 2H), 3.70–3.61 (m, 1H), 3.48 (dd~t, *J* = 10.3 Hz, 1H), 3.36 (s, 3H), 3.39–3.31 (m, 1H), 3.03 (dd, *J* = 14.2, 3.9 Hz, 1H), 2.93 (dd, *J* = 14.2, 6.3 Hz, 1H), 1.67–1.56 (m, 2H), 1.40–1.21 (m, 10H), 0.88 (t, *J* = 6.8 Hz, 3H); ^13^C NMR (100 MHz, CDCl_3_) *δ* 157.4, 136.1, 129.7, 128.3, 126.6, 102.8, 99.1, 81.5, 70.9, 68.5, 65.8, 62.4, 55.7, 55.3, 40.9, 31.8, 29.23, 29.17, 28.9, 25.8, 22.6, 14.1. LC-MS (ESI+) calculated for C_24_H_38_NO_7_ [M + H]^+^ 452.2 found 452.

**Synthesis of compound 7**. Compound **1** (75 mg, 0.25 mmol, 1.0 eq.), isopropyl chloroformate (63 µL, 0.30 mmol, 1.2 eq.), and DIPEA (50 µL, 0.30 mmol, 1.2 eq.). The crude product was purified by column chromatography using 0–2% MeOH in DCM to afford a yellowish solid (80 mg, 0.21 mmol, 84%) as the desired product. R_f_ = 0.43 (3% MeOH/DCM) M.P. = 155.0–158.0 °C; ^1^H NMR (400 MHz, CDCl_3_) *δ* 7.31–7.19 (m, 5H), 5.04–4.97 (m, 1H, -NH), 4.96–4.88 (m, 1H), 4.76 (dd, *J* = 4.0, 6.3 Hz, 1H), 4.69–4.65 (m, 1H), 4.10 (dd,, *J* = 4.9, 10.3 Hz, 1H), 3.87–3.78 (m, 2H), 3.69–3.61 (m, 1H), 3.48 (dd~t, *J* = 10.3 Hz, 1H), 3.36 (s, 3H), 3.38–3.30 (m, 1H), 3.04 (dd, *J* = 14.2, 3.9 Hz, 1H), 2.93 (dd, *J* =14.2, 6.3 Hz, 1H), 1.25 (dd, *J* = 6.3, 1.3 Hz, 6H); ^13^C NMR (100 MHz, CDCl_3_) *δ* 156.9, 136.1, 129.7, 128.3, 126.6, 102.8, 99.1, 81.5, 71.0, 69.0, 68.5, 62.3, 55.7, 55.3, 40.9, 22.09, 22.05. LC-MS (ESI+) calculated for C_19_H_28_NO_7_ [M + H]^+^ 382.1 found 382.

**Synthesis of compound 8**. Compound **1** (75 mg, 0.25 mmol, 1.0 eq.), isobutyl chloroformate (35 µL, 0.27 mmol, 1.1 eq.), and triethyl amine (35 µL, 0.25 mmol, 1.0 eq.). The crude product was purified by column chromatography using 0–1% MeOH in DCM to afford a yellowish solid (85.5 mg, 0.22 mmol, 87%) as the desired product. R_f_ = 0.50 (3% MeOH/DCM) M.P. = 149.0–151.0 °C; ^1^H NMR (400 MHz, CDCl_3_) *δ* 7.32–7.19 (m, 5H), 5.08–4.98 (br, 1H, -NH), 4.76 (dd, *J* = 6.3, 3.9 Hz, 1H), 4.71–4.66 (m, 1H), 4.11 (dd, *J* = 10.3, 4.9 Hz, 1H), 3.91–3.80 (m, 4H), 3.70–3.61 (m, 1H), 3.48 (dd~t, *J* = 10.3 Hz, 1H), 3.37 (s, 3H), 3.38–3.30 (m, 1H) 3.04 (dd, *J* = 14.2, 3.9 Hz, 1H), 2.93 (dd, *J* = 14.2, 6.3 Hz, 1H), 1.98–1.86 (m, 1H), 0.96 (d, *J* = 6.7 Hz, 6H); ^13^C NMR (100 MHz, CDCl_3_) *δ* 156.4, 135.1, 128.7, 127.3, 125.6, 101.8, 98.1, 80.5, 70.6, 69.7, 67.5, 61.4, 54.7, 54.3, 40.0, 27.0, 18.0. LC-MS (ESI+) calculated for C_20_H_30_NO_7_ [M + H]^+^ 396.1 found 396.

**Synthesis of compound 9**. Compound **1** (75 mg, 0.25 mmol, 1.0 eq.), trichloroethyl chloroformate (37 µL, 0.27 mmol, 1.1 eq.), and DIPEA (50 µL, 0.30 mmol, 1.2 eq.). The crude product was purified by column chromatography using 0–1% MeOH in DCM to afford a yellowish solid (78.9 mg, 0.17 mmol, 67%) as the desired product. R_f_ = 0.60 (3% MeOH/DCM) M.P. = 152.0–152.8 °C; ^1^H NMR (400 MHz, CDCl_3_) *δ* 7.31–7.20 (m, 5H), 5.34–5.26 (br, 1H, -NH), 4.80–4.68 (m, 4H), 4.14 (dd, *J* = 10.3, 4.9 Hz, 1H), 3.94–3.82 (m, 2H), 3.71–3.63 (m, 1H), 3.49 (dd~t, *J* = 10.3 Hz, 1H), 3.41 (s, 3H), 3.39–3.32 (m, 1H), 3.02 (dd, *J* = 14.2, 3.9 Hz, 1H), 2.94 (dd, *J* = 14.2, 6.3 Hz, 1H); ^13^C NMR (100 MHz, CDCl_3_) *δ* 155.0, 136.0, 129.6, 129.3, 126.7, 102.8, 98.9, 95.4, 81.3, 74.9, 70.5, 68.4, 62.4, 55.9, 55.4, 40.9. LC-MS (ESI+) calculated for C_18_H_23_Cl_3_NO_7_ [M + H]^+^ 470.0, 472.0 found 470 and 472 (due to 35 and 37 chlorine isotopes), and [M+Na]^+^ 492, 494. 

**Synthesis of compound 10**. Compound **1** (50 mg, 0.19 mmol, 1.0 eq.), phenyl chloroformate (23 µL, 0.18 mmol, 1.1 eq.), and triethyl amine (23 µL, 0.17 mmol, 1.0 eq.). The crude product was purified by column chromatography using 0–1% MeOH in DCM to afford a yellowish solid (65.3 mg, 0.14 mmol, 73%) as the desired product. R_f_ = 0.57 (3% MeOH/DCM) M.P. = 168.0–170.0 °C; ^1^H NMR (400 MHz, CDCl_3_) *δ* 7.38–7.11 (m, 10H), 5.46–5.36 (br, 1H, -NH), 4.82–4.72 (m, 2H), 4.13 (dd, 1H, *J* = 4.9, 10.3 Hz), 3.96–3.87 (m, 2H), 3.74–3.64 (m, 1H), 3.49 (dd~t, *J* = 10.3 Hz, 1H), 3.41 (s, 3H), 3.39–3.32 (m, 1H), 3.03 (dd, *J* = 14.2, 3.9 Hz, 1H), 2.93 (dd, *J* = 14.2, 6.3 Hz, 1H); ^13^C NMR (100 MHz, CDCl_3_) *δ* 155.1, 150.9, 136.1, 129.7, 129.3, 128.3, 126.6, 125.5, 121.5, 102.8, 98.9, 81.4, 70.6, 68.5, 62.4, 55.9, 55.4, 40.9. LC-MS (ESI+) calculated for C_22_H_26_NO_7_ [M + H]^+^ 416.1 found 416.

**Synthesis of compound 11**. Compound **1** (50 mg, 0.19 mmol, 1.0 eq.), benzyl chloroformate (24 µL, 0.18 mmol, 1.1 eq.), and DIPEA (26 µL, 0.17 mmol, 1.0 eq.). The crude product was purified by column chromatography using 0–1% MeOH in DCM to afford a yellowish solid (70.3 mg, 0.16 mmol, 86%) as the desired product. R_f_ = 0.50 (3% MeOH/DCM), M.P. = 156.0–157.0 °C; ^1^H NMR (400 MHz, CDCl_3_) *δ* 7.40–7.19 (m, 10H), 5.19–5.08 (br, 3H), 4.76 (dd, *J* = 6.3, 3.9 Hz, 1H), 4.72–4.63 (m, 1H), 4.11 (dd, *J* = 10.3, 4.9 Hz, 1H), 3.93–3.78 (m, 2H), 3.69–3.60 (m, 1H), 3.48 (dd~t, *J* = 10.3 Hz, 1H), 3.35 (s, 3H), 3.38–3.30 (m,1H), 3.03 (dd, *J* = 14.2, 4.0 Hz, 1H), 2.93 (dd, *J* = 14.2, 6.3 Hz, 1H); ^13^C NMR (100 MHz, CDCl_3_) *δ* 156.9, 136.1, 129.7, 128.6, 128.3, 126.6, 102.8, 99.0, 81.4, 70.8, 68.5, 67.4, 62.4, 55.8, 55.3, 40.9. LC-MS (ESI+) calculated for C_23_H_28_NO_7_ [M + H]^+^ 430.1 found 430. 

**Synthesis of compound 12**. Compound **1** (75 mg, 0.25 mmol, 1.0 eq.), 4-nitrobenzyl chloroformate (64.7 mg, 0.30 mmol, 1.2 eq.), and DIPEA (50 µL, 0.30 mmol, 1.2 eq.). The crude product was purified by column chromatography using 0–1% MeOH in DCM to afford a yellowish solid (113.0 mg, 0.24 mmol, 95%) as the desired product. R_f_ = 0.40 (3% MeOH/DCM) M.P. = 212.0–214.0 °C; ^1^H NMR (400 MHz, CDCl_3_) *δ* 8.21 (d, *J* = 8.7 Hz, 2H), 7.52 (d, *J* = 8.7 Hz, 2H), 7.31–7.20 (m, 5H), 5.26–5.14 (br, 3H), 4.76 (dd, *J* = 6.3, 3.9 Hz, 1H), 4.72–4.66 (m, 1H), 4.11 (dd, 1H, *J* = 4.9, 10.3 Hz), 3.94–3.78 (m, 2H), 3.71–3.61 (m, 1H), 3.48 (dd~t, *J* = 10.3 Hz, 1H), 3.37 (s, 3H), 3.36–3.28 (m, 1H), 3.03 (dd, *J* = 14.2, 4.2 Hz, 1H), 2.93 (dd, *J* = 14.2, 6.3 Hz, 1H); ^13^C NMR (100 MHz, CDCl_3_) *δ* 156.3, 147.7, 143.5, 136.0, 129.6, 128.3, 128.2, 126.7, 123.8, 102.8, 98.9, 81.4, 70.7, 68.4, 65.7, 62.4, 55.8, 55.3, 40.9. LC-MS (ESI+) calculated for C_23_H_27_N_2_O_9_ [M + H]^+^ 475.1 found 475.

**Synthesis of compound 13**. Compound **1** (75 mg, 0.25 mmol, 1.0 eq.), 4-chlorophenyl chloroformate (39 µL, 0.27 mmol, 1.1 eq.), and triethyl amine (35 µL, 0.25 mmol, 1.0 eq.). The crude product was purified by column chromatography using 0–3% MeOH in DCM to afford a yellowish solid (110.5 mg, 0.24 mmol, 98%) as the desired product. R_f_ = 0.65 (5% MeOH/DCM) M.P. = 170.0–171.0 °C; ^1^H NMR (400 MHz, CDCl_3_) *δ* 7.33–7.20 (m, 6H), 7.12 (d, J = 8.8 Hz, 2H), 5.44–5.33 (m, 1H, -NH), 4.82–4.71 (m, 2H), 4.13 (dd, 1H, *J* = 4.9, 10.3 Hz), 3.98–3.84 (m, 2H), 3.74–3.63 (m, 1H), 3.0 (dd~t, *J* = 10.3 Hz, 1H), 3.41 (s, 3H), 3.38–3.31 (m, 1H), 3.03 (dd, *J* = 14.1, 4.1 Hz, 1H), 2.94 (dd, *J* = 14.1, 6.0 Hz, 1H); ^13^C NMR (100 MHz, CDCl_3_) *δ* 154.6, 149.4, 136.0, 130.8, 129.6, 129.3, 128.3, 126.7, 122.8, 102.8, 98.9, 81.3, 70.5, 68.4, 62.4, 55.9, 55.4, 40.9. LC-MS (ESI+) calculated for C_22_H_25_ClNO_7_ [M + H]^+^ 450.1 found 450.

**Synthesis of compound 14**. Compound **1** (75 mg, 0.25 mmol, 1.0 eq.), Fmoc chloroformate (52 mg, 0.20 mmol, 1.2 eq.), and DIPEA (35 µL, 0.20 mmol, 1.2 eq.). The crude product was purified by column chromatography using 0–1% MeOH in DCM to afford a yellowish solid (51.8 mg, 0.10 mmol, 59%) as the desired product. R_f_ = 0.50 (3% MeOH/DCM) M.P. = 168.0–170.0 °C; ^1^H NMR (400 MHz, CDCl_3_) *δ* 7.79–7.74 (m, 2H), 7.62–7.57 (m, 2H), 7.44–7.37 (m, 2H), 7.34–7.20 (m, 7H), 5.14–5.03 (br, 1H), 4.76 (dd, *J* = 6.3, 3.9 Hz, 1H), 4.70–4.63 (m, 1H), 4.53–4.38 (m, 2H), 4.26–4.20 (m, 1H), 4.11 (dd, 1H, *J* = 4.9, 10.3 Hz), 3.92–3.79 (m, 2H), 3.70–3.61 (m, 1H), 3.48 (dd~t, *J* = 10.3 Hz, 1H), 3.37 (s, 3H), 3.34–3.30 (m, 1H), 3.04 (dd, *J* = 14.1, 4.0 Hz, 1H), 2.94 (dd, *J* = 14.3, 6.2 Hz, 1H); ^13^C NMR (100 MHz, CDCl_3_) *δ* 156.9, 143.8, 141.4, 136.1, 129.7, 128.3, 127.8, 127.1, 126.6, 125.1, 125.0, 120.0, 102.8, 99.0, 81.4, 70.6, 68.5, 67.1, 62.4, 55.8, 55.3, 47.3, 40.9, 29.7. LC-MS (ESI+) calculated for C_30_H_32_NO_7_ [M + H]^+^ 518.2 found 518.

## Figures and Tables

**Table 1:** Gelation performance carbamates in several solvents.

	R:	Toluene	Pump Oil	Glycerol	Et-Glycol	EtOH:H_2_O (1:1)	EtOH:H_2_O (1:2)	DMSO:H_2_O (1:1)	DMSO:H_2_O (1:2)	H_2_O
**2**	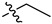	P	UG 20.0_O_	G 6.7_T_	UG 20.0_T_	G 20.0_T_	G 20.0_T_	S	G 6.7_T_	U G 6.7_O_
**3**	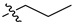	P	G 4.0_O_	G 6.7_T_	G 20.0_O_	G 10.0_O_	P	G 20.0_O_	G 10.0_O_	I
**4**	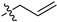	G 20.0_O_	G 3.3_O_	G 5.0_O_	P	P	P	G 20.0_O_	G 6.7_O_	I
**5**	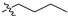	P	G 6.7_O_	G 4.0_O_	G 20.0_O_	G 10.0_O_	G 6.7_O_	G 20.0_O_	G 4.0_O_	P
**6**	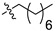	S	G 6.7_O_	G 6.7_T_	G 4.0_O_	G 2.8_O_	P	G 2.8_O_	G 6.7_O_	P
**7**	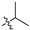	S	S	G 5.0_C_	G 20.0_C_	G 20.0_O_	G 2.8_T_	G 6.7_T_	G 3.3_C_	G 2.0_T_
**8**	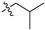	S	G 6.7_T_	G 3.3_C_	P	S	G 1.7 _O_	G 4.0_T_	G 4.0_O_	G 10.0_O_
**9**	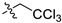	S	S	G 6.7_T_	S	G 10.0_O_	P	P	I	I
**10**	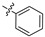	P	UG 6.7_T_	G10.0_C_	S	P	P	P	P	I
**11**	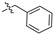	S	G 3.6_T_	G 1.3_T_	G 5.0_C_	G 10.0_T_	G 3.3_O_	G 6.7_T_	G 6.7_O_	G 4.0_O_
**12**	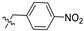	S	G 20.0_O_	G 6.7_T_	G 10.0_C_	G 3.3_O_	G 2.8_O_	G 2.5_O_	G 1.8_O_	I
**13**	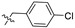	S	S	G 6.7_C_	G 10.0_T_	P	S	G 20.0_T_	P	I
**14**	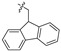	P	G 4.0_O_	G 6.7_T_	G 6.7_T_	UG 6.7_O_	UG 6.7_O_	G 4.0_O_	G 10.0_T_	I

All compounds were tested starting from 20.0 mg/mL. UG, unstable gel at room temperature and the numbers are concentrations in mg/mL; G, stable gel at room temperature and the numbers are MGC in mg/mL; P, precipitation; S, soluble; and I, insoluble. Gel appearance: C for clear or transparent; T, translucent; and O, opaque. Compounds **5** and **6** formed opaque gels in hexane at 5.0 mg/mL, while **4** and **8** formed precipitate, and all others were insoluble in hexane. The ratios for all mixed solvents are volume ratios.

**Table 2:** Comparison of gelation properties of the two series of carbamate derivatives.

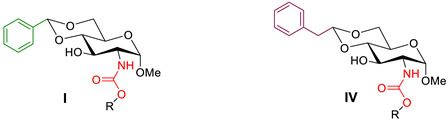								
R	**I**	EtOH:H_2_O (1:2)	DMSO:H_2_O (1:2)	H_2_O	**IV**	EtOH:H_2_O (1:2)	DMSO: H_2_O (1:2)	H_2_O
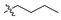	**I-a**	G 10.0	G 10.0	I	**5, IV-a**	G 6.7	G 4.0	P
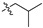	**I-b**	G 2.2	G 1.7	G 4.0	**8, IV-b**	G 1.7	G 4.0	G 10.0
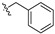	**I-c**	G 10.0	G 20.0	I	**11, IV-c**	G 3.3	G 6.7	G 4.0

**Table 3:** Gelation test results of metallogels of compound **8** with different metal salts.

Metal Salt (1.5 Eq.)	Concentration of Compound 8
Cu(OAc)_2_⋅H_2_O	G 3.0_O_
Cu(SO_4_)⋅5H_2_O	G 3.0_O_
CuBr_2_	G 3.0_O_
Hg(OAc)_2_	P
Zn(OAc)_2_⋅2H_2_O	G 2.5_O_
NiCl_2_⋅6H_2_O	G 2.5_O_
Pb(OAc)_4_	G 3.8_O_
FeCl_2_	G 3.0_O_
FeCl_3_⋅6H_2_O	G 3.0_O_
AgNO_3_	G 3.8_O_

## Data Availability

Not applicable.

## Supplementary Materials

The following supporting information can be downloaded at: https://www.mdpi.com/article/10.3390/gels8030191/s1, Part I: Copies of NMR (^1^H and ^13^C) spectra of carbamate derivatives **2–14**; and 2D spectra for compounds **3**, **6**, and **10** and Part II: NMR spectra of compound **8** at variable temperatures; procedures for gelation tests and water tolerance studies; details for metallogels’ formation and analysis; rheological analysis data; chemiluminescence analysis, drug loading, and release studies; conductivity measurement for co-gels with TBA salts; and LCMS data. Figure S1. The ^1^H NMR spectra of compound **8** from 30–60 °C at 8.0 mg/mL in DMSO-d_6_; Figure S2. Gels of compound **8** in 0.1 mL of DMSO and incremental amounts of water; Figure S3a. Gels formed by compound **8** with 1.5 eq. of metal salt at 5.0 mg/mL; Figure S3b. Gels formed by compound **11** with 1.0 eq. of metal salt at 3.0 mg/mL; Figure S3c. Overlay of IR spectra of compound **8**; Figure S4a–g, Amplitude sweep data graphs for several gels; Figure S5. Chemiluminescence intensities of luminol with Metal-Organic-Xerogels of compound **8**; Figure S6. The gel images of compound **11** and naproxen in 2.0 mL of DMSO:H_2_O (*v*/*v* 1:10); Figure S7. Conductivity correspondence to concentrations of TBABr solutions; Figure S8. Conductivities of the co-gels formed by compound **7** in the presence of TBABr and the TBABr solutions. Table S1. Gelation properties of compound **8** in different amount of water; Table S2. The gelation concentrations of compound **8** after adding water to metallogels; Table S3. G′/G″ ratios for several gels; Table S4a. G′/G″ ratios for various gels and metallogels; Table S4b. G′/G″ ratios for metallogels; Table S5a. Chemiluminescence data for compound **8**; Table S5b. Chemiluminescence data for compound **11**; Table S6. Conductivity data of multicomponent gels.

## References

[1] Sangeetha N.M., Maitra U. (2005). Supramolecular gels: Functions and uses. Chem. Soc. Rev..

[2] Basu N., Chakraborty A., Ghosh R. (2018). Carbohydrate derived organogelators and the corresponding functional gels developed in recent time. Gels.

[3] Morris J., Bietsch J., Bashaw K., Wang G. (2021). Recently Developed Carbohydrate Based Gelators and Their Applications. Gels.

[4] Du X.Z.J., Shi J., Xu B. (2015). Supramolecular Hydrogelators and Hydrogels: From Soft Matter to Molecular Biomaterials. Chem. Rev..

[5] Lim J.Y.C., Goh S.S., Liow S.S., Xue K., Loh X.J. (2019). Molecular gel sorbent materials for environmental remediation and wastewater treatment. J. Mater. Chem. A.

[6] Narayana C., Upadhyay R.K., Chaturvedi R., Sagar R. (2017). A versatile carbohydrate based gelator for oil water separation, nanoparticle synthesis and dye removal. New J. Chem..

[7] Okesola B.O., Smith D.K. (2016). Applying low-molecular weight supramolecular gelators in an environmental setting-self-assembled gels as smart materials for pollutant removal. Chem. Soc. Rev..

[8] Latxague L., Ramin M.A., Appavoo A., Berto P., Maisani M., Ehret C., Chassande O., Barthelemy P. (2015). Control of Stem-Cell Behavior by Fine Tuning the Supramolecular Assemblies of Low-Molecular-Weight Gelators. Angew. Chem. Int. Ed..

[9] Fang W., Zhang Y., Wu J., Liu C., Zhu H., Tu T. (2018). Recent Advances in Supramolecular Gels and Catalysis. Chem. Asian J..

[10] Draper E.R., Adams D.J. (2017). Low-Molecular-Weight Gels: The State of the Art. Chem.

[11] Mayr J., Saldias C., Diaz Diaz D. (2018). Release of small bioactive molecules from physical gels. Chem. Soc. Rev..

[12] Tam A.Y.-Y., Yam V.W.-W. (2013). Recent advances in metallogels. Chem. Soc. Rev..

[13] Goyal N., Cheuk S., Wang G. (2010). Synthesis and characterization of d-glucosamine-derived low molecular weight gelators. Tetrahedron.

[14] Chen A., Adhikari S.B., Mays K., Wang G. (2017). Synthesis and Study of Molecular Assemblies Formed by 4,6-O-(2-Phenylethylidene)-Functionalized D-Glucosamine Derivatives. Langmuir.

[15] Wang G., Cheuk S., Yang H., Goyal N., Reddy P.V.N., Hopkinson B. (2009). Synthesis and Characterization of Monosaccharide-Derived Carbamates as Low-Molecular-Weight Gelators. Langmuir.

[16] Bietsch J., Olson M., Wang G. (2021). Fine-Tuning of Molecular Structures to Generate Carbohydrate Based Super Gelators and Their Applications for Drug Delivery and Dye Absorption. Gels.

[17] Wang D., Chen A., Morris J., Wang G. (2020). Stimuli-responsive gelators from carbamoyl sugar derivatives and their responses to metal ions and tetrabutylammonium salts. RSC Adv..

[18] Christoff-Tempesta T., Lew A.J., Ortony J.H. (2018). Beyond covalent crosslinks: Applications of supramolecular gels. Gels.

[19] Slavik P., Kurka D.W., Smith D.K. (2018). Palladium-scavenging self-assembled hybrid hydrogels-reusable highly-active green catalysts for Suzuki-Miyaura cross-coupling reactions. Chem. Sci..

[20] Piepenbrock M.O., Lloyd G.O., Clarke N., Steed J.W. (2010). Metal- and anion-binding supramolecular gels. Chem. Rev..

[21] Li L., Sun R., Zheng R., Huang Y. (2021). Anions-responsive supramolecular gels: A review. Mater. Des..

[22] Bielejewski M., Nowicka K., Bielejewska N., Tritt-Goc J. (2016). Ionic conductivity and thermal properties of a supramolecular ionogel made from a sugar-based low molecular weight gelator and a quaternary ammonium salt electrolyte solution. J. Electrochem. Soc..

[23] Guo P., Su A., Wei Y., Liu X., Li Y., Guo F., Li J., Hu Z., Sun J. (2019). Healable, Highly Conductive, Flexible, and Nonflammable Supramolecular Ionogel Electrolytes for Lithium-Ion Batteries. ACS Appl. Mater. Interfaces.

[24] Zheng Y., Li G., Zhang Y. (2016). Organometallic Hydrogels. ChemNanoMat.

[25] Wu H., Zheng J., Kjoniksen A.L., Wang W., Zhang Y., Ma J. (2019). Metallogels: Availability, Applicability, and Advanceability. Adv. Mater..

[26] Dastidar P., Ganguly S., Sarkar K. (2016). Metallogels from Coordination Complexes, Organometallic, and Coordination Polymers. Chem.-Asian J..

[27] Karan C.K., Sau M.C., Bhattacharjee M. (2017). A copper(II) metal-organic hydrogel as a multifunctional precatalyst for CuAAC reactions and chemical fixation of CO_2_ under solvent free conditions. Chem. Commun..

[28] Anh H.T.P., Huang C.-M., Huang C.-J. (2019). Intelligent Metal-Phenolic Metallogels as Dressings for Infected Wounds. Sci. Rep..

[29] Wang A., Shi W., Huang J., Yan Y. (2016). Adaptive soft molecular self-assemblies. Soft Matter.

[30] Karan C.K., Bhattacharjee M. (2019). A Copper Metal-Organic Hydrogel as a Catalyst for SO2 and CO2 Fixation under Ambient Conditions. Eur. J. Inorg. Chem..

[31] Li B., Xiao D., Gai X., Yan B., Ye H., Tang L., Zhou Q. (2021). A multi-responsive organogel and colloid based on the self-assembly of a Ag(I)-azopyridine coordination polymer. Soft Matter.

[32] Jiang Z.W., Zhao T.T., Li Y.F., Huang C.Z. (2020). Dimension conversion: From a 1D metal-organic gel into a 3D metal-organic porous network with high-efficiency multiple enzyme-like activities for cascade reactions. Nanoscale Horiz..

[33] Guo M.X., Li Y.F. (2019). Cu (II)-based metal-organic xerogels as a novel nanozyme for colorimetric detection of dopamine. Spectrochim. Acta Part A.

[34] Zhao T.T., Jiang Z.W., Zhen S.J., Huang C.Z., Li Y.F. (2019). A copper(II)/cobalt(II) organic gel with enhanced peroxidase-like activity for fluorometric determination of hydrogen peroxide and glucose. Microchim. Acta.

[35] Bailey T.S., Pluth M.D. (2013). Chemiluminescent Detection of Enzymatically Produced Hydrogen Sulfide: Substrate Hydrogen Bonding Influences Selectivity for H2S over Biological Thiols. J. Am. Chem. Soc..

[36] He L., Peng Z.W., Jiang Z.W., Tang X.Q., Huang C.Z., Li Y.F. (2017). Novel Iron(III)-Based Metal-Organic Gels with Superior Catalytic Performance toward Luminol Chemiluminescence. ACS Appl. Mater. Interfaces.

[37] Zhang L., Hou Y., Lv C., Liu W., Zhang Z., Peng X. (2020). Copper-based metal-organic xerogels on paper for chemiluminescence detection of dopamine. Anal. Methods.

[38] Sun X., Lei J., Jin Y., Li B. (2020). Long-Lasting and Intense Chemiluminescence of Luminol Triggered by Oxidized g-C3N4 Nanosheets. Anal. Chem..

[39] Ye J., Zhu L., Yan M., Xiao T., Fan L., Xue Y., Huang J., Yang X. (2021). An intensive and glow-type chemiluminescence of luminol-embedded, guanosine-derived hydrogel. Talanta.

[40] Yang C.P., He L., Huang C.Z., Li Y.F., Zhen S.J. (2021). Continuous singlet oxygen generation for persistent chemiluminescence in Cu-MOFs-based catalytic system. Talanta.

[41] Zong L.-P., Ruan L.-Y., Li J., Marks R.S., Wang J.-S., Cosnier S., Zhang X.-J., Shan D. (2021). Fe-MOGs-based enzyme mimetic and its mediated electrochemiluminescence for in situ detection of H2O2 released from Hela cells. Biosens. Bioelectron..

[42] Yu J., Cao M., Wang H., Li Y. (2019). Novel manganese(II)-based metal-organic gels: Synthesis, characterization and application to chemiluminescent sensing of hydrogen peroxide and glucose. Microchim. Acta.

[43] Tang X.Q., Xiao B.W., Li C.M., Wang D.M., Huang C.Z., Li Y.F. (2017). Co-metal-organic-frameworks with pure uniform crystal morphology prepared via Co_2_ + exchange-mediated transformation from Zn-metallogels for luminol catalysed chemiluminescence. Spectrochim. Acta Part A.

[44] Tao L., Huo Z.P., Ding Y., Wang L., Zhu J., Zhang C.N., Pan X., Nazeeruddin M.K., Dai S.Y., Gratzel M. (2014). Gel electrolyte materials formed from a series of novel low molecular mass organogelators for stable quasi-solid-state dye-sensitized solar cells. J. Mater. Chem. A.

[45] Bielejewski M., Lapinski A., Demchuk O. (2017). Molecular interactions in high conductive gel electrolytes based on low molecular weight gelator. J. Colloid Interface Sci..

[46] Sharma P., Chen A., Wang D., Wang G. (2021). Synthesis and Self-Assembling Properties of Peracetylated β-1-Triazolyl Alkyl D-Glucosides and D-Galactosides. Chemistry.

