# Supplementary material for: 4,6-O-Phenylethylidene Acetal Protected D-Glucosamine Carbamate-Based Gelators and Their Applications for Multi-Component Gels
Source: Gels. 2022 Mar 19;8(3):191. doi: 10.3390/gels8030191 (PMC8953293; doi:10.3390/gels8030191)

Supplemental Information

4, 6-*O*-Phenylethylidene Acetal Protected D-Glucosamine Carbamate-based Gelators and Their Applications for Multi-component Gels

Pooja Sharma and Guijun Wang*

Department of Chemistry and Biochemistry, Old Dominion University, Norfolk, VA 23529, g1wang@odu.edu

Table of contents

Part I.

^{1}H and ^{13}C NMR spectra of compounds **2-14**…………..…………………………………..……...S2

2D-NMR spectra of compounds **3**, **6** and **10**….…………………………………………………...S16

Part II.

1.^{1}H NMR spectra for compound **8** at variable temperatures…………………………………......S22

2. Procedure for gelation tests and water dilution study……………………………….……….S23

3. Metallogel formation and characterizations..……………………………………….……….S25

4. Rheological analysis data...………………..…………………………...……………..S28

5. Chemiluminescence analysis …………………………………..……………………….…..S34

6. Drug loading and release studies... …………………………………………...…………..S36

7. Conductivity measurement of co-gels…..…………………………………………………..…S38

8. LCMS data……………………………………..……………………………………………S41

Part I. ^{1}H and ^{13}C NMR spectra of compounds 2-14

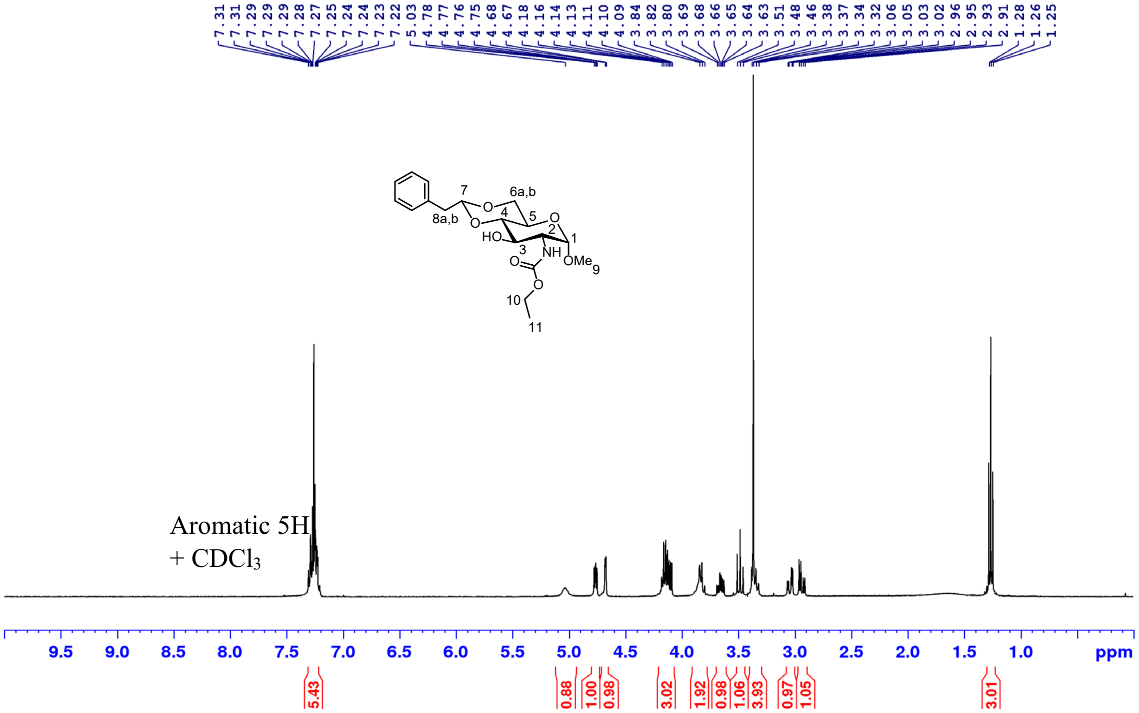

^{1}H NMR spectrum for compound **2** in $CDCl_3$

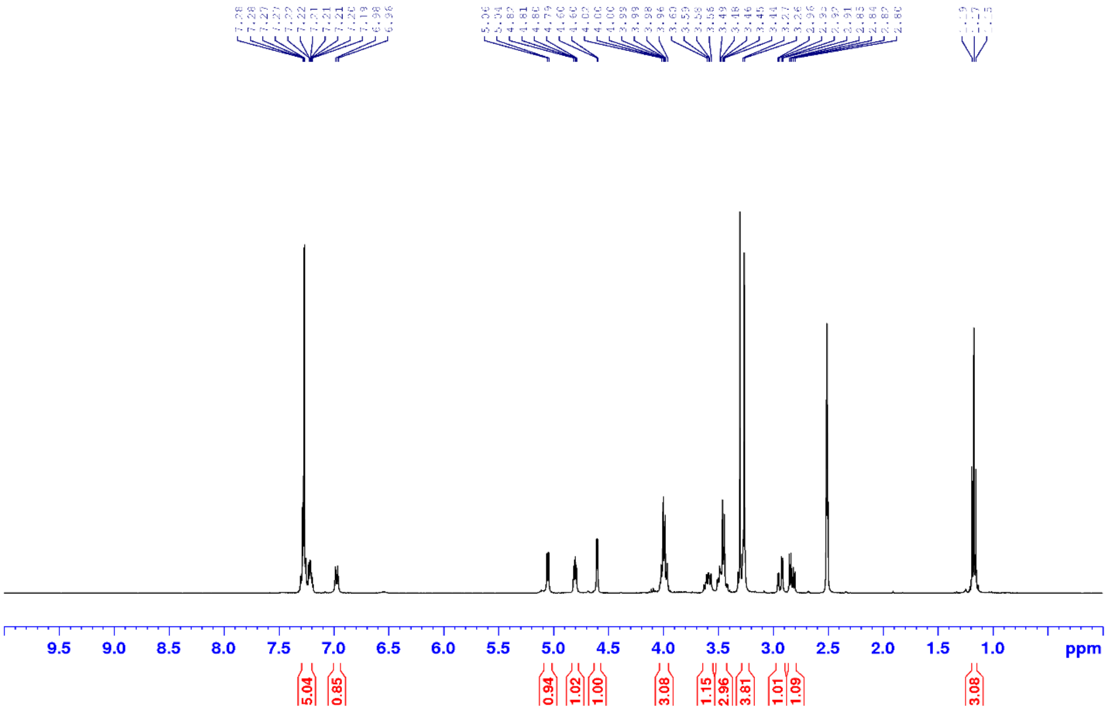

^{1}H NMR spectrum for compound **2** in DMSO-d_6

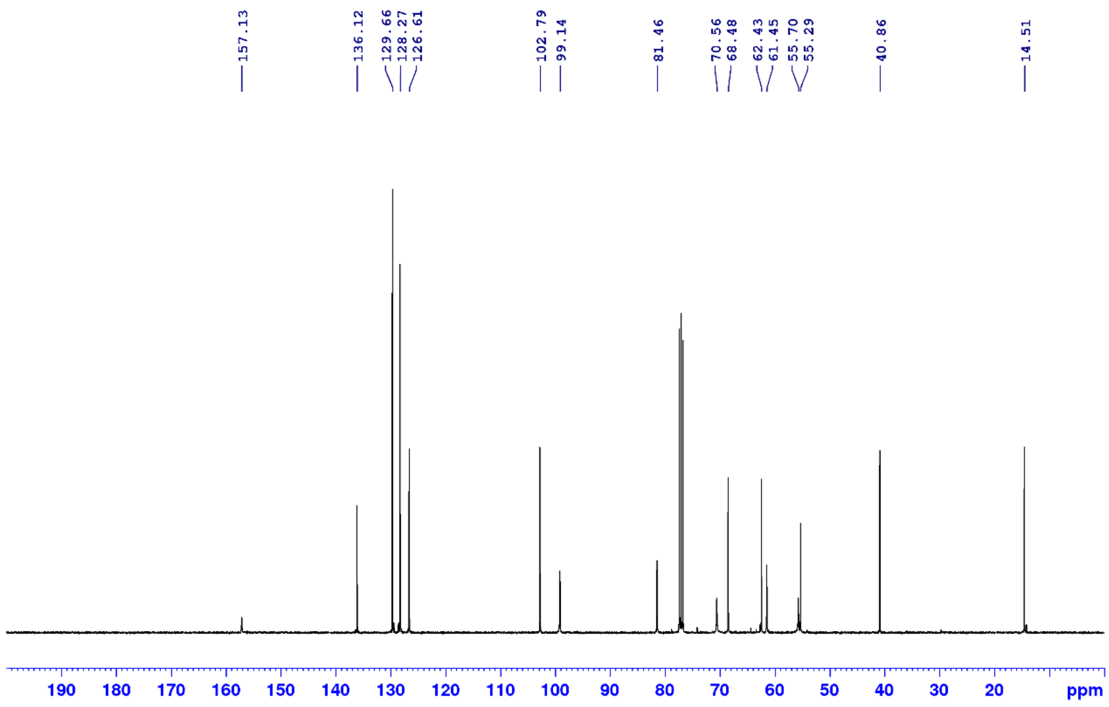

^{13}C NMR spectrum for compound **2** in $CDCl_3$

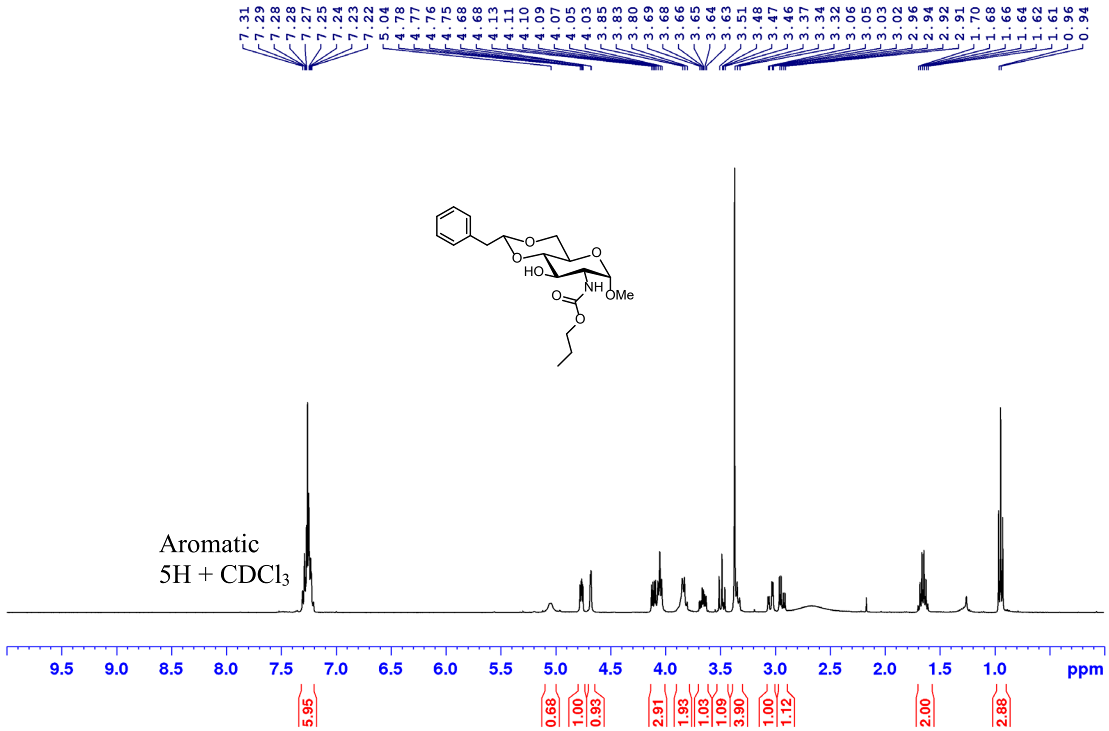

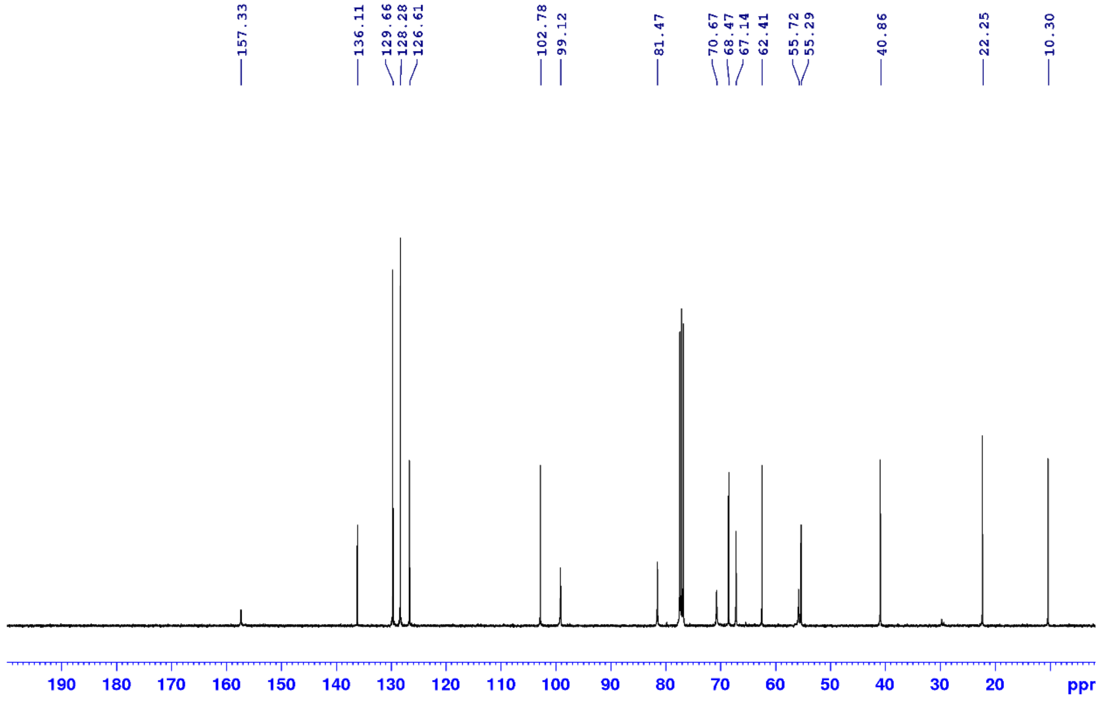

^{1}H NMR and ^{13}C NMR spectra for compound **3** in $CDCl_3$

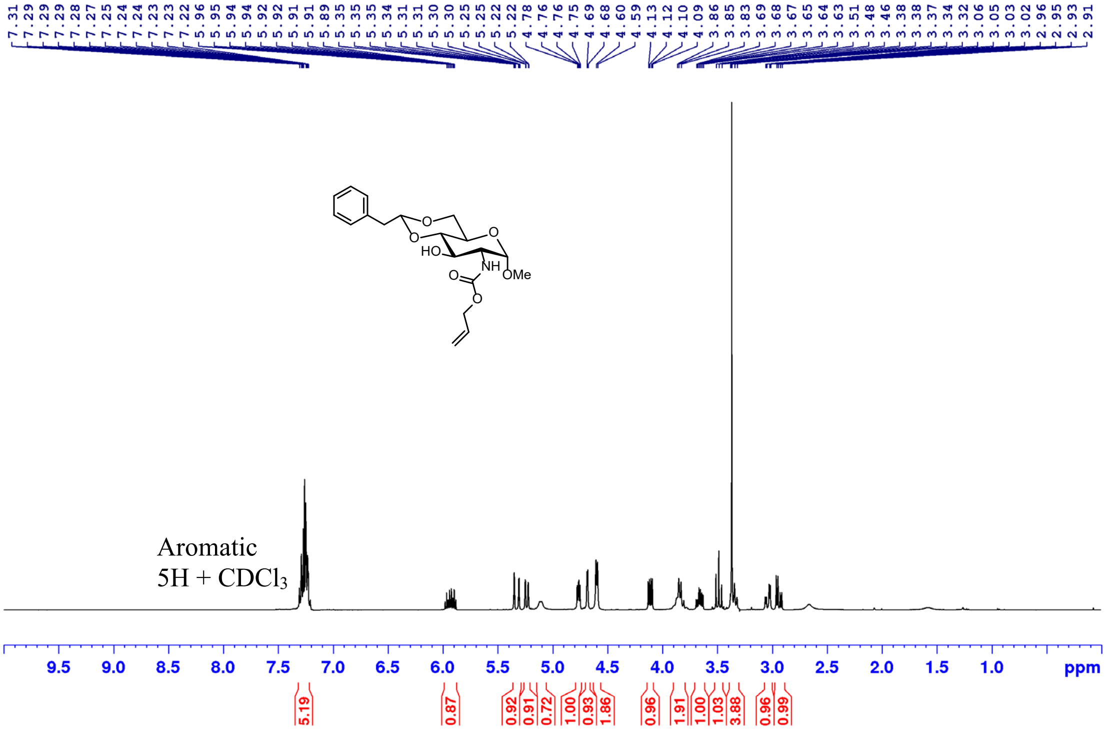

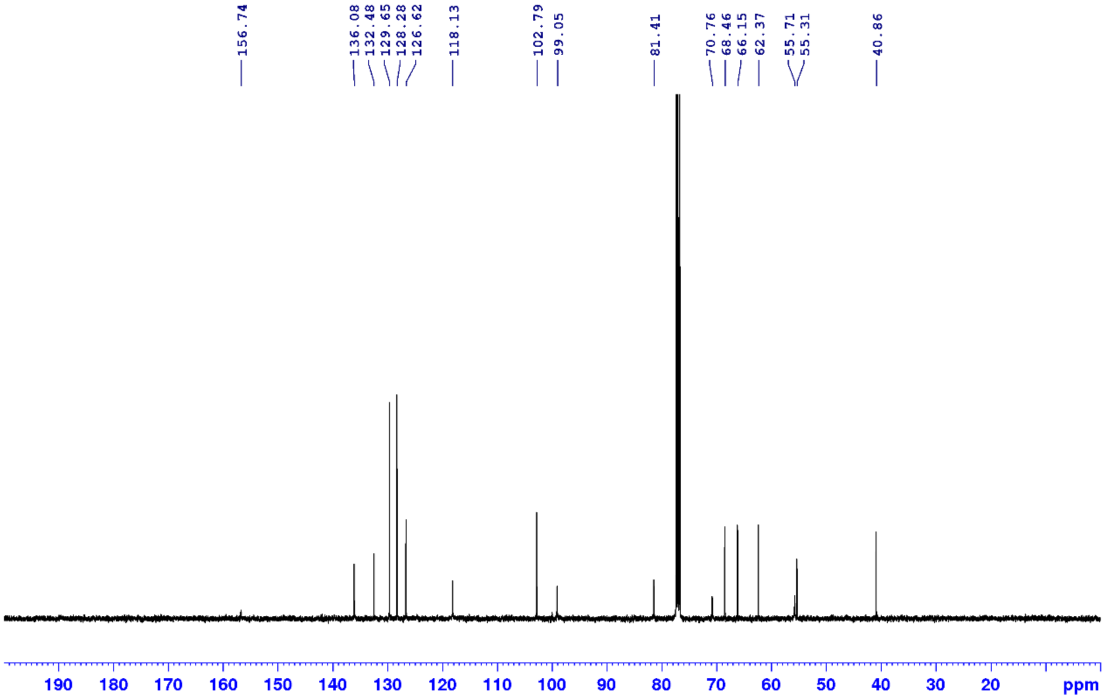

^{1}H NMR and ^{13}C NMR spectra for compound **4** in $CDCl_3$

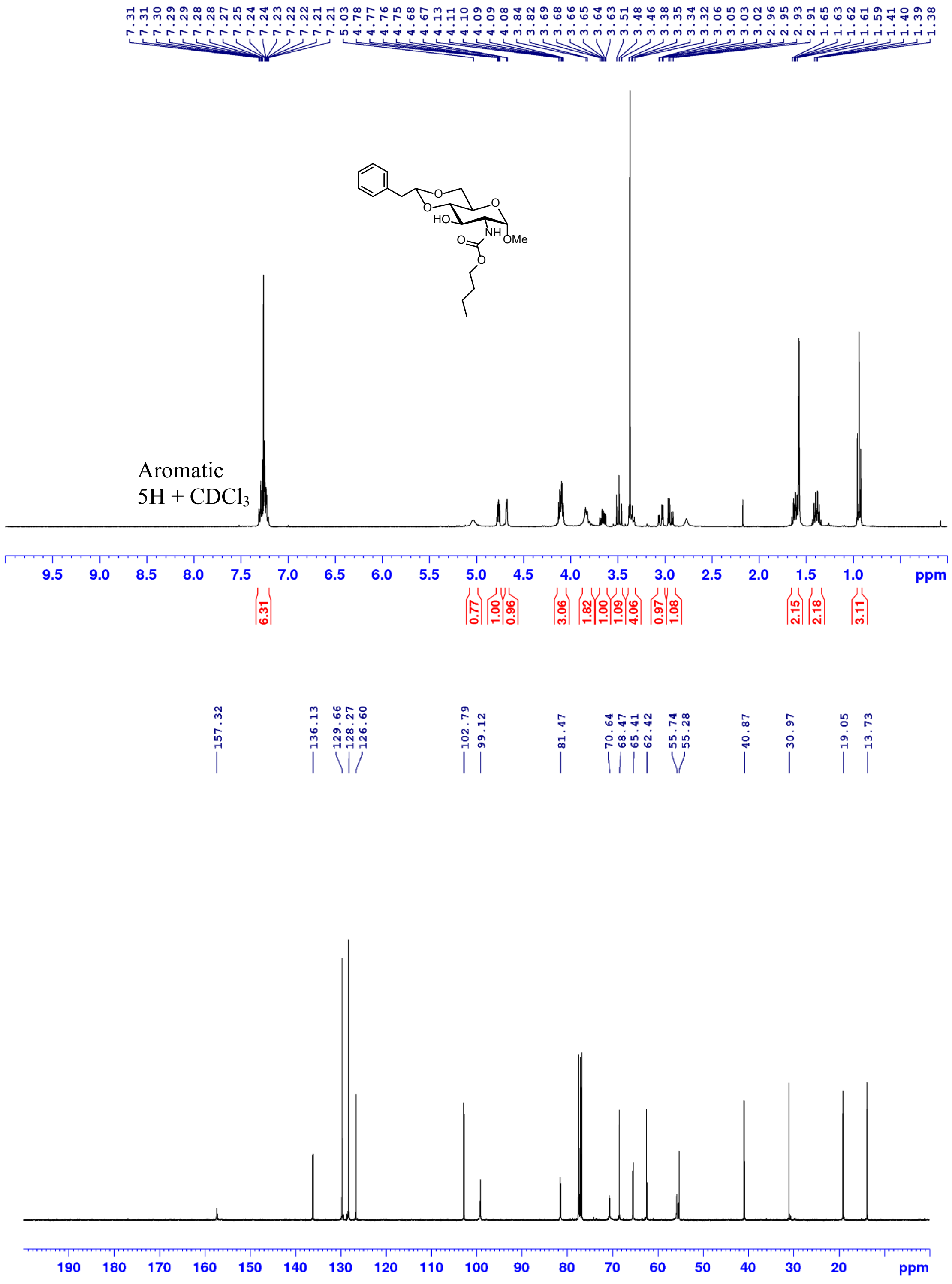

^{1}H NMR and ^{13}C NMR spectra for compound **5** in $CDCl_3$

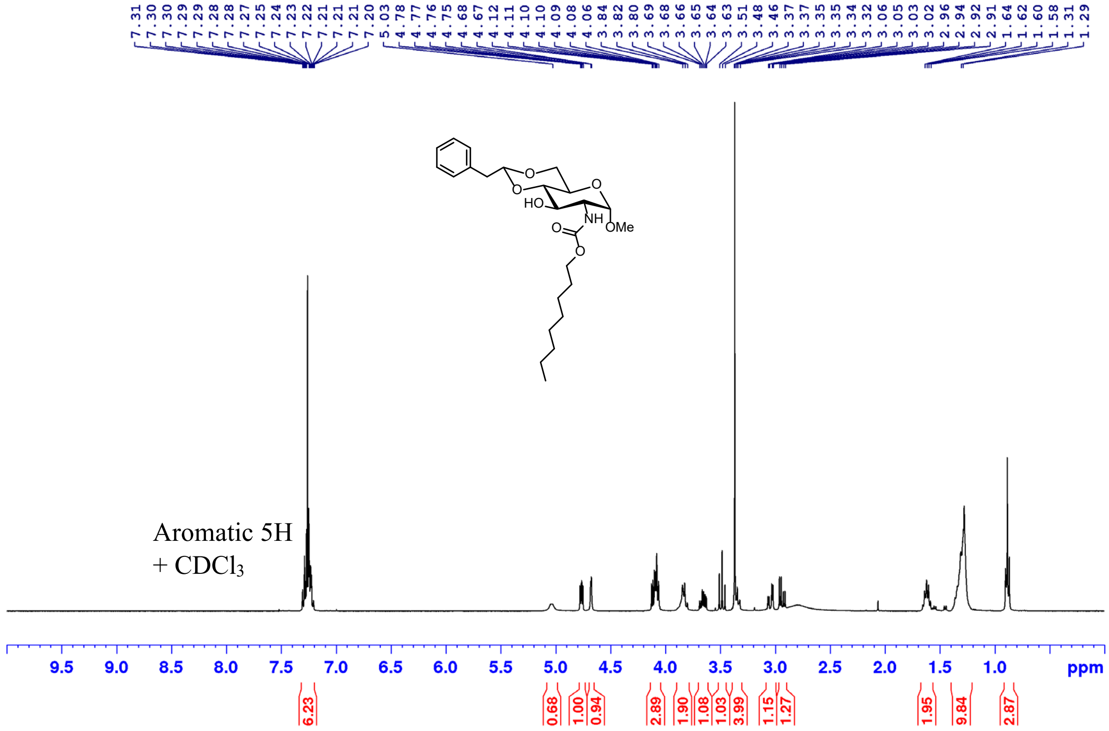

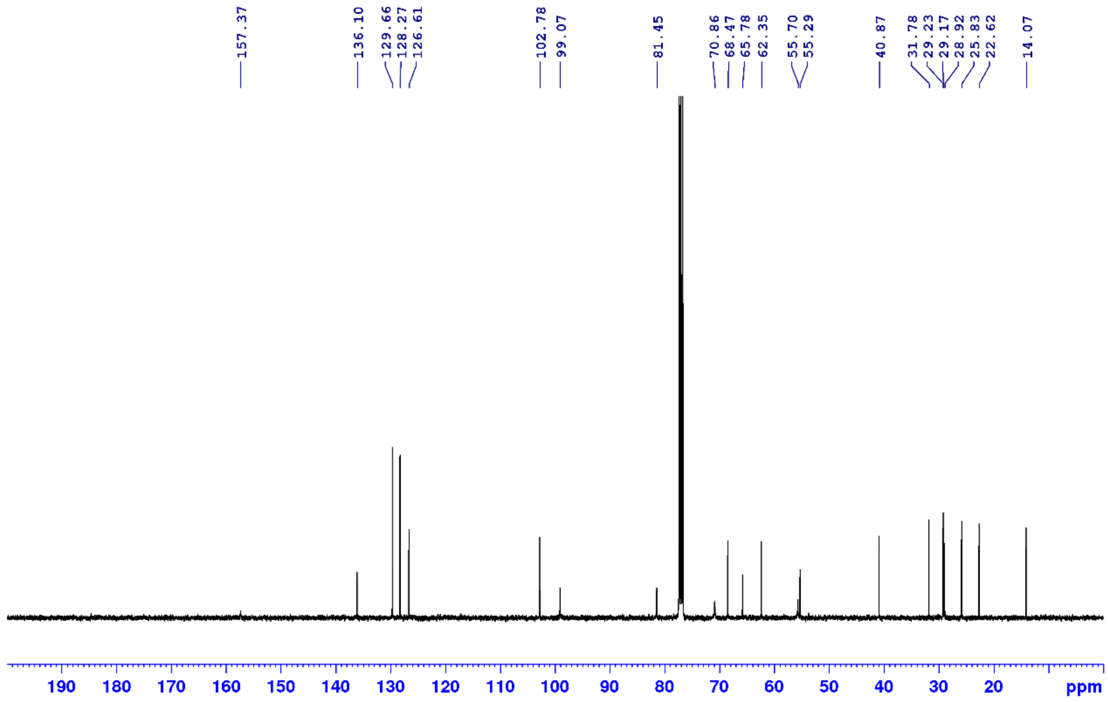

^{1}H NMR and ^{13}C NMR spectra for compound **6** in $CDCl_3$

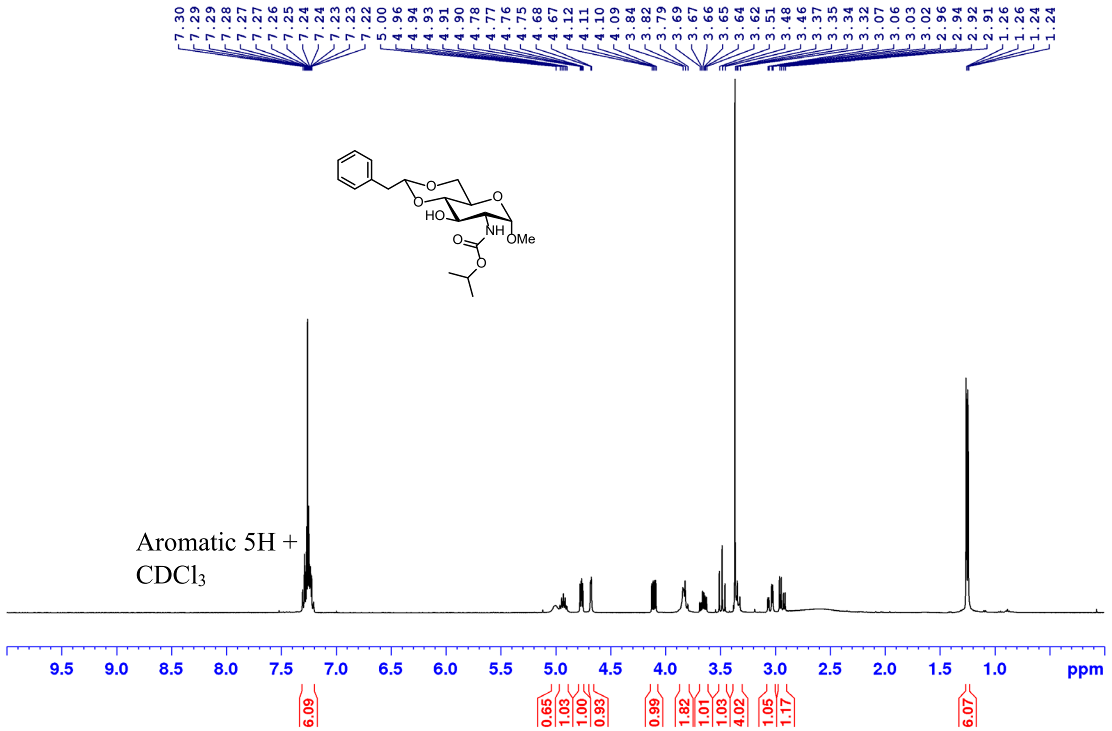

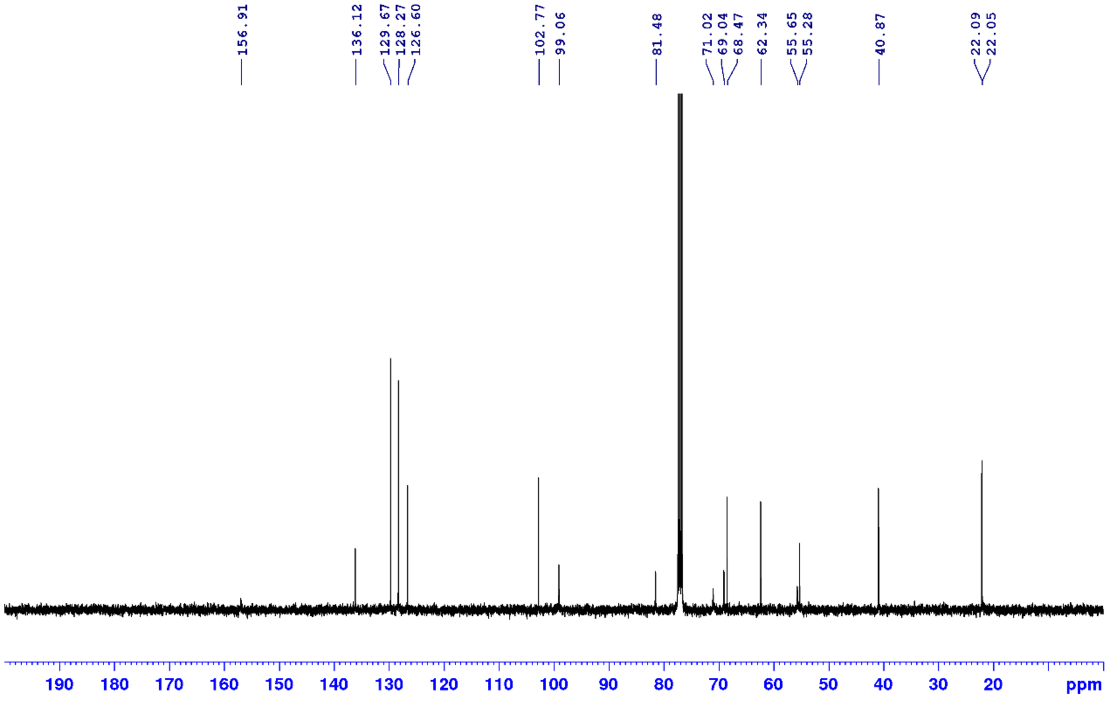

^{1}H NMR and ^{13}C NMR spectra for compound **7** in $CDCl_3$

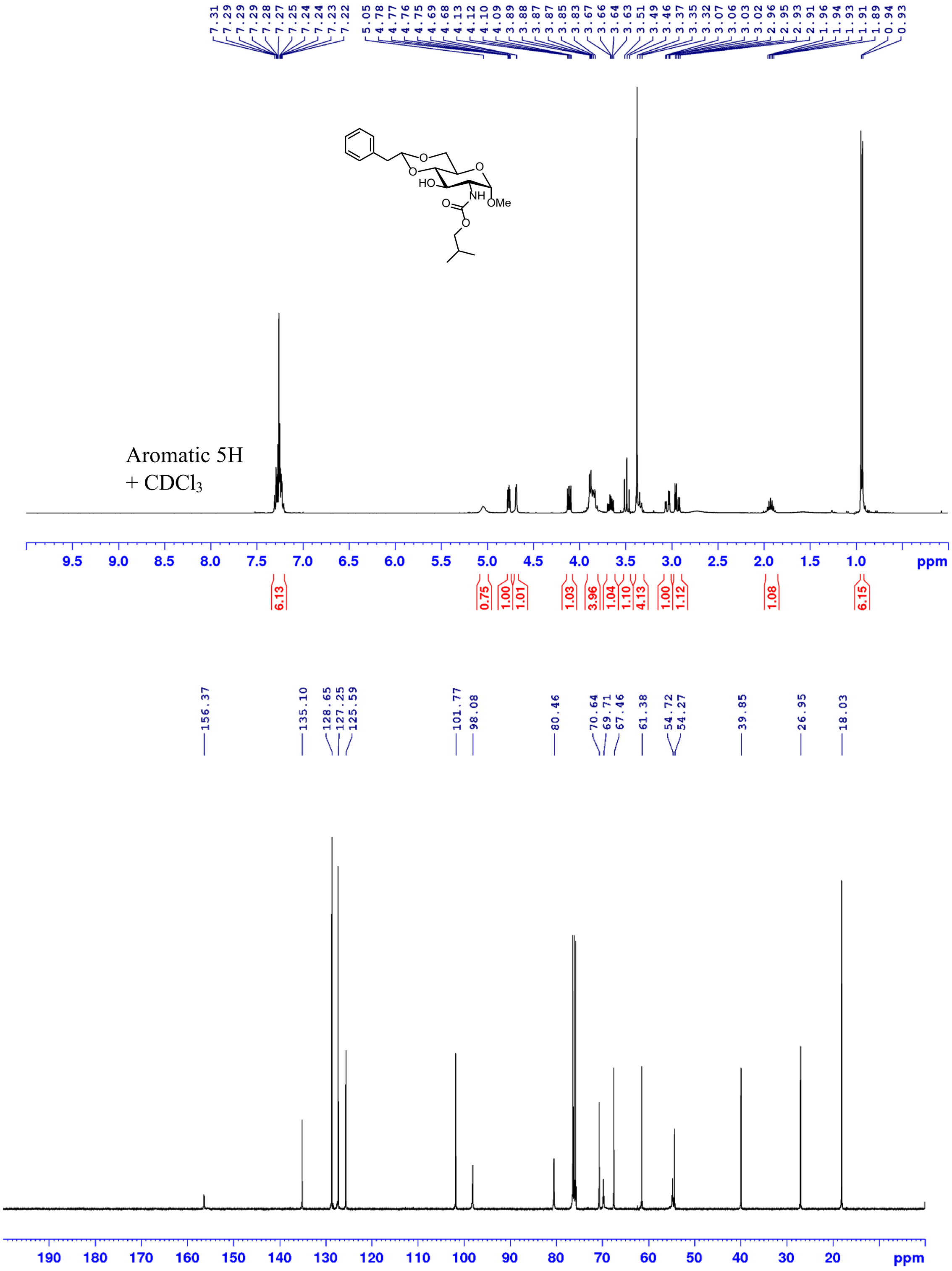

^{1}H NMR and ^{13}C NMR spectra for compound **8** in $CDCl_3$

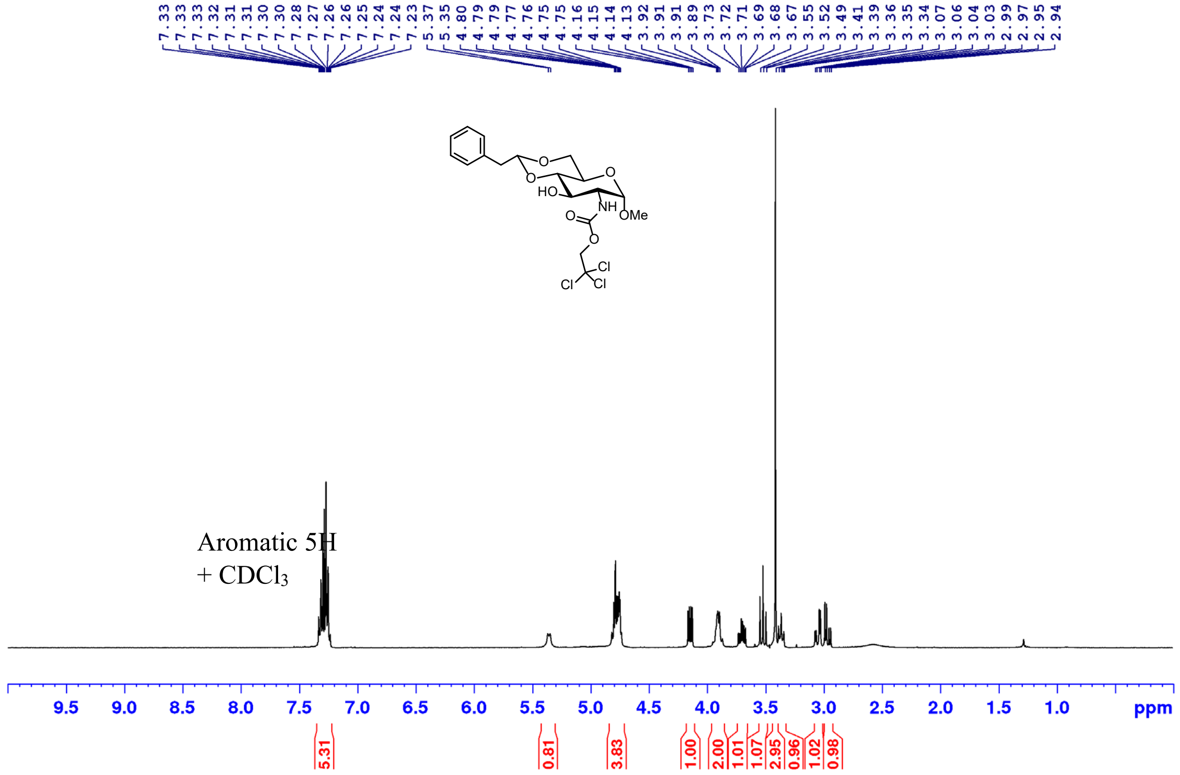

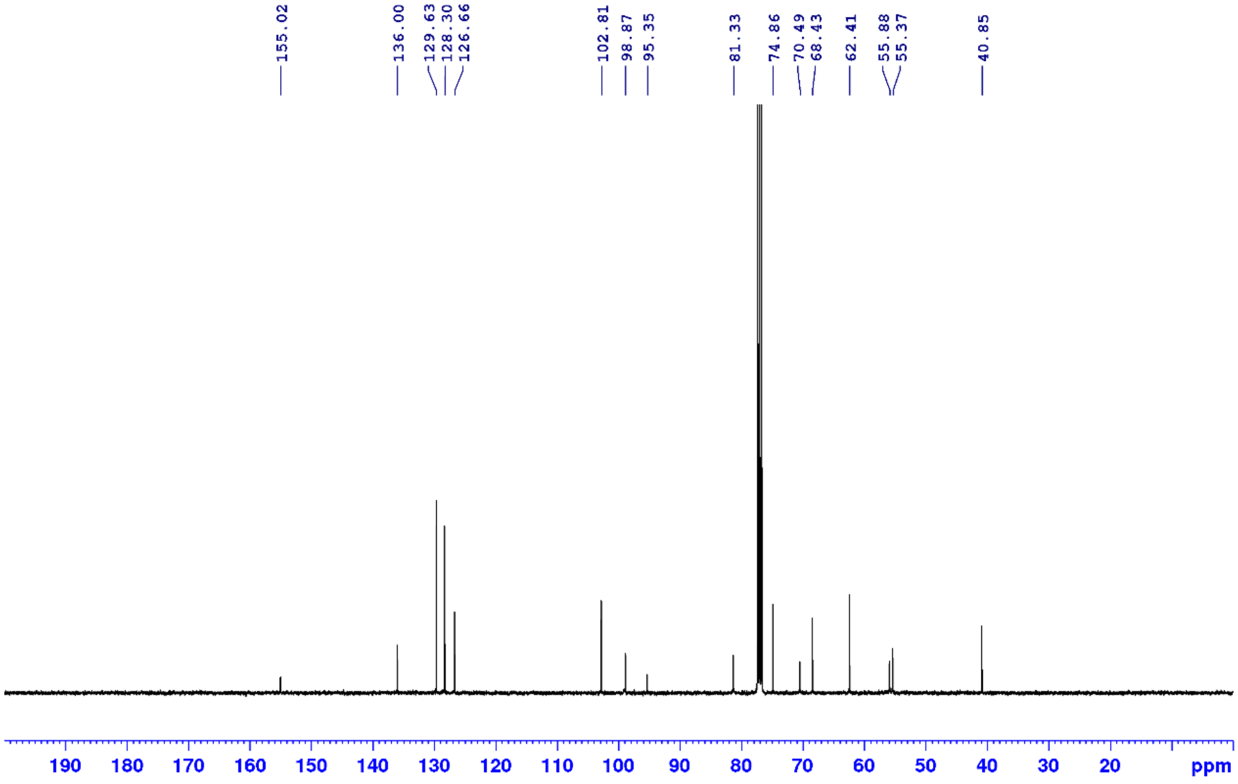

^{1}H NMR and ^{13}C NMR spectra for compound **9** in $CDCl_3$

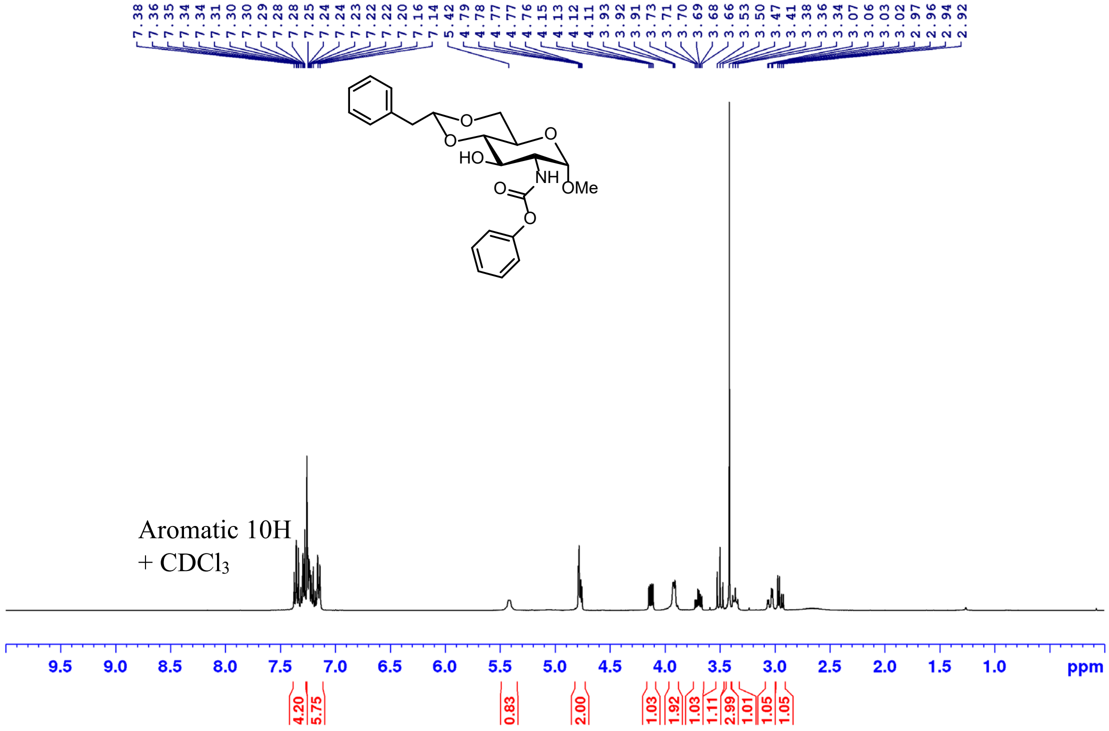

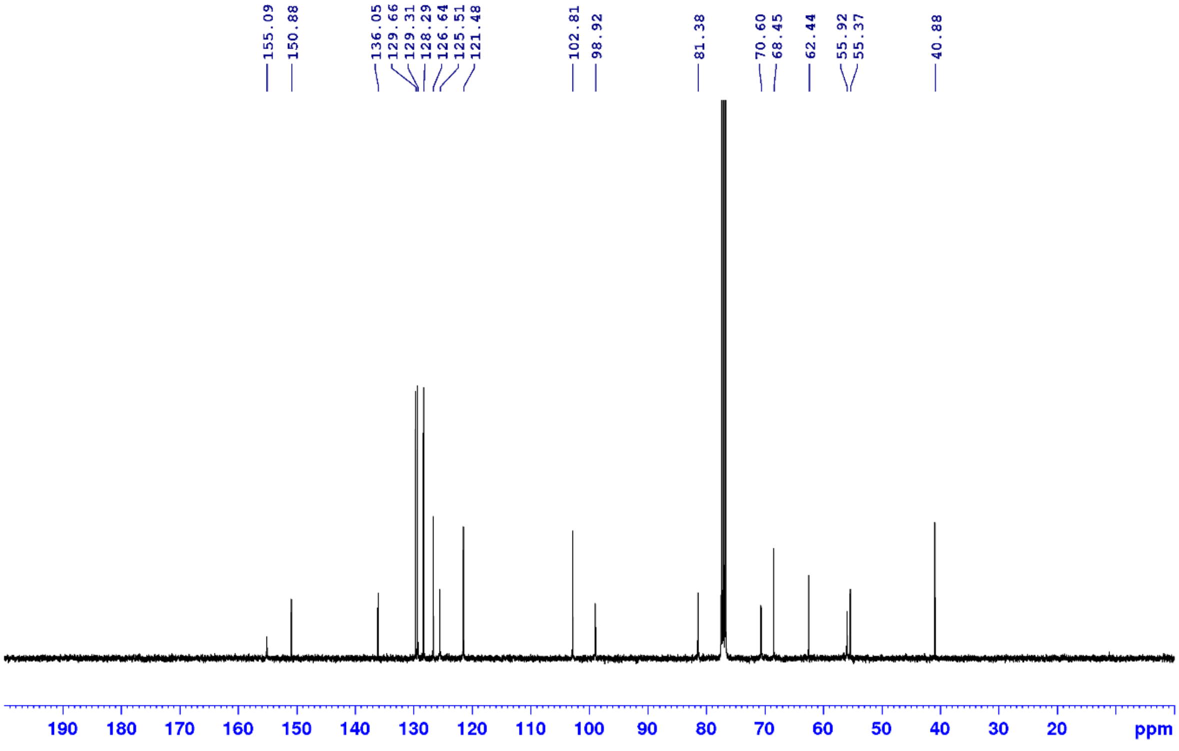

^{1}H NMR and ^{13}C NMR spectra for compound **10** in $CDCl_3$

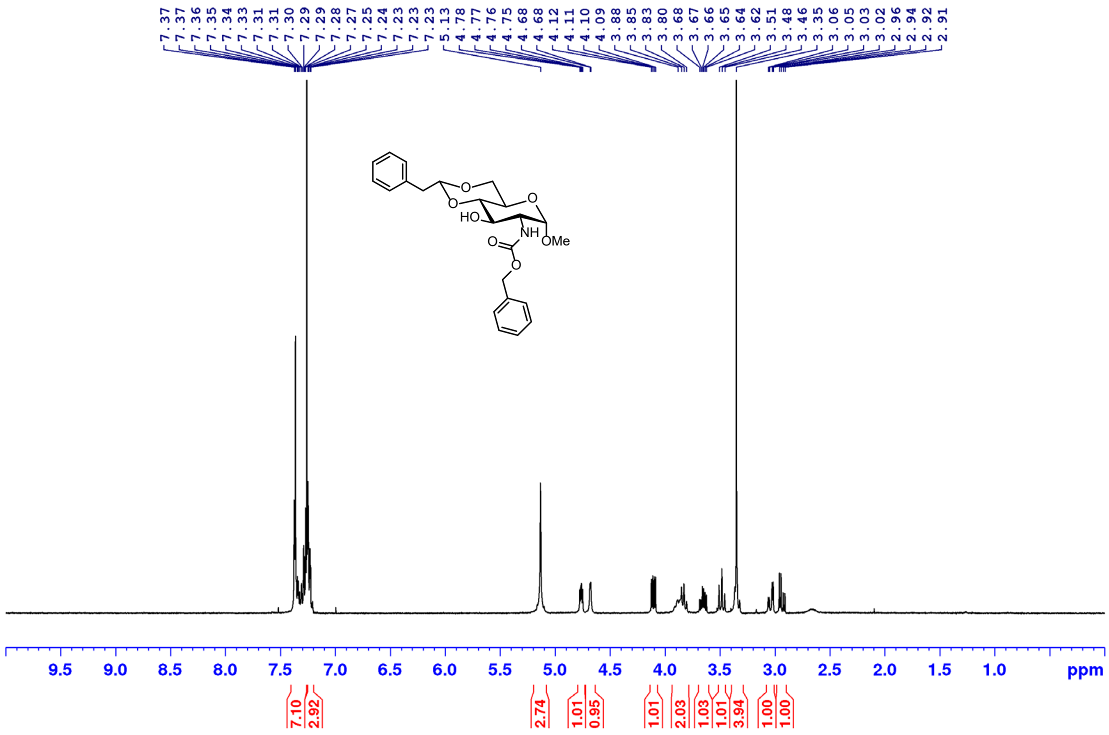

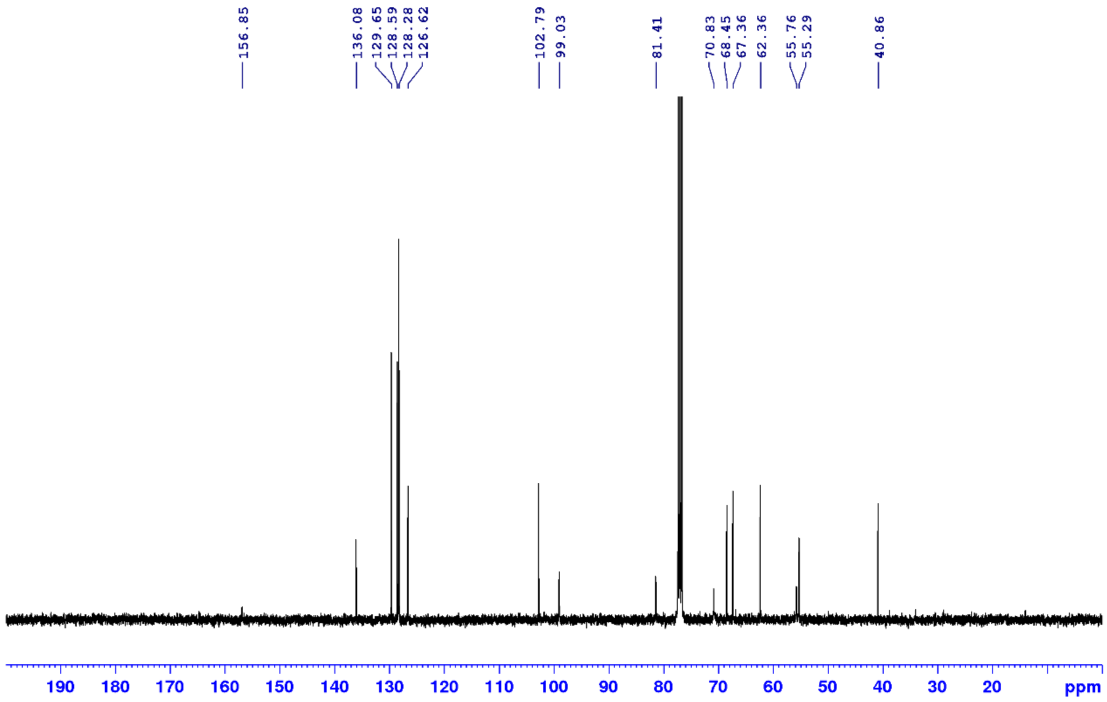

^1H NMR and ^{13}C NMR spectra for compound **11** in $CDCl_3$

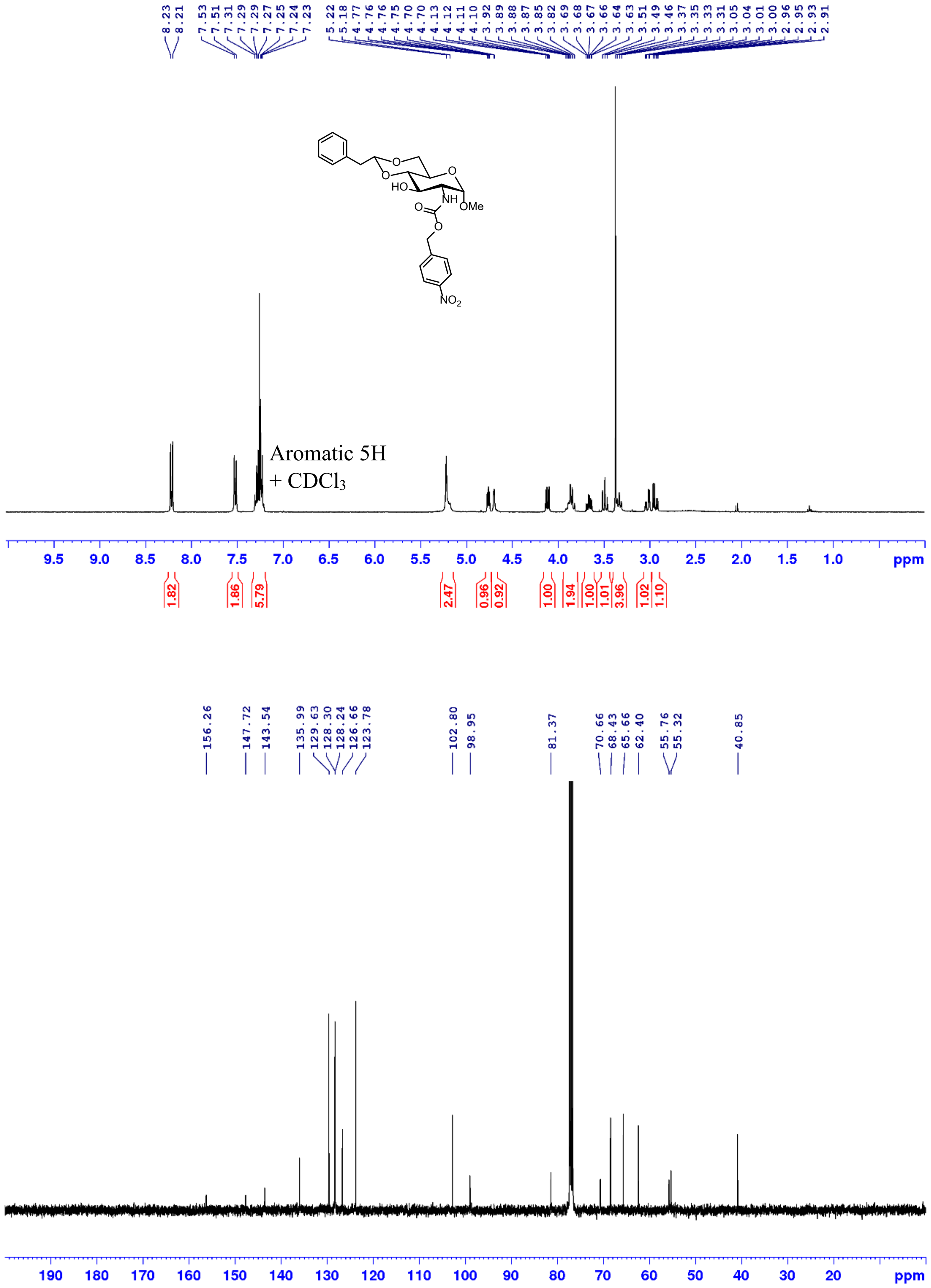

^{1}H NMR and ^{13}C NMR spectra for compound **12** in $CDCl_3$

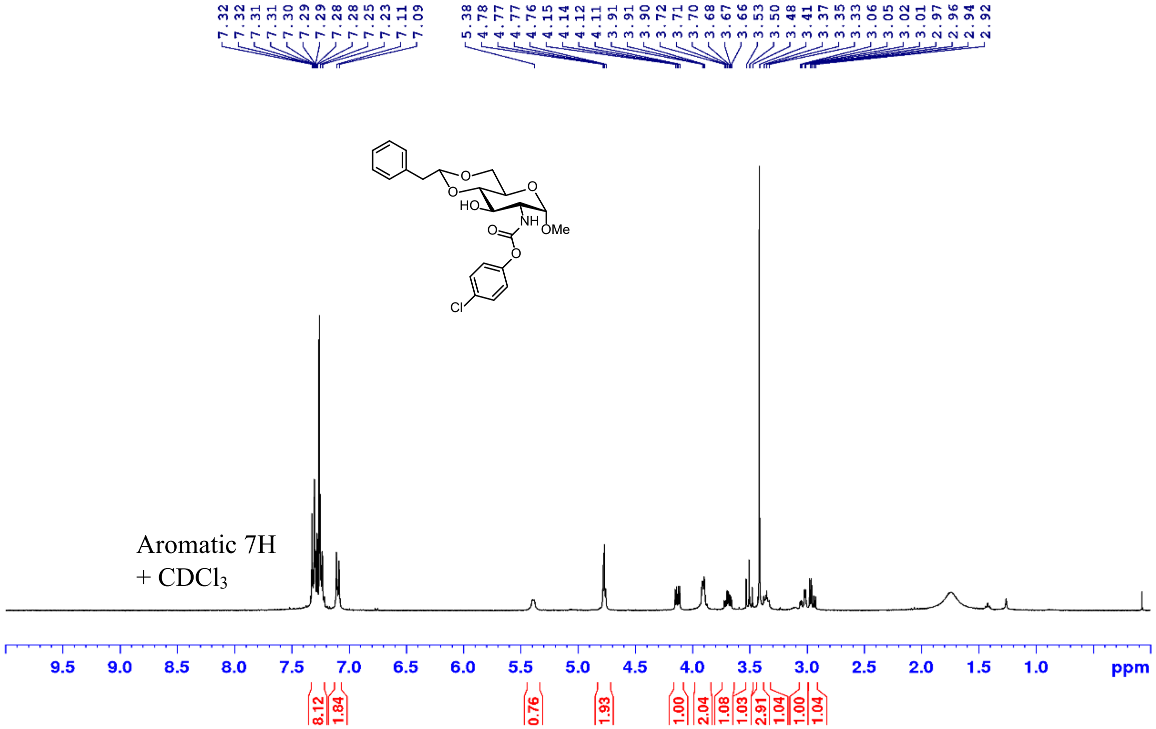

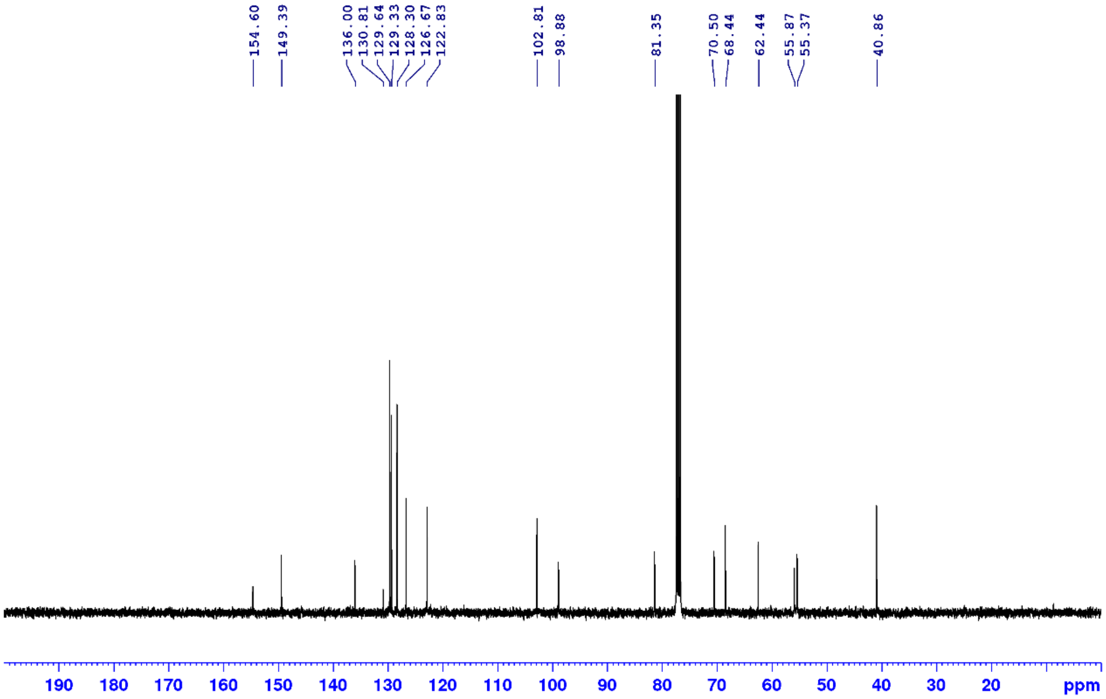

^{1}H NMR and ^{13}C NMR spectra for compound **13** in $CDCl_3$

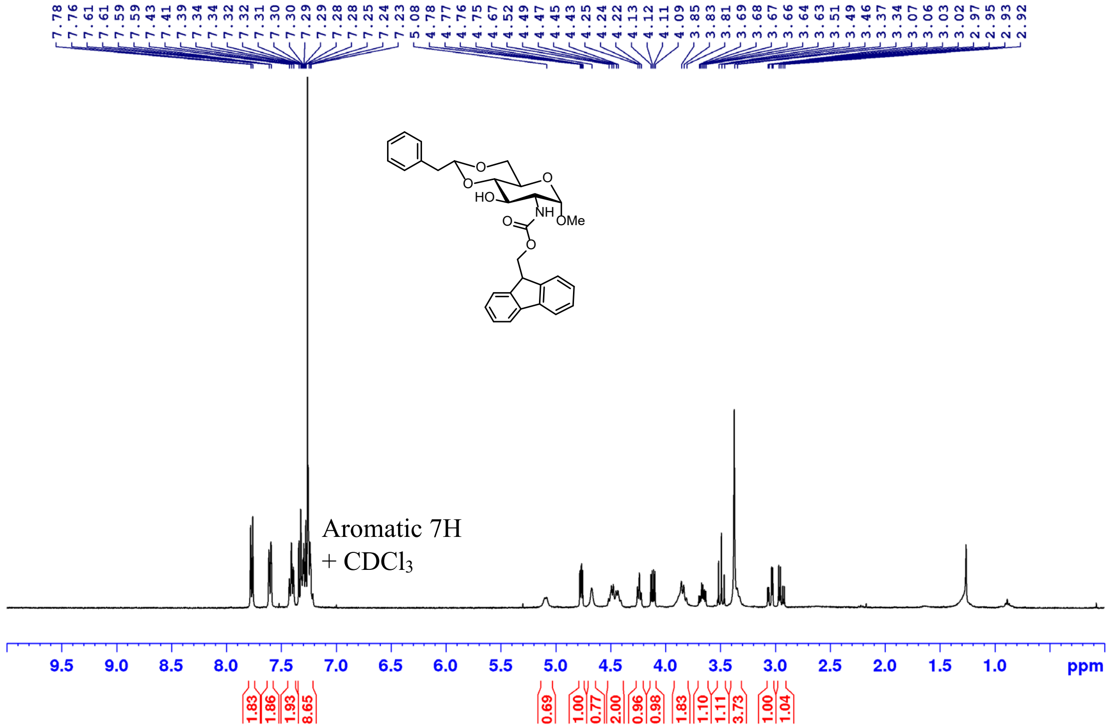

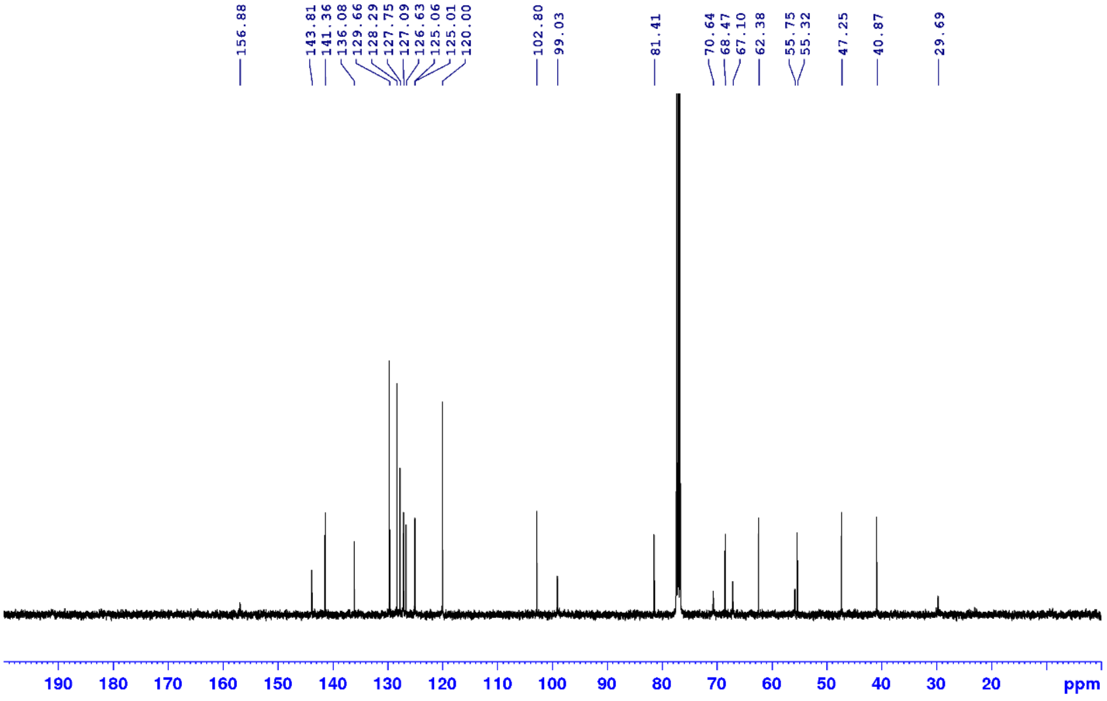

^{1}H NMR and ^{13}C NMR spectra for compound **14** in $CDCl_3$

2D-NMR spectra of compounds 3, 6 and 10

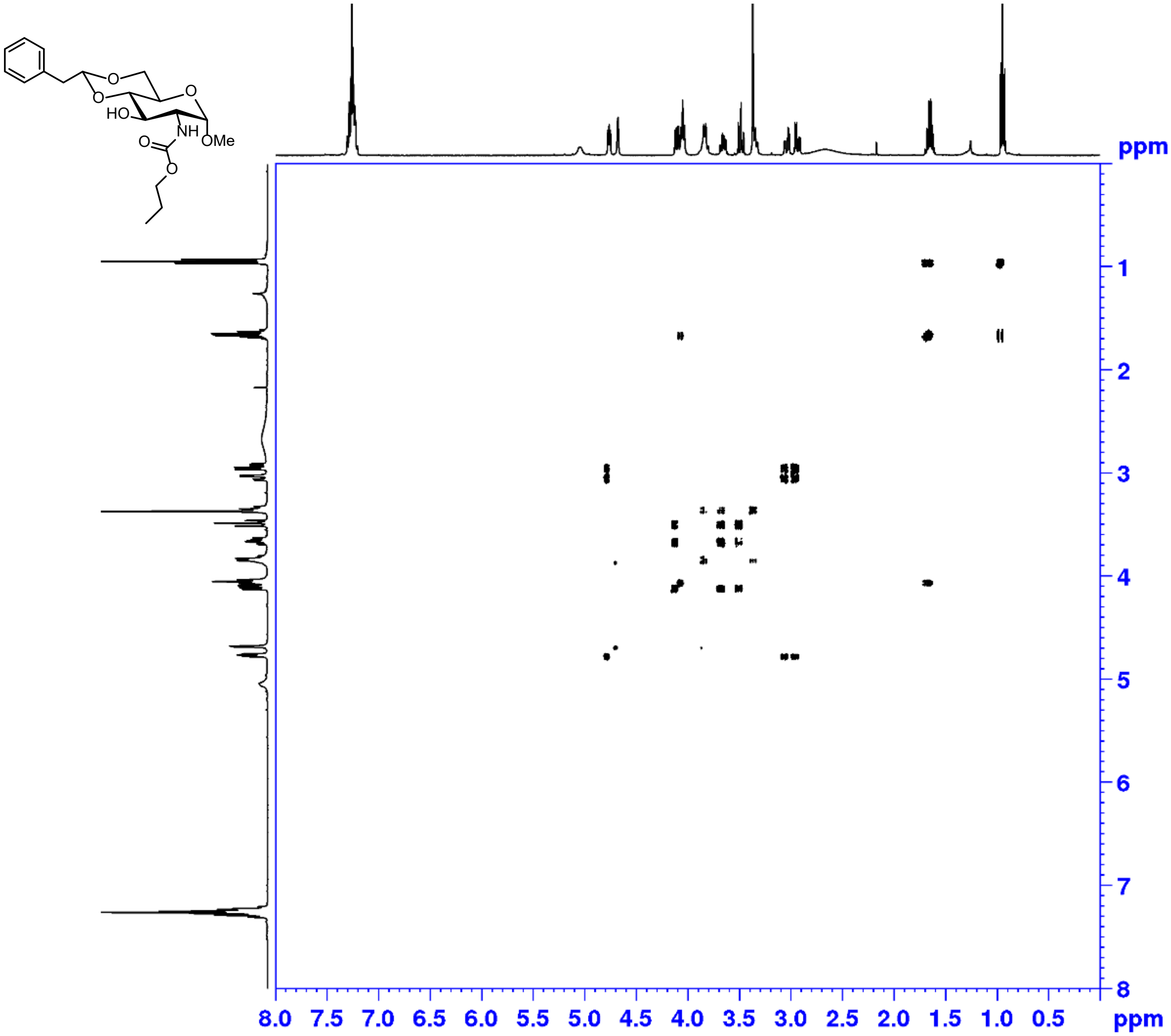

COSY NMR spectrum for compound **3** in $CDCl_3$

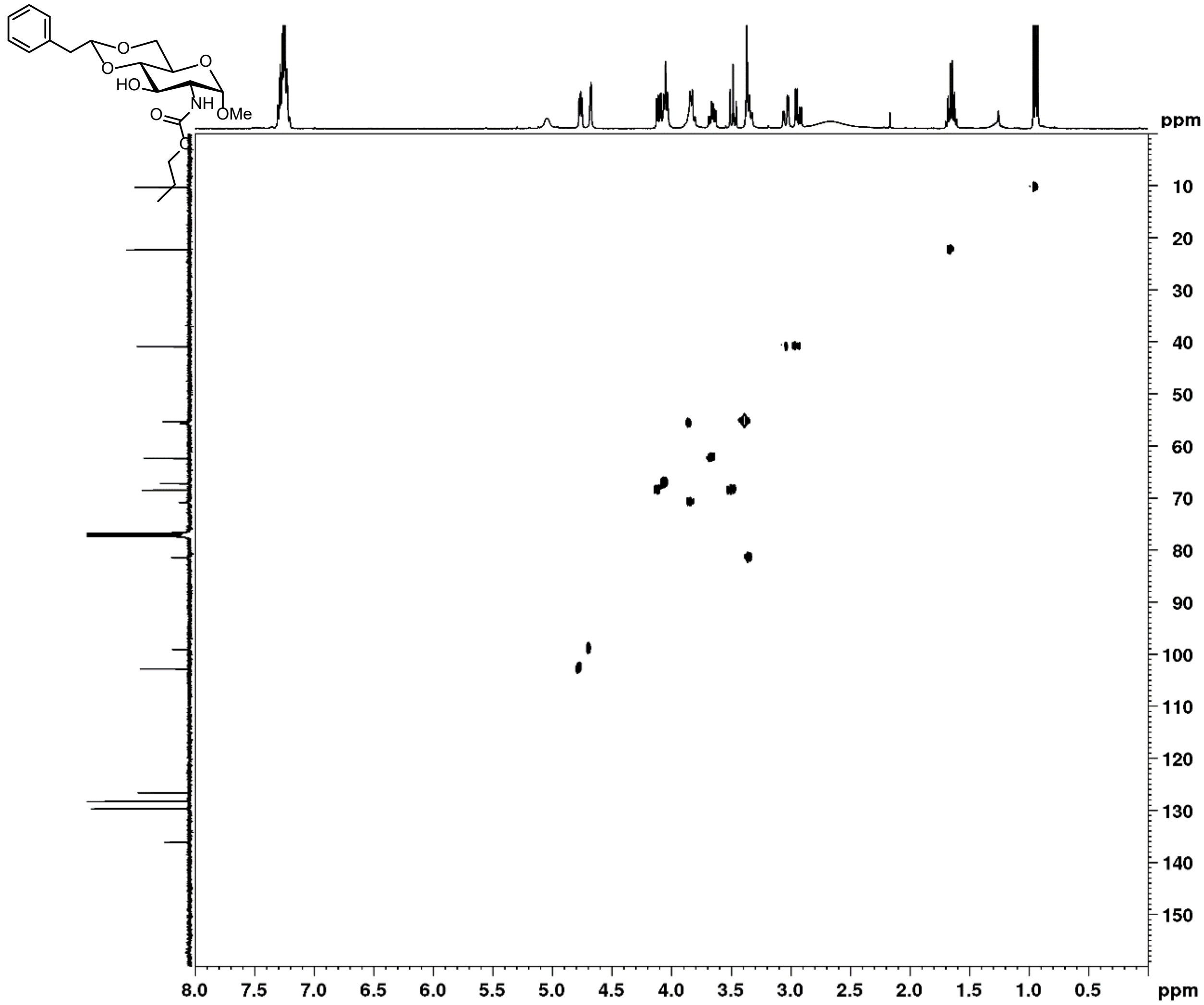

HSQC NMR spectrum for compound **3** in $CDCl_3$

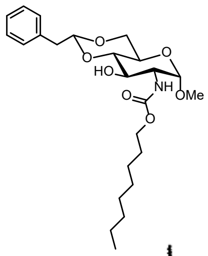

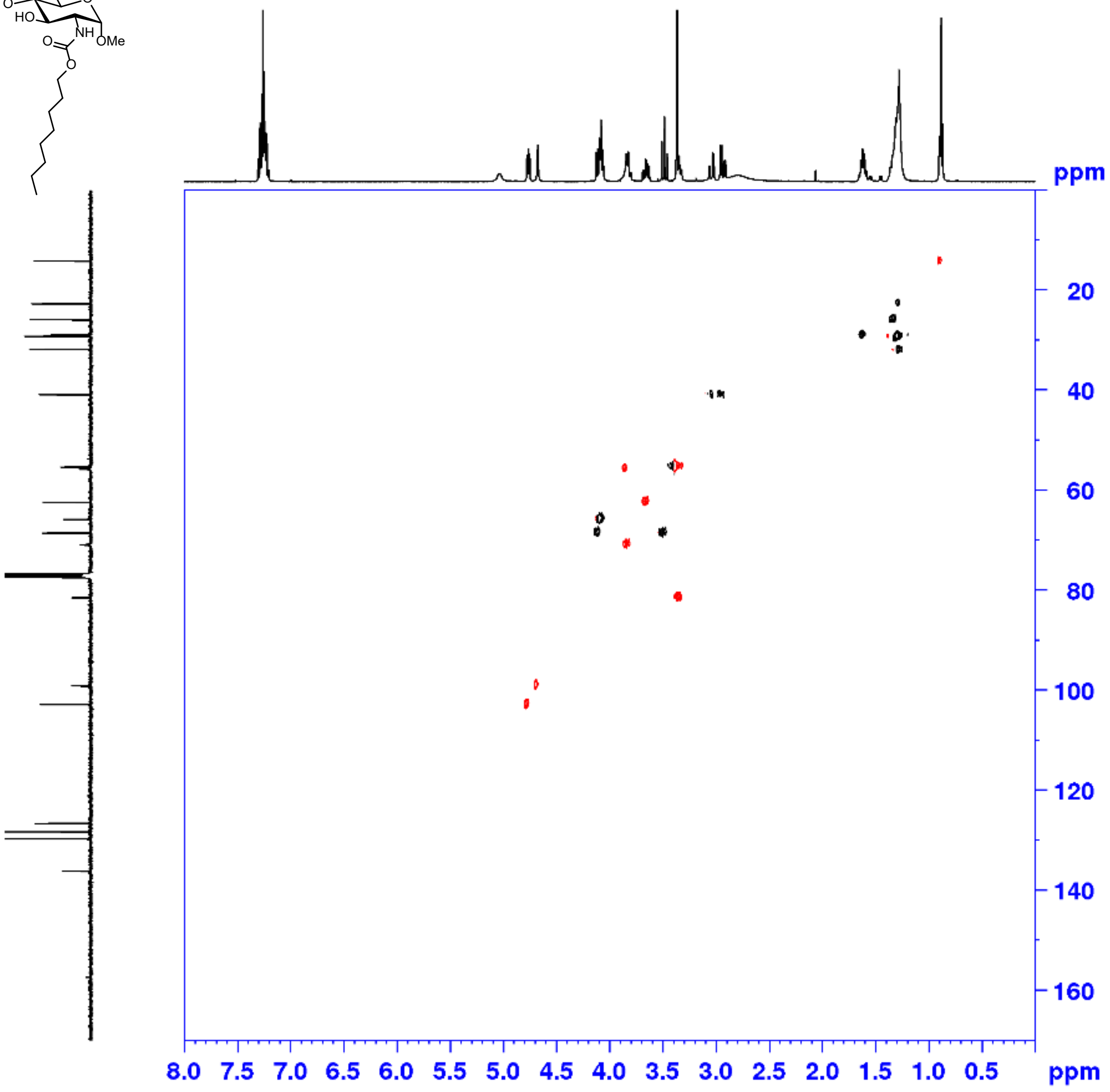

HSQC NMR spectrum for compound **6** in $CDCl_3$

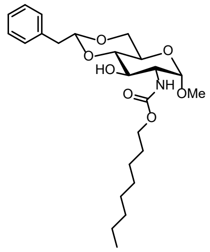

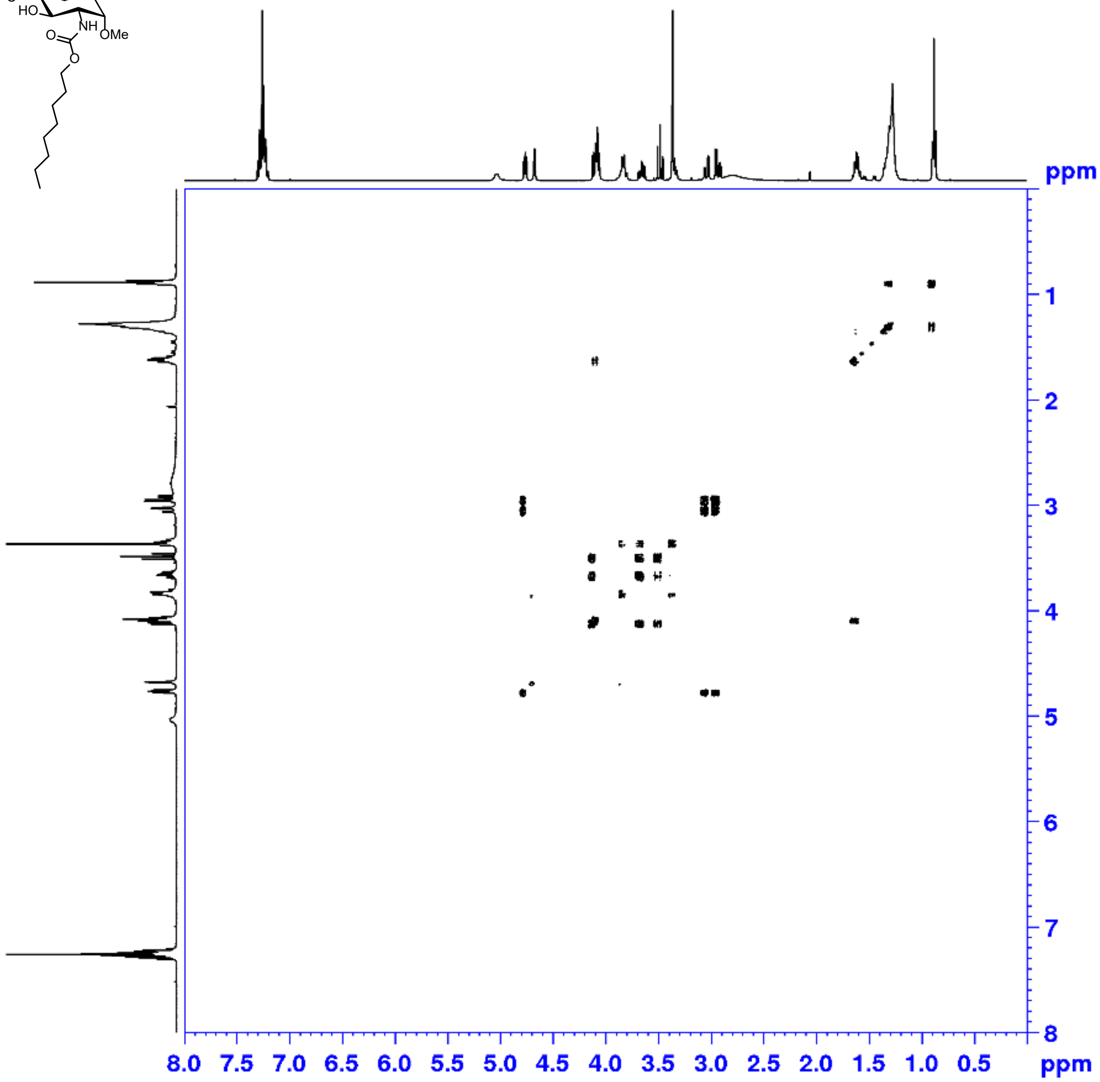

COSY NMR spectrum for compound **6** in $CDCl_3$

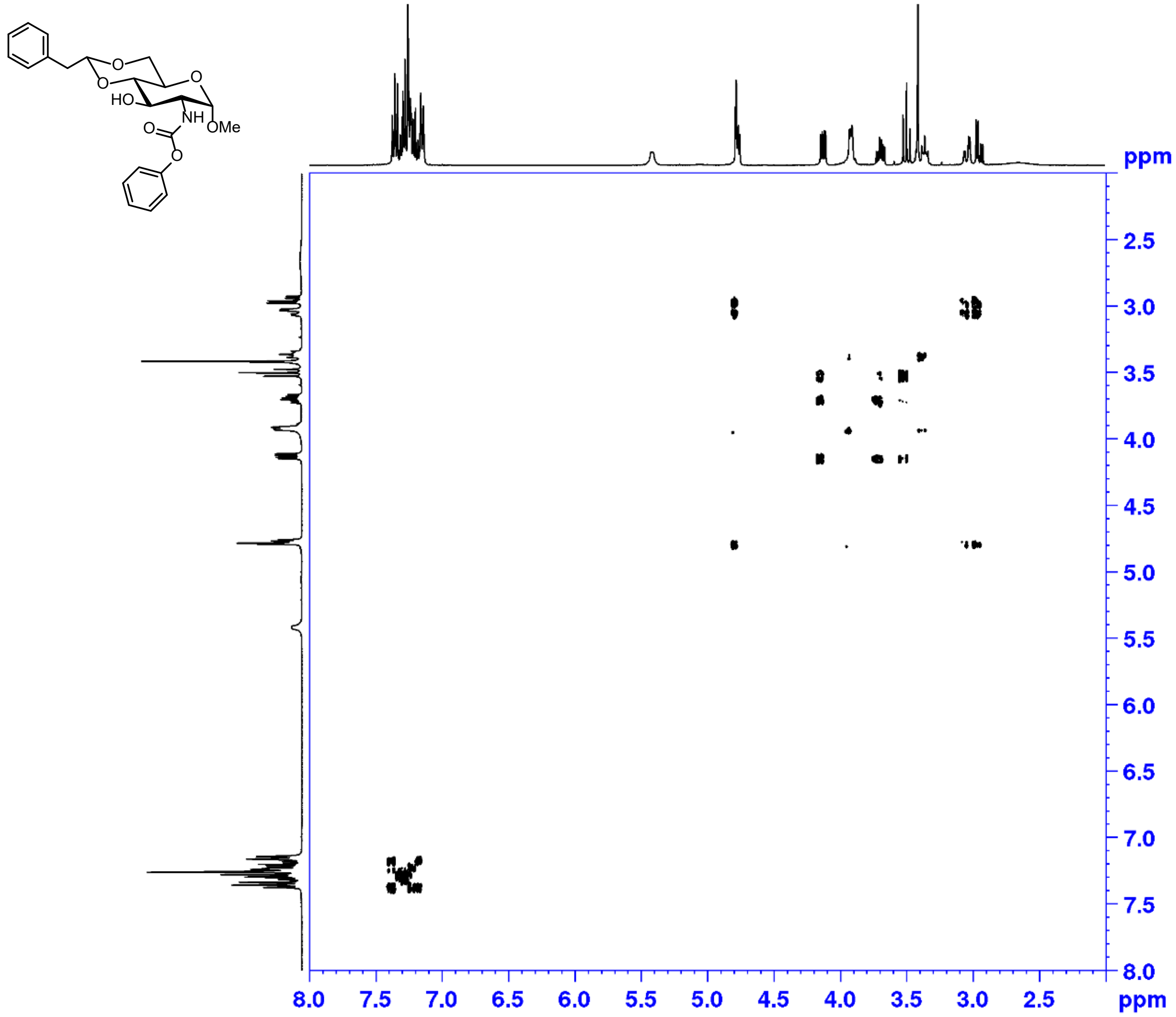

COSY NMR spectrum for compound **10** in $CDCl_3$

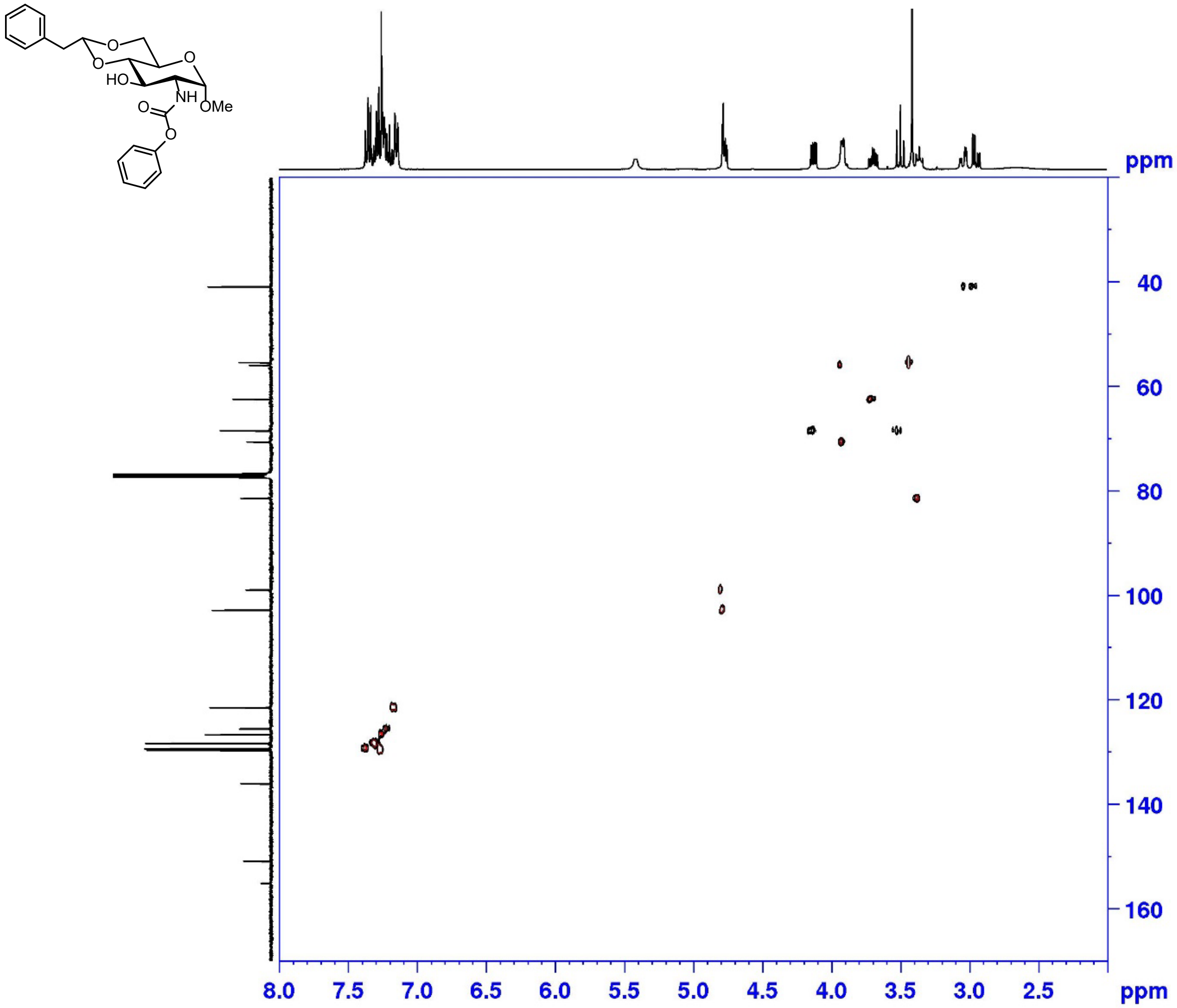

HSQC NMR spectrum for compound **10** in $CDCl_3$

Part II

1. ^{1}H NMR spectra for compound 8 at variable temperatures

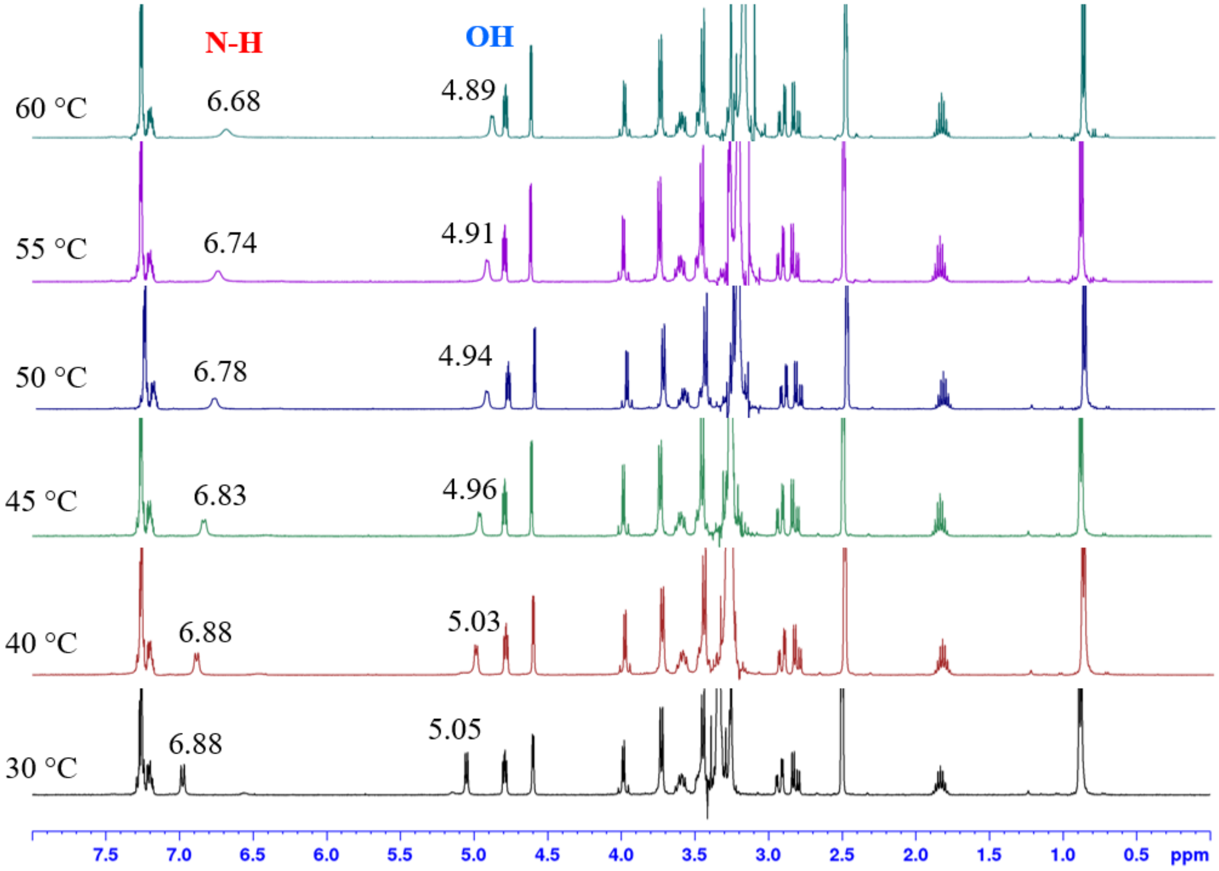

Figure S1. The ^{1}H NMR spectra of compound **8** from 30-60ºC at 8.0 mg/mL in DMSO-d_6.

2. Procedure for gelation tests and water dilution study

About 2.0 mg of dried compound was placed in a 1 dram glass vial and the corresponding solvent was added to obtain a concentration of 20.0 mg/mL. The mixture was then heated until the solid was fully dissolved, sometimes sonication was needed to dissolve the sample, then the solution was allowed to cool to room temperature and let stand for 15-30 minutes. After this period, if the sample is a solution, this is recorded as soluble; if solid reappeared, this is recorded as precipitate; if the sample formed a gel, then the vial is inverted and if there is no solvent flowing this indicated that a stable gel is formed, otherwise this is recorded as unstable gel. The stable gel was then serial diluted till the minimum gelation concentration, which is the concentration prior to unstable gelation, is obtained.

Compound **8** was tested further for solubility/gelation in DMSO aqueous solutions. Typically, 3.0 mg of compound **8** (3.0 mg) was dissolved in 0.1 mL DMSO by heating. To this mixture water was added in 0.1 mL increments. This mixture was heated and cooled to observe gel formation. The photos of these gels formed by compound **8** dissolved in 0.1 mL of DMSO and incremental amounts of water are shown in Figure S2.

Table S1. Gelation properties of compound **8** in different amount of water

**Water Added**	**Result**	**MGC**
0.1 mL	G_T, Instant Gel	
0.2 mL	G_O	
0.3 mL	G_O	
0.4 mL	G_O	
0.5 mL	G_O	G 5.0_O
0.6 mL	Weak G_O	
0.7 mL	Weak G_O	
0.8 mL	Weak G_O	

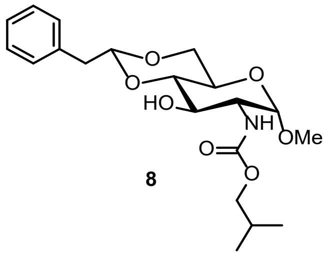

8

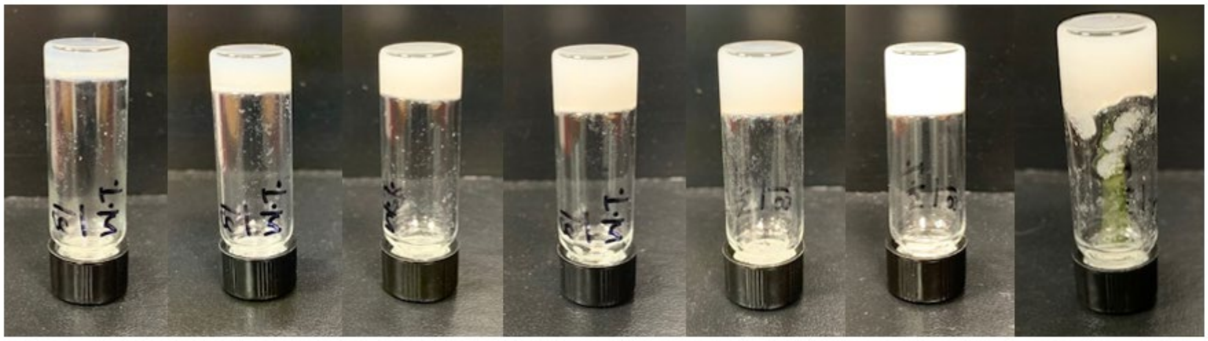

0.1 mL 0.2 mL 0.3 mL 0.4 mL 0.5 mL 0.6 mL 0.7 mL

Figure S2. Gels of compound **8** in 0.1 mL of DMSO and incremental amounts of water.

3. Metallogel formation and characterizations

For the formation of metallogels, in one dram vial, compound **8** (3.0 mg) was dissolved in 0.5 mL DMSO:H_2O (1:5) mixture. The mixture was heated and sonicated to form an opaque gel. To this mixture, 1.5 eq. metal salt was added and the final mixture was heated again and cooled to test gel formation. To the final gel, water was added in 0.1 mL increments and their gelation was tested by heating the mixture and allowing them to cool at room temperature, the gelation properties are shown in Table S2, and the images of gels formed by compound **8** in the presence of 1.5 eq. of metal salt at 5.0 mg/mL are shown in Figure S3a.

Table S2. The gelation concentrations of compound **8** after adding water to metallogels

**Metal salt**	0.1 mL water	0.2 mL water	0.3 mL water	0.4 mL water	0.5 mL water	0.6 mL water	0.7 mL water	0.8 mL water
$Cu(OAc)_2.H_2O$	G 5.0$_O$	G 4.3$_O$	G 3.75$_O$	G 3.3$_O$	G 3.0$_O$	P	-	-
$Cu(SO_4)\cdot 5H_2O$	G 5.0$_O$	G 4.3$_O$	G 3.75$_O$	G 3.3$_O$	G 3.0$_O$	P	-	-
$CuBr_2$	G 5.0$_O$	G 4.3$_O$	G 3.75$_O$	G 3.3$_O$	G 3.0$_O$	P	-	-
$Hg(OAc)_2$	No gel	-	-	-	-	-	-	-
$Zn(OAc)_2.2H_2O$	G 5.0$_O$	G 4.3$_O$	G 3.75$_O$	G 3.3$_O$	G 3.0$_O$	G 2.7$_O$	G 2.5$_O$	P
$NiCl_2.6H_2O$	G 5.0$_O$	G 4.3$_O$	G 3.75$_O$	G 3.3$_O$	G 3.0$_O$	G 2.7$_O$	G 2.5$_O$	P
$Pb(OAc)_4$	G 5.0$_O$	G 4.3$_O$	G 3.75$_O$	P	-	-	-	-
$FeCl_2$	G 5.0$_O$	G 4.3$_O$	G 3.75$_O$	G 3.3$_O$	G 3.0$_O$	P	-	-
$FeCl_3\cdot 6H_2O$	G 5.0$_O$	G 4.3$_O$	G 3.75$_O$	G 3.3$_O$	G 3.0$_O$	P	-	-
$AgNO_3$	G 5.0$_O$	G 4.3$_O$	G 3.75$_O$	P	-	-	-	-

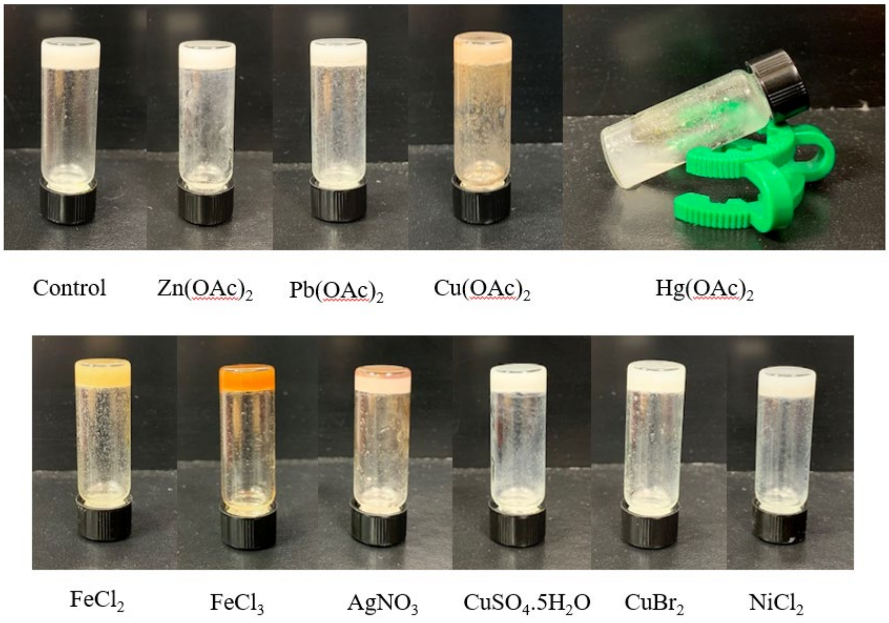

Figure S3a. Gels formed by compound **8** with 1.5 eq. of metal salt at 5.0 mg/mL.

Similarly, compound **11** was also able to form metallogels in the presence of 1.0 eq. metal salt in DMSO:H_2O (1:5) mixture when tested at 3.0 mg/mL concentration, the gel photos are shown in Figure S3b.

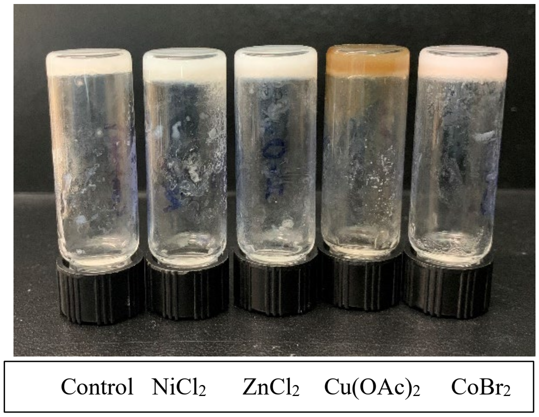

Figure S3b. Gels formed by compound **11** with 1.0 eq. of metal salt at 3.0 mg/mL.

IR analysis of metallogels In a one dram vial, compound **8** (3.0 mg) was dissolved in 0.5 mL DMSO:H_2O (1:5) mixture. The mixture was heated and sonicated to form an opaque gel. To this mixture, 1.0 eq. metal salt was added and the final mixture was heated again and cooled to test gel formation. A small piece of gel was placed on the disc of Bruker Alpha FTIR spectrometer and analyzed using OPUS software.

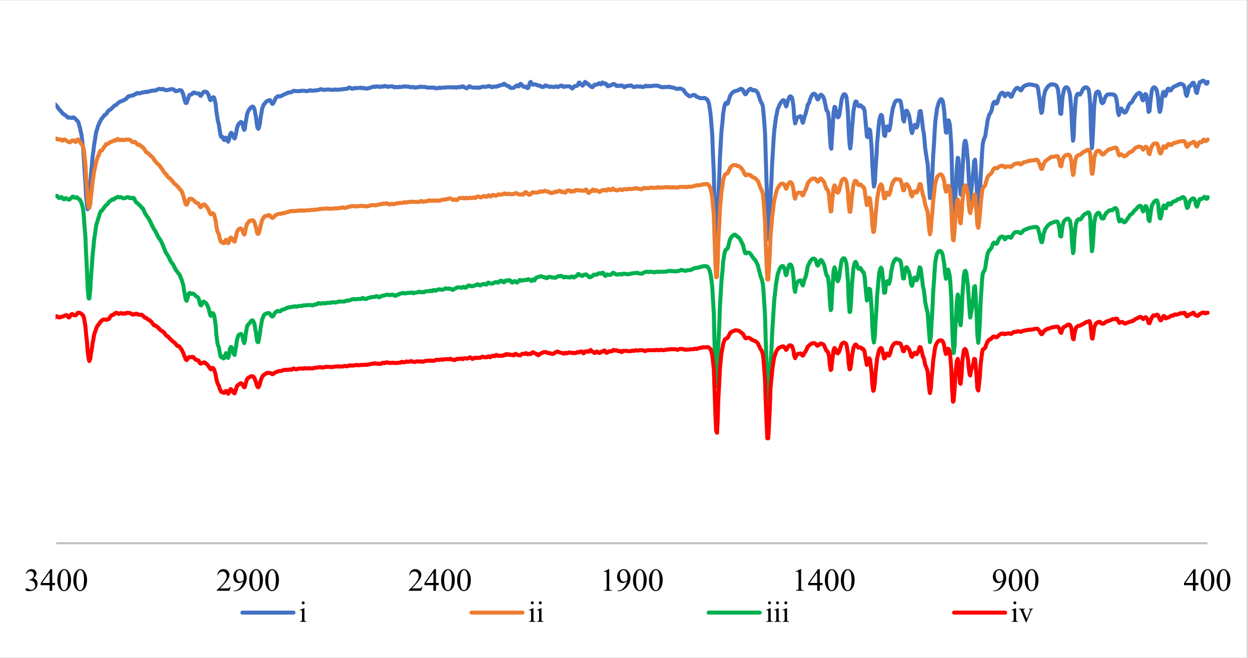

Figure S3c. Overlay of IR spectra of compound **8**.

i) **8** as solid; ii) Gel formed by **8** in DMSO:H_2O (1:5) at 6.0 mg/mL as control; iii) Metallogel formed by **8** and $Zn(OAc)_2{\cdot}2H_2O$ (1.5 eq.) at 2.5 mg/mL; iv) Metallogel formed by **8** and $Cu(OAc)_2{\cdot}H_2O$ (1.5 eq.) at 3.0 mg/mL.

4. Rheological analysis data

The data was collected for various gels at their minimum gelation concentrations. The gels were typically left standing for 15-18 h. Strain used for frequency sweeps was 0.2% (based on Linear Viscoelastic Range of amplitude sweeps). The amplitude sweep results for a few gels are shown in Figure S4a-c.

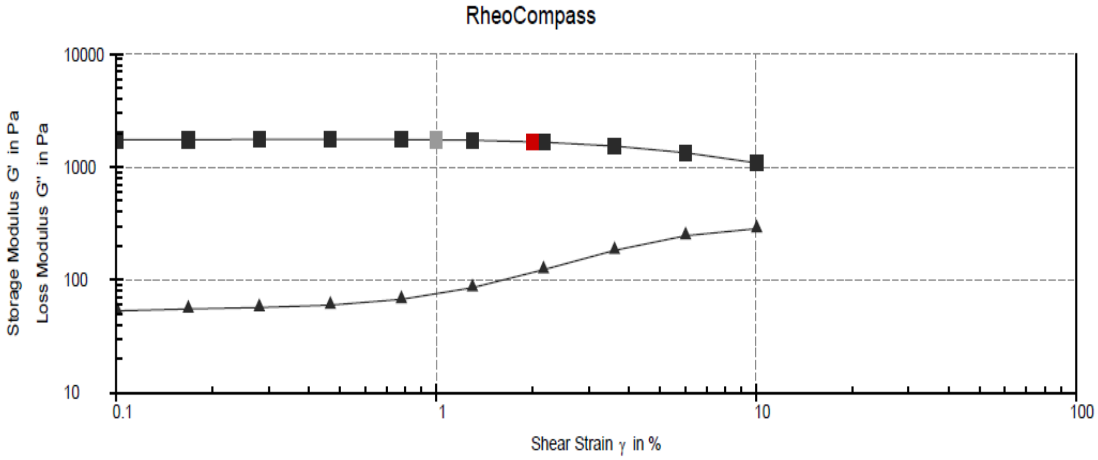

Figure S4a. Amplitude sweep results for **c**ompound **7** at 2.0 mg/mL in H_2O.

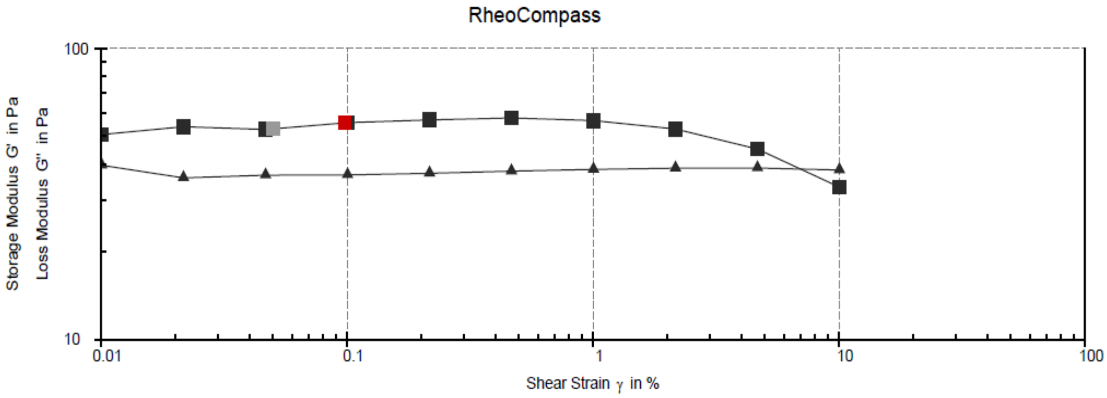

Figure S4b. Amplitude sweep results for compound **11** at 1.3 mg/mL in glycerol.

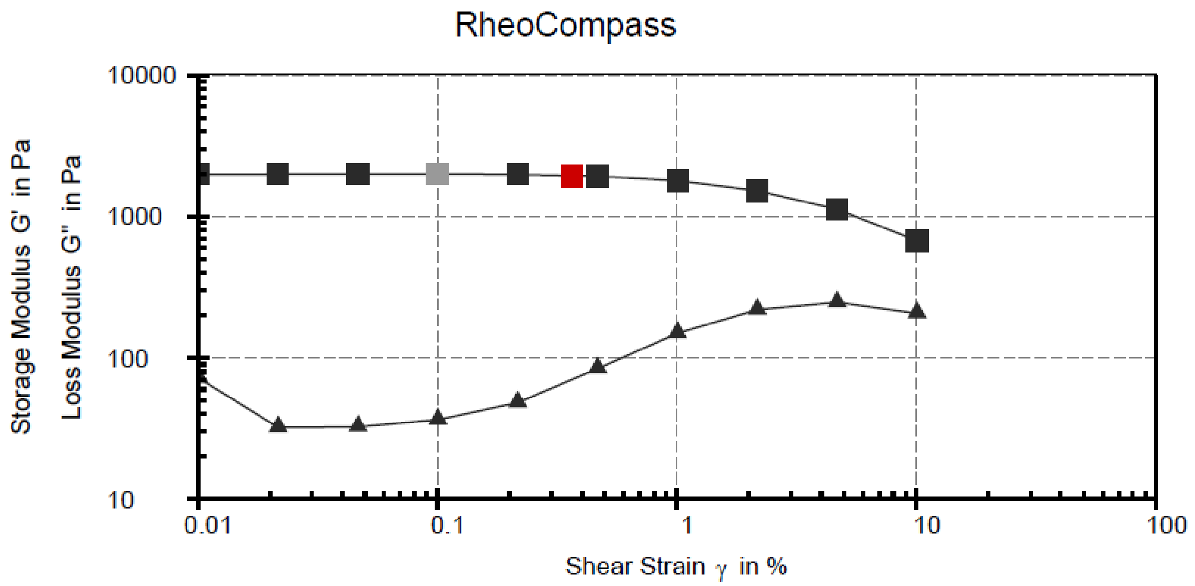

Figure S4c. Amplitude sweep results for compound **12** at 1.8 mg/mL in DMSO:H_2O (1:2).

Table S3. G'/G" ratios for several gels

Angular Frequency	**7** Storage Modulus (G')	**7** Loss Modulus (G")	G'/G"	**11** Storage Modulus (G')	**11** Loss Modulus (G")	G'/G"	**12** Storage Modulus (G')	**12** Loss Modulus (G")	G'/G"
[rad/s]	[Pa]	[Pa]		[Pa]	[Pa]		[Pa]	[Pa]	
100.0	44371.0	1532.5	29.0	212.3	217.9	1.0	9448.5	261.6	36.1
46.4	43747.0	1481.2	29.5	167.9	121.8	1.4	9437.4	122.0	77.4
21.5	43328.0	1659.6	26.1	153.3	74.5	2.1	9427.4	129.2	73.0
10.0	42851.0	1901.0	22.5	142.2	48.9	2.9	9394.7	164.7	57.1
4.6	42376.0	2176.4	19.5	134.8	34.7	3.9	9329.5	165.7	56.3
2.2	41880.0	2502.6	16.7	128.6	27.6	4.7	9432.0	233.2	40.4
1.0	41682.0	2849.4	14.6	128.6	23.0	5.6	9456.5	283.7	33.3
0.5	41376.0	3150.8	13.1	127.5	21.1	6.1	9647.0	404.2	23.9
0.2	41257.0	3689.1	11.2	126.6	20.5	6.2	9657.1	781.8	12.4
0.1	40915.0	4130.8	9.9	129.5	23.6	5.5	9782.5	736.3	13.3
**Average G'/G"**			**19.2**			**3.9**			**42.3**

Rheological data for metallogels

For metallogels, the age of gels was 4-6 hr. Strain used for frequency sweeps was 0.2% (based on Linear Viscoelastic Range of amplitude sweeps.

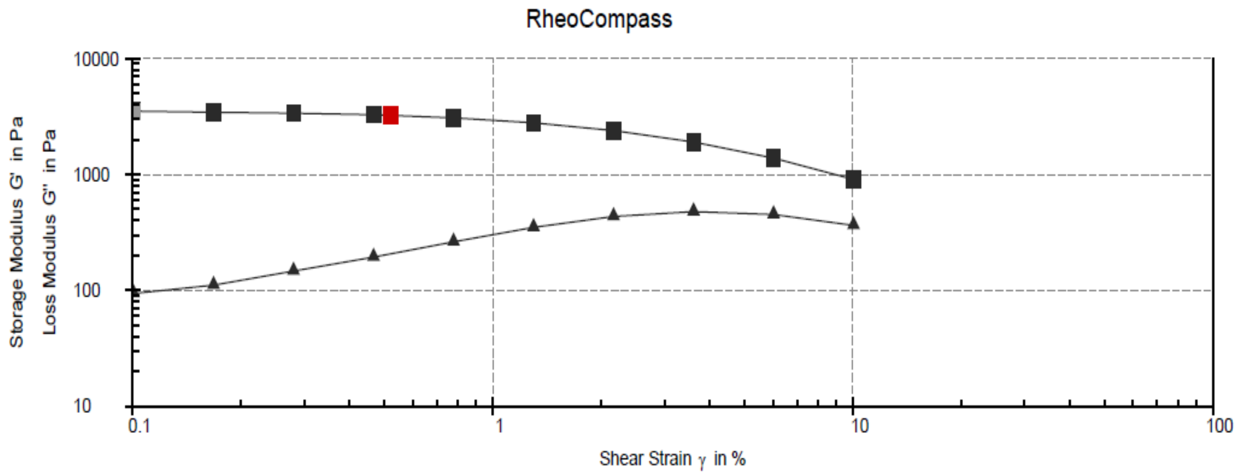

Figure S4d. Amplitude sweep results for Compound **8** at 6.0 mg/mL in DMSO:H_2O (1:5) (Control).

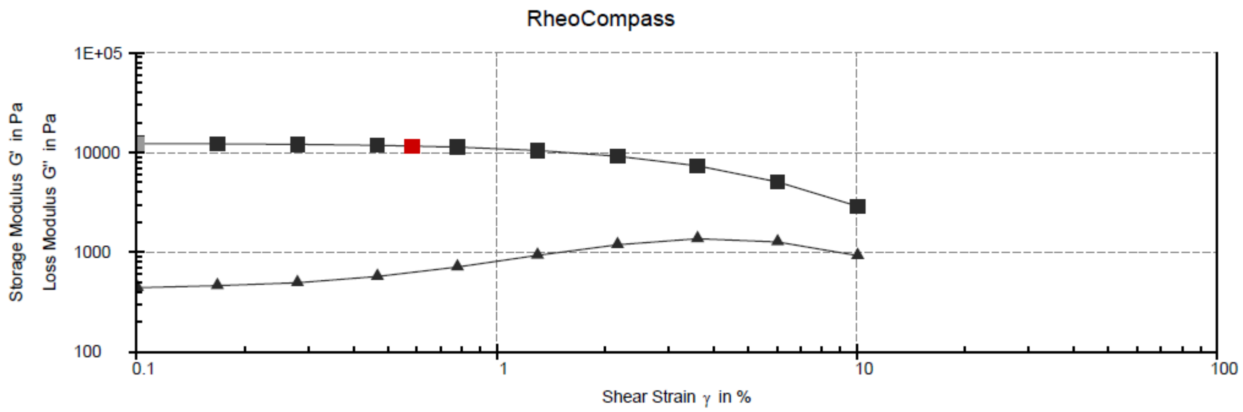

Figure S4e. Amplitude sweep results for metallogel of compound **8** and $Cu(OAc)_2$ (1.5 eq.) at 3.0 mg/mL in DMSO:H_2O (1:5).

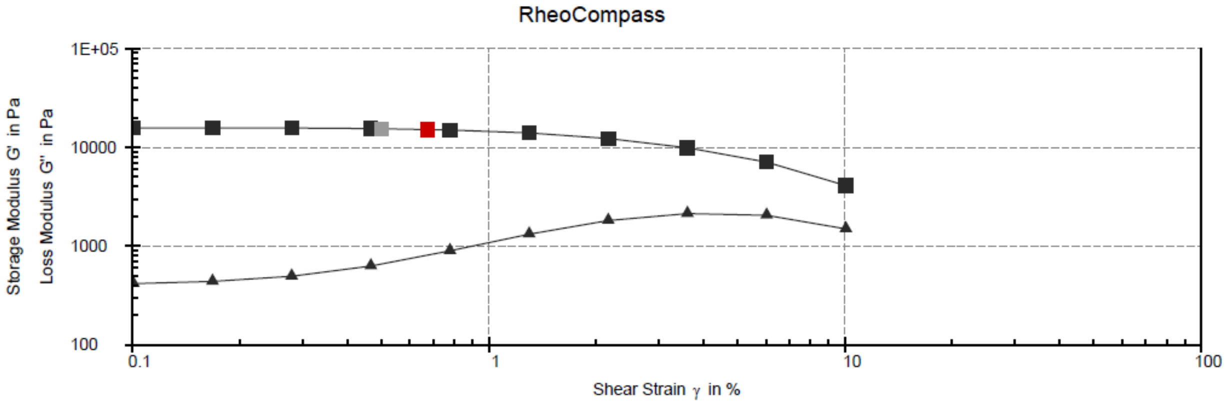

Figure S4f. Amplitude sweep results for metallogel of compound **8** and $NiCl_2$ (1.5 eq.) at 2.5 mg/mL in DMSO:H_2O (1:5).

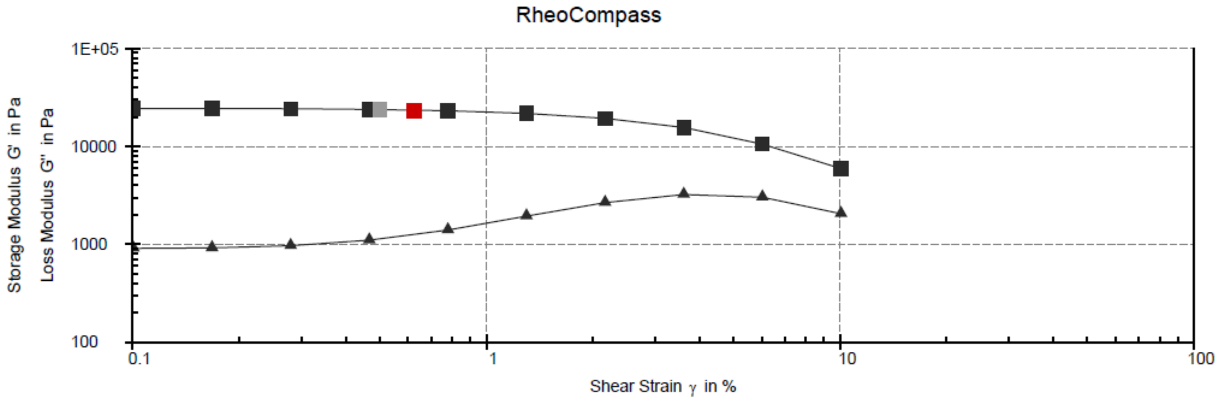

Figure S4g. Amplitude sweep results for metallogel of compound **8** and $Zn(OAc)_2$ (1.5 eq.) at 2.5 mg/mL in DMSO:H_2O (1:5).

Table S4a. G'/G" ratios for various gels and metallogels.

Angular Frequency (ω)	**8**-Control Storage Modulus (G')	**8**-Control Loss Modulus (G")	G'/G"	**8**+Cu Storage Modulus (G')	**8**+Cu Loss Modulus (G")	G'/G"
[rad/s]	[Pa]	[Pa]		[Pa]	[Pa]	
100	30890	1147.7	26.9	112110.0	3431.3	32.7
46.4	30586	975.52	31.4	112140.0	3842.6	29.2
21.5	30510	1081.1	28.2	112040.0	4270.1	26.2
10	30427	1136	26.8	111580.0	4908.8	22.7
4.64	30301	1212.6	25.0	111280.0	5605.1	19.9
2.15	30257	1372.2	22.0	110840.0	6430.7	17.2
1	30383	1536.4	19.8	110150.0	7720.7	14.3
0.464	30591	1544.3	19.8	108980.0	9107.4	12.0
0.215	30641	1647.8	18.6	106760.0	11202	9.5
0.1	30759	2334.5	13.2	102520.0	12434	8.2
Average G'/G"			23.2			19.2

Table S4b. G'/G" ratios for metallogels.

Angular Frequency (ω)	**8**+Ni Storage Modulus (G')	**8**+Ni Loss Modulus (G")	G'/G"	**8**+Zn Storage Modulus (G')	8+Zn Loss Modulus (G")	G'/G"
[rad/s]	[Pa]	[Pa]		[Pa]	[Pa]	
100	146950.0	6916.2	21.2	182980.0	5915.7	30.9
46.4	146590.0	6717.1	21.8	180960.0	4976.8	36.4
21.5	145630.0	7486.2	19.5	179130.0	5555.7	32.2
10	144700.0	8519.4	17.0	177060.0	6525.3	27.1
4.64	143630.0	9598.7	15.0	174800.0	7704.7	22.7
2.15	142660.0	11412.0	12.5	172460.0	9146.7	18.9
1	142070.0	13196.0	10.8	169040.0	11155.0	15.2
0.464	142410.0	14849.0	9.6	166140.0	14743.0	11.3
0.215	142090.0	17383.0	8.2	160990.0	17300.0	9.3
0.1	140550.0	20380.0	6.9	154520.0	23497.0	6.6
Average G'/G"			14.2			21.1

5. Chemiluminescence analysis

In a one dram vial, gelator **8** (3.0 mg, 1.0 eq.) and metal salt (1.0 eq.) were added. Then, DMSO:H_2O (1:5) mixture (0.5 mL) was added into the vials. The mixture was heated to form a homogeneous solution and allowed to cool to room temperature to form gels. Subsequently, metal−organic gels were observed within 15 min under ambient conditions. Metal Organic Xerogels (MOX) were further acquired after removing the solvent of MOGs under air. To investigate the luminol− MOXs CL system, 100 µL of solution of MOX (3.0 mg/mL premixed in Millipore grade water) was added in 96-well plate. Then, 100 µL of the luminol solution (2.0 mg/mL in 10.0 mM NaOH solution) was injected followed by 100 µL of H_2O_2 solution (0.15%), and the CL profile and intensity were measured.

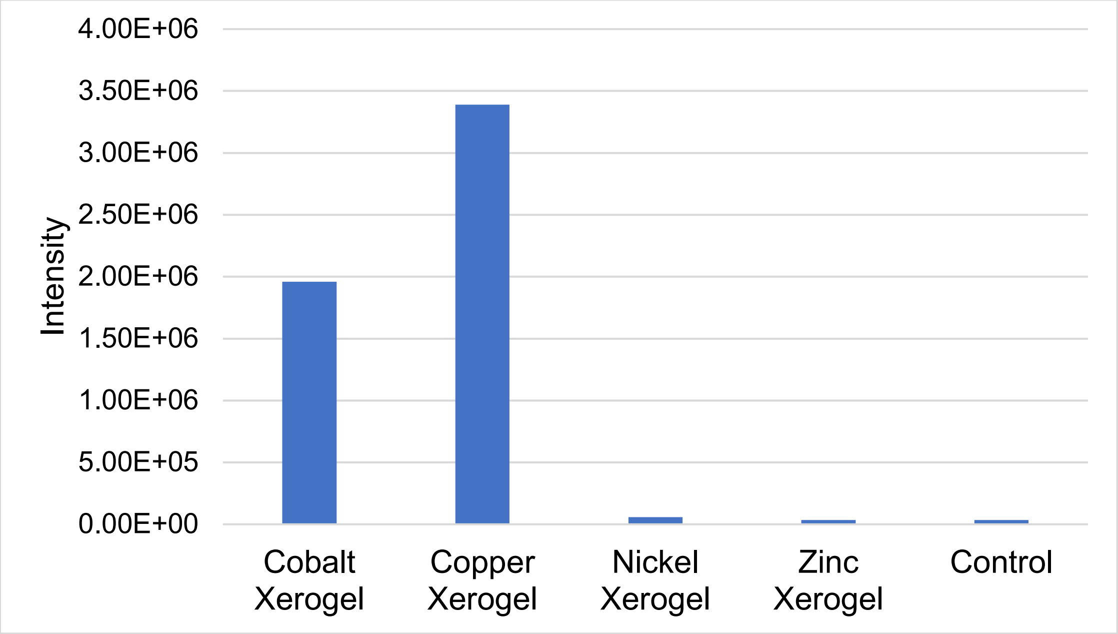

Figure S5. Chemiluminescence intensities of luminol with Metal-Organic-Xerogels of compound **8**. Control: Metallogel (3.0 mg/mL in DMSO:H_2O (1:5))+ Luminol (100 µL of 2.0 mg/mL solution in 10 mM NaOH). Metal-Organic-Xerogel (3.0 mg/mL in MilliQ water) + Luminol (100 µL of 2.0 mg/mL solution in 10.0 mM NaOH) + H_2O_2 (100 µL of 0.15%).

Table S5a. Chemiluminescence data for compound **8**.

**Trial**	**Chemiluminescence Intensity**	
	**Cobalt-gel+ Luminol +H_2O_2**	**Copper gel+ Luminol +H_2O_2**
1	2090000	3555000
2	1730000	3408000
3	2057000	3210000
**Average**	1959000	3391000
**SD%**	8.294381	4.168614

Table S5b. Chemiluminescence data for compound **11**.

**Trial**	**Chemiluminescence Intensity**				
	**Co-gel + Luminol +H_2O_2**	**Cu-gel + Luminol +H_2O_2**	**Ni-gel + Luminol +H_2O_2**	**Fe-gel + Luminol +H_2O_2**	**Zn-gel + Luminol +H_2O_2**
1	5666000	18730000	4421000	167100	7656000
2	5794000	20290000	5824000	164500	8370000
3	5557000	19110000	5160000	190654	6282800
**Average**	5672333	19376667	5135000	174085	7436267
**SD %**	2	4	13.7	8	14

6. Drug loading and release studies

In a one dram vial, compound **8** (10.0 mg) and 0.1 mg of Vitamin B_2 were dissolved in 1.0 mL of DMSO:H_2O (1:2) mixture. The mixture was heated and sonicated. Gel formation was observed at 10.0 mg/mL. The final gel was diluted with DMSO:H_2O (1:2) mixture in 0.1 mL increments. The minimum gelation concentration was found to be 3.8 mg/mL after dilution.

In a one dram vial, compound **11** (10 mg) and 0.5 mg of Naproxen sodium were dissolved in 1.3 mL DMSO:H_2O (1:10) mixture to give a gel of 7.7 mg/mL. The mixture was heated and sonicated to form an opaque gel.

Naproxen release from the co-gels using compound **11**.

A gel was prepared in a one-dram vial using compound **11** (15.0 mg) and 0.5 mg of naproxen sodium, and 2.0 mL of DMSO/H_2O (v/v 1:10). After a stable gel formed and the gel was left undisturbed for 15 min, 2.0 mL of DMSO/H_2O (v/v 1:10) was added to the top of the gel carefully. Naproxen release from the gel was monitored by UV absorption at intervals by transferring the supernatant with a pipet to a cuvette, and after each measurement, the aqueous phase was carefully transferred back to the vial and placed on top of the gel again until the next measurement. The UV spectra of 0.5 mg pure naproxen in 4.0 mL DMSO/H_2O (v/v 1:10) was also recorded as standard. The images taken at various time points of gels loaded with naproxen are shown in Figure S6.

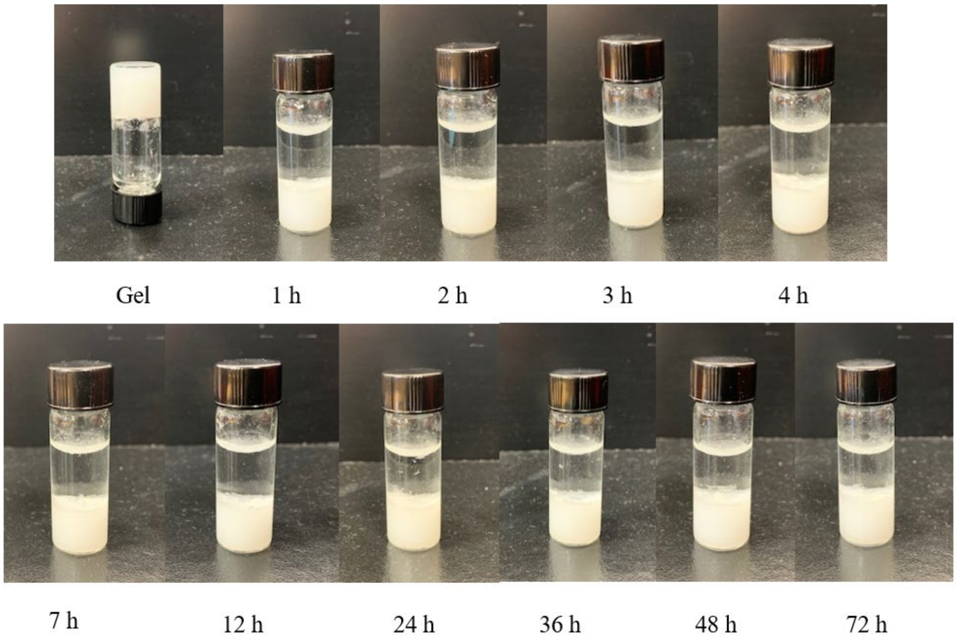

Figure S6. The gel images of compound **11** and naproxen in 2.0 mL of DMSO:H_2O (v/v 1:10).

7. Conductivity measurement of co-gels

Co-gels formed by compound **7** and TBABr were tested for conductivity using conductivity probe by Vernier with Cell Constant: 1.0 cm^{-1}. The probe has ABS body, parallel carbon (graphite) electrodes with dimensions of 12 mm OD and 150 mm length. The measurements were performed in high range: 0 to 20,000 µS/cm (0 to 10,000 mg/L Total Dissolved Solids) with accuracy using factory calibration: ±4% of full-scale reading for high range.

Step 1. Calibration curve for TBABr solutions

To obtain the calibration curve, TBABr solutions were prepared in DMSO:H_2O (1:9) using Millipore water ranging from 1.0 – 20.0 mM concentrations. The conductance of each solution was reported for a time span of 10 sec and the average reading was recorded and plotted in Figure S7, the graph shows linear relationship between the concentration and conductivity.

Concentration (mM)	Conductivity
20	1815
10	1014
5	631
2	372
1	282

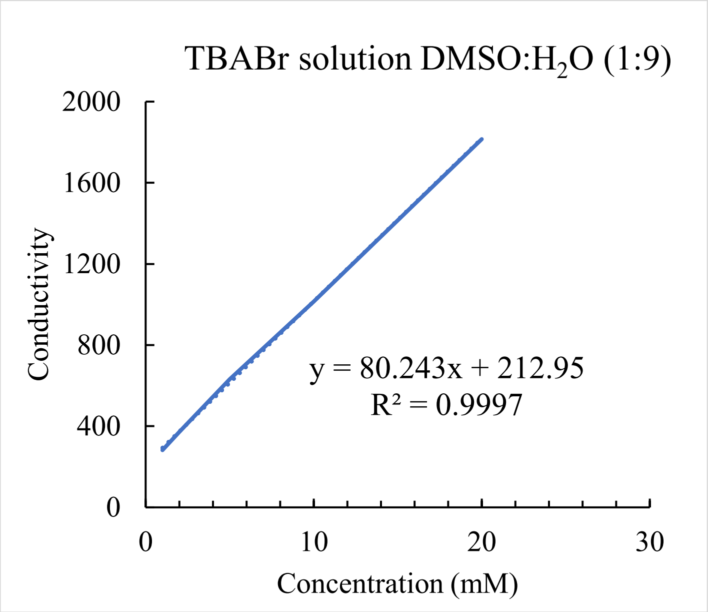

Figure S7. Conductivity correspondence to concentrations of TBABr solutions.

Step 2. Conductivity data for co-gels

To obtain the data, co-gels were prepared using compound **7** and TBABr in various ratios in DMSO:H_2O (1:9) using Millipore water. The conductivity of each gel and solution was recorded for a time span of 10 sec and the average reading was reported. Estimated values of TBABr solutions were obtained from the calibration curve shown in Step 1 above. The bar graphs of conductivities of the co-gels formed by compound **7** in the presence of TBABr electrolytes (blue) and the TBABr solutions (orange) are shown in Figure S8.

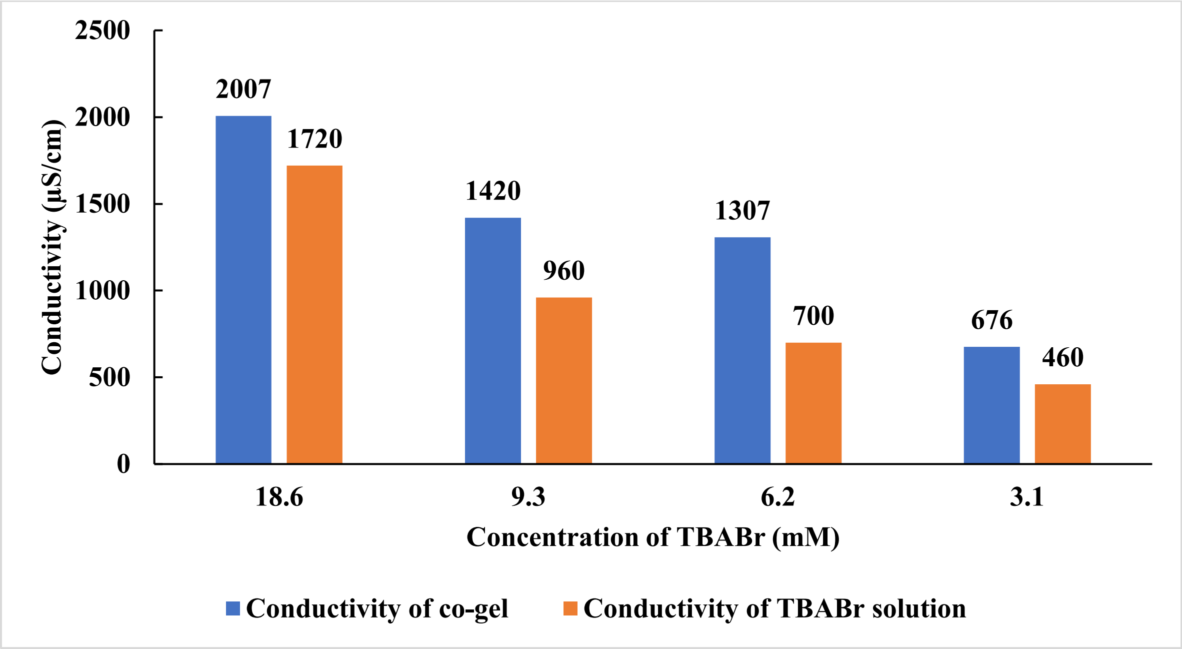

Figure S8. Conductivities of the co-gels formed by compound **7** in the presence of TBABr and the TBABr solutions.

Table S6. Conductivity data of multicomponent gels.

**Compound 7**	**TBABr**	**Conductivity (μS/cm)**	**Molar Ratio of compound 7 with TBABr**
30 mg (control)	-	109	**-**
30 mg	10 mg (3.1 mM)	676	2:1
30 mg	20 mg (6.2 mM)	1307	1:1
30 mg	30 mg (9.3 mM)	1420	1:1.5
30 mg	60 mg (18.6 mM)	2007	1:3

8. LCMS Data

Compound 2 LC-MS (ESI+) calcd for $C_{18}H_{26}NO_7$ $[M + H]^+$ 368.1 found 368.

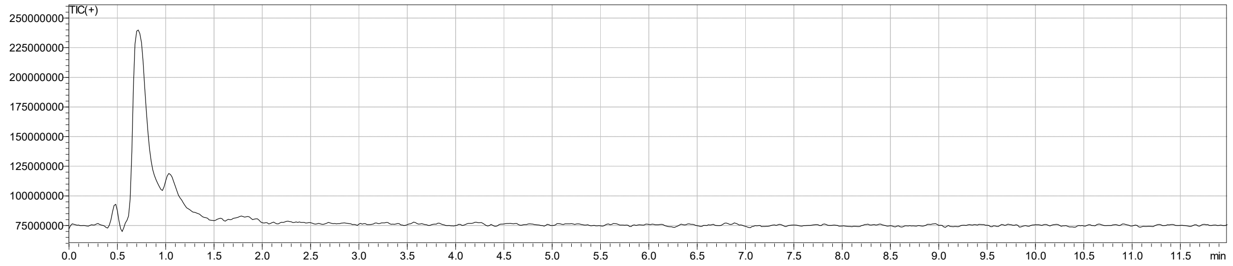

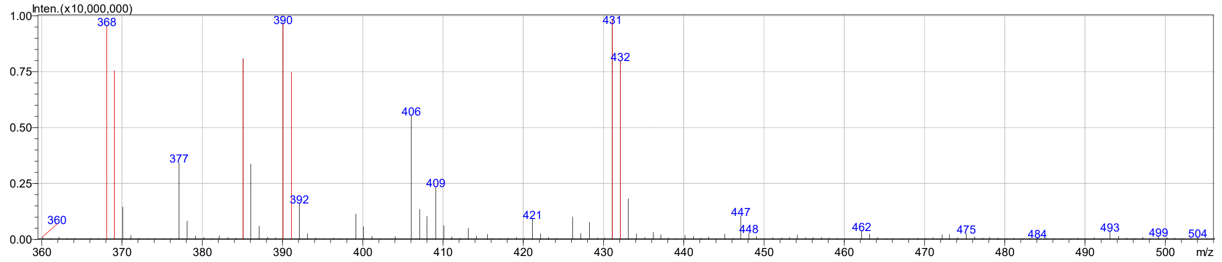

Compound 3 LC-MS (ESI+) calcd for $C_{19}H_{28}NO_7$ $[M + H]^+$ 382.1 found 382.

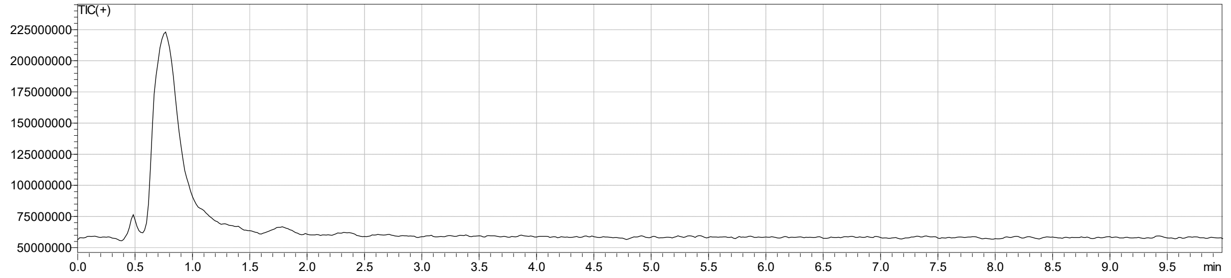

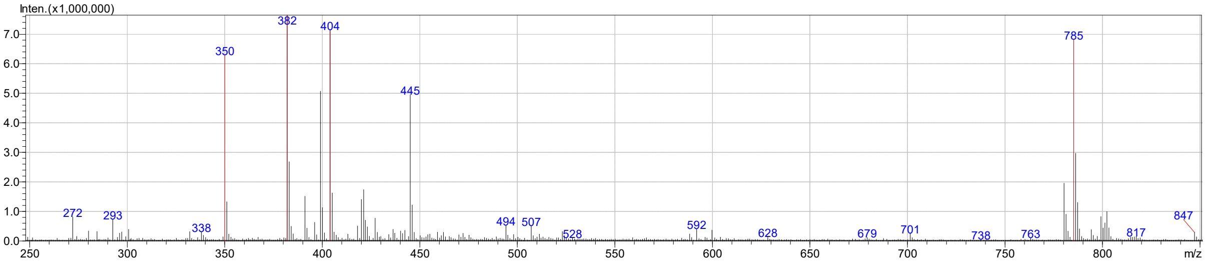

Compound 4 LC-MS (ESI+) calcd for $C_{19}H_{26}NO_7$ $[M + H]^+$ 380.1 found 380.

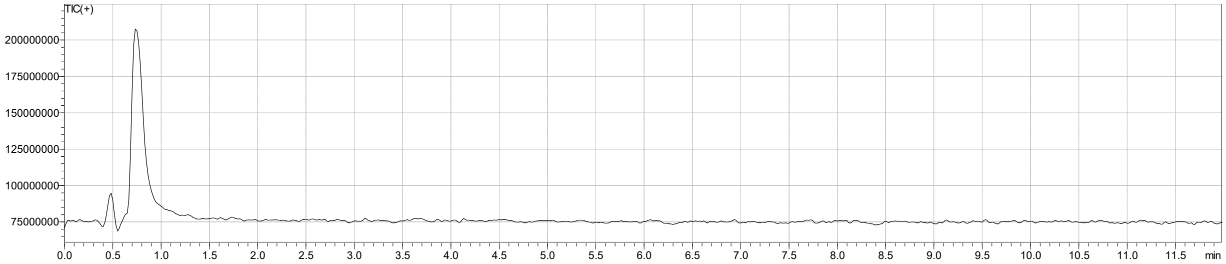

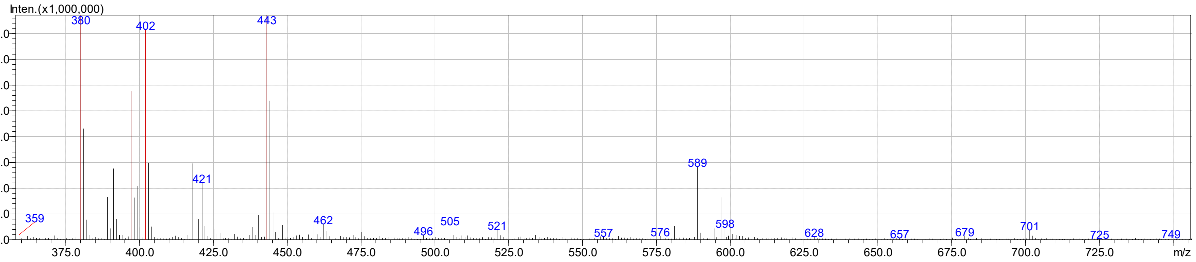

Compound 5 LC-MS (ESI+) calcd for $C_{20}H_{30}NO_7$ $[M + H]^+$ 396.1 found 396.

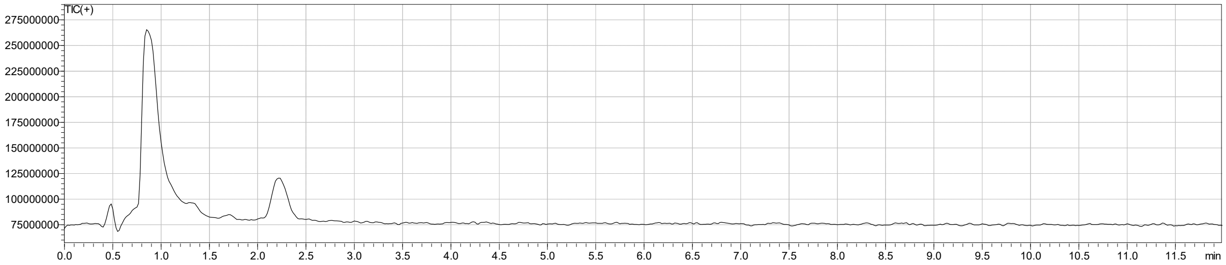

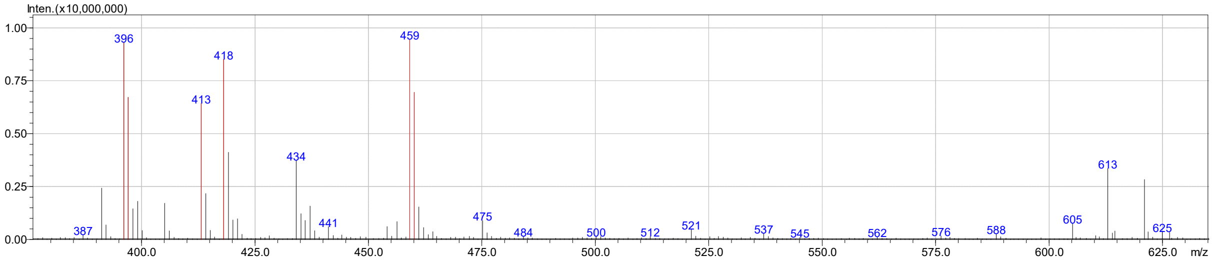

Compound 6 LC-MS (ESI+) calcd for $C_{24}H_{38}NO_7$ $[M + H]^+$ 452.2 found 452.

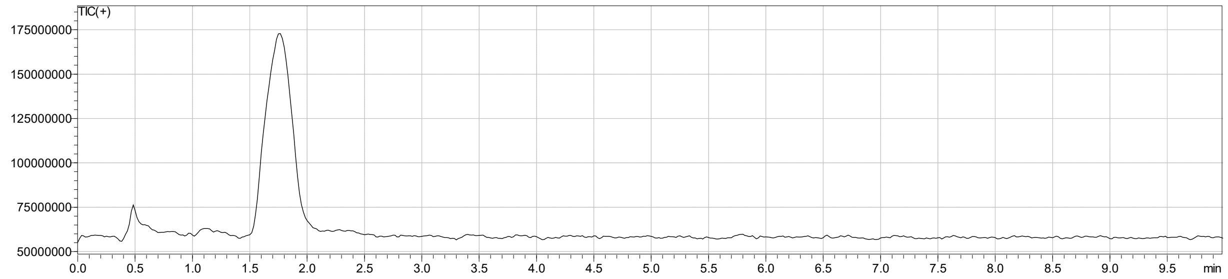

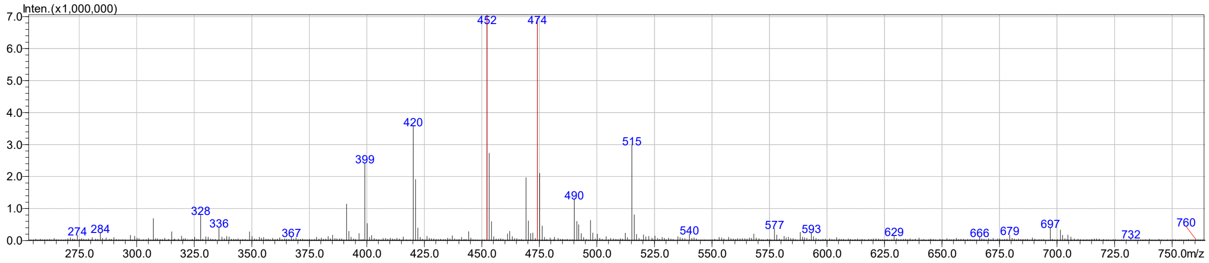

Compound 7 LC-MS (ESI+) calcd for $C_{19}H_{28}NO_7$ $[M + H]^+$ 382.1 found 382.

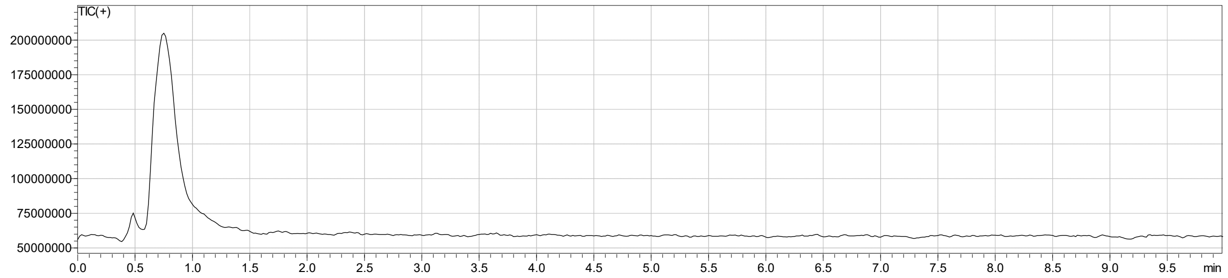

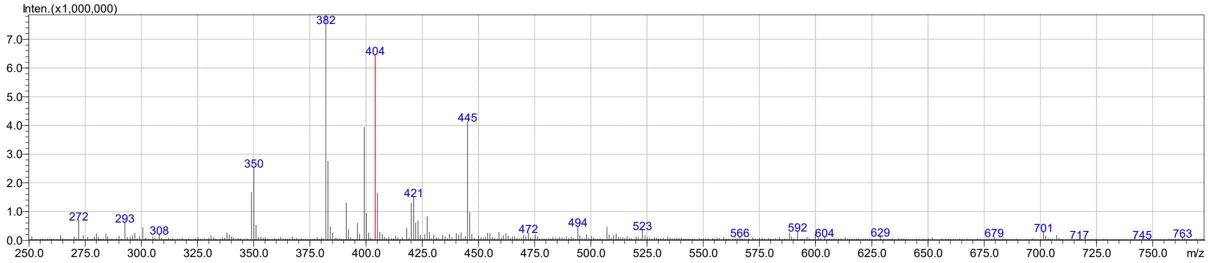

Compound 8 LC-MS (ESI+) calcd for $C_{20}H_{30}NO_7$ $[M + H]^+$ 396.1 found 396.

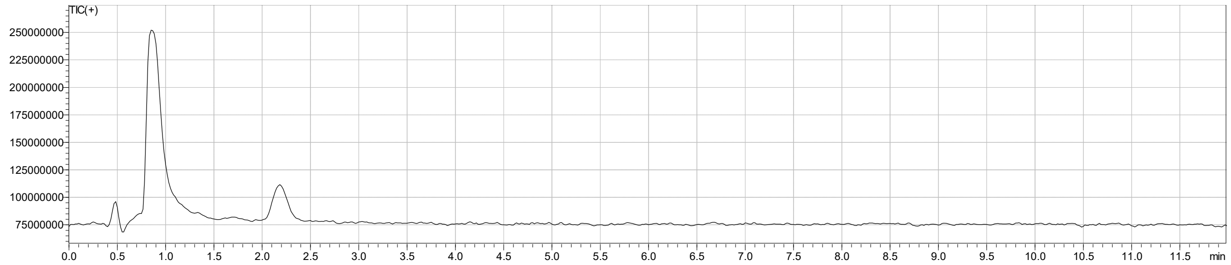

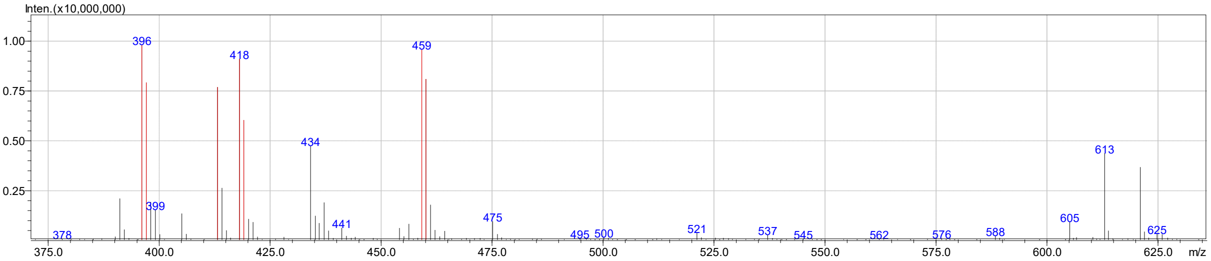

Compound 9 LC-MS (ESI+) calcd for $C_{18}H_{23}Cl_3NO_7$ $[M + H]^+$ 470.0, 472.0 found 470 and 472 (due to 35, 37 chlorine isotopes); and $[M+Na]^+$ 492, 494.

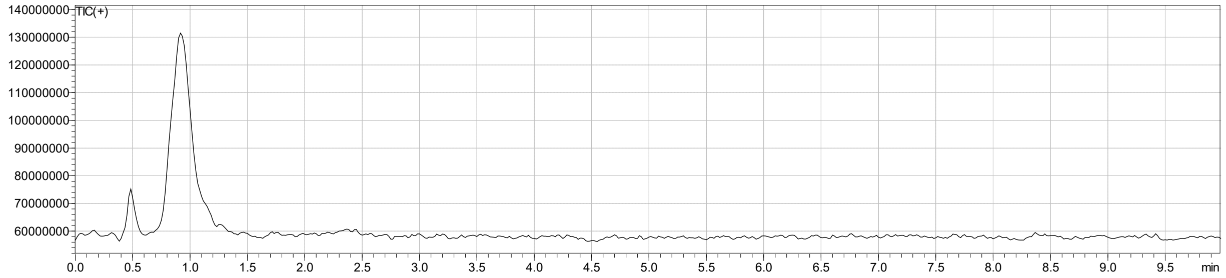

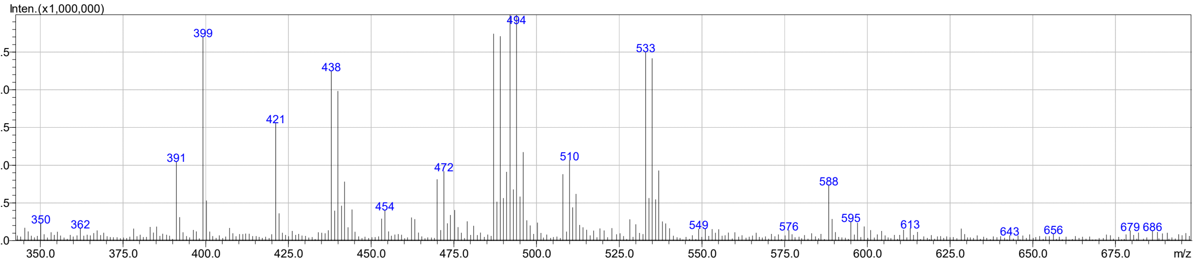

Compound 10 LC-MS (ESI+) calcd for $C_{22}H_{26}NO_7$ $[M + H]^+$ 416.1 found 416.

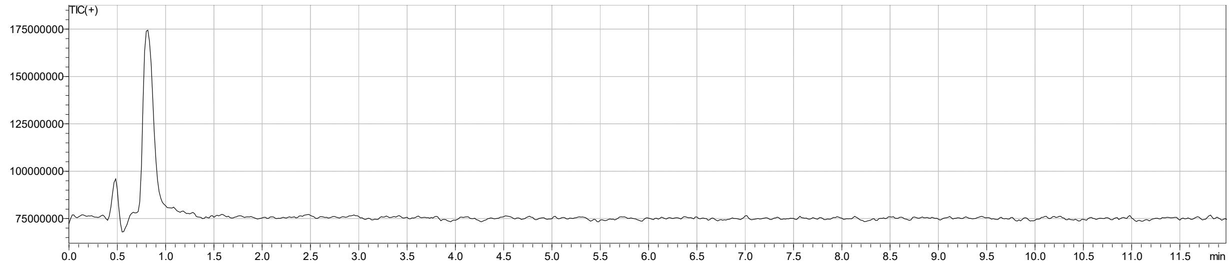

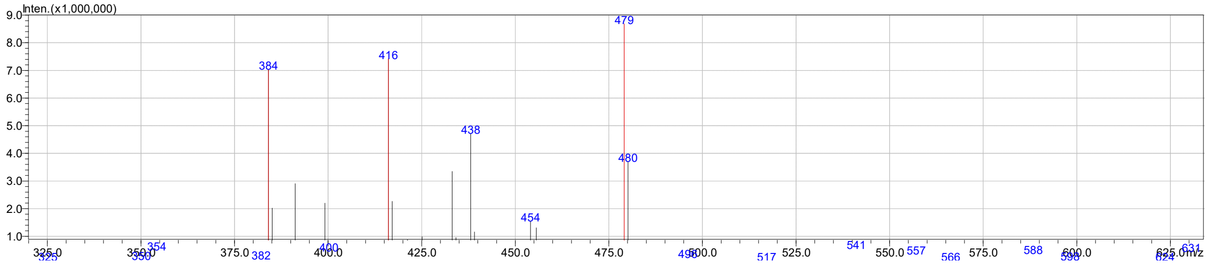

Compound 11 LC-MS (ESI+) calcd for $C_{23}H_{28}NO_7$ $[M + H]^+$ 430.1 found 430.

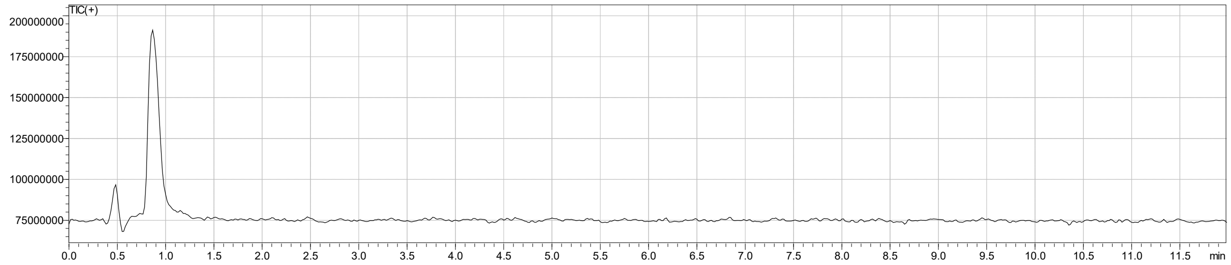

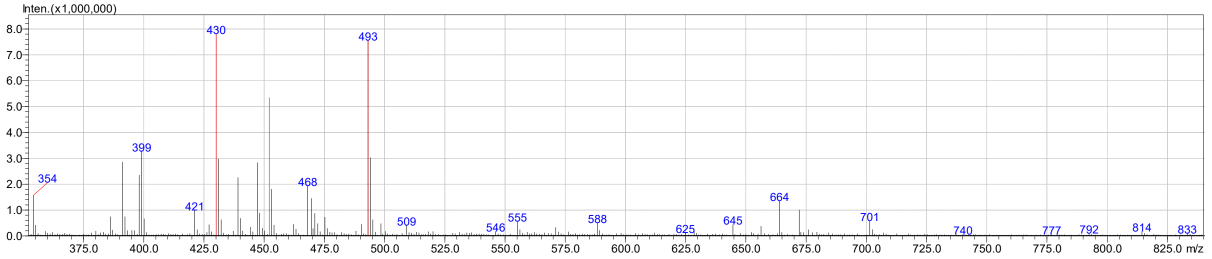

Compound 12 LC-MS (ESI+) calcd for $C_{23}H_{27}N_2O_9$ $[M + H]^+$ 475.1 found 475.

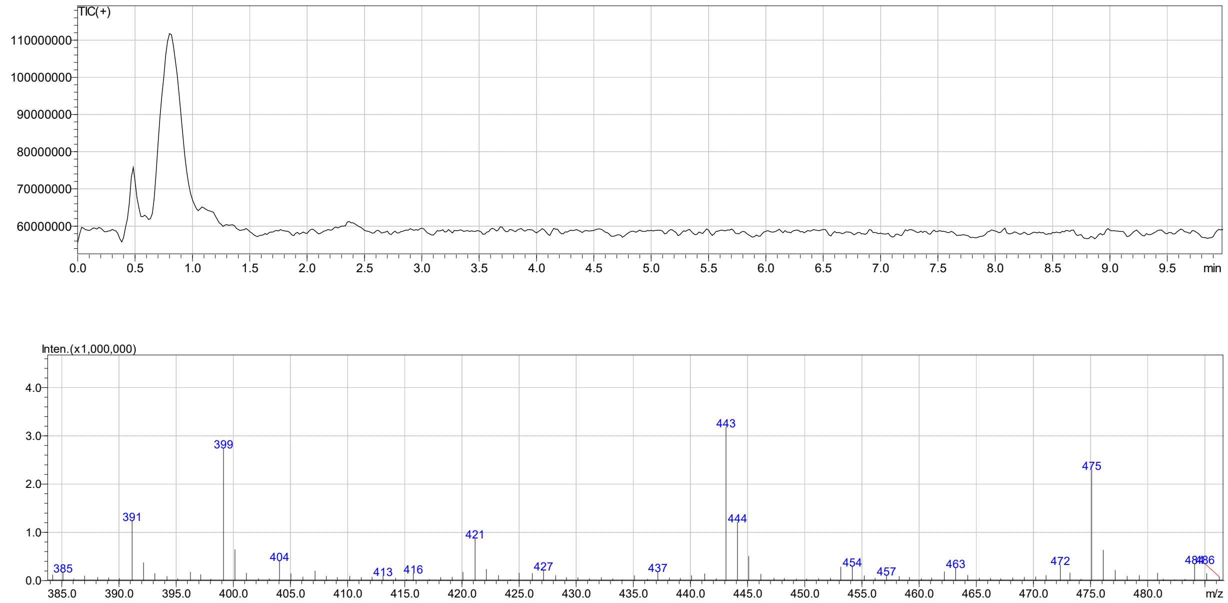

Compound 13 LC-MS (ESI+) calcd for $C_{22}H_{25}ClNO_7$ $[M + H]^+$ 450.1 found 450.

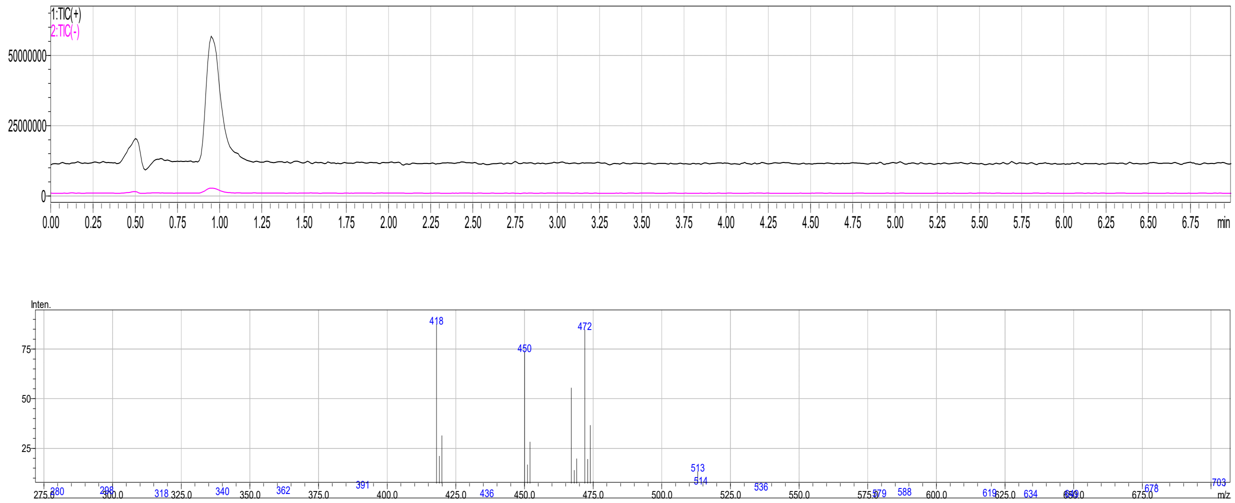

Compound 14 LC-MS (ESI+) calcd for $C_{30}H_{32}NO_7$ $[M + H]^+$ 518.2 found 518.

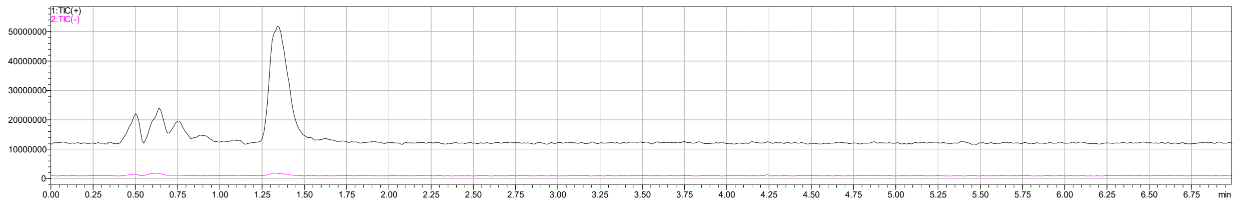

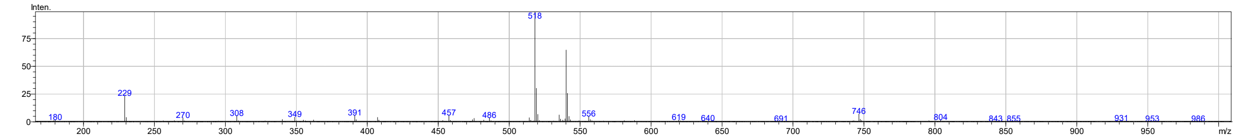

Supplement: Supplementary file 1 [file gels-08-00191-s001.zip › gels-1630106-SI.pdf]
